# Cross-protection against highly pathogenic avian influenza H5N1 virus from seasonal influenza vaccines: a systematic review and meta-analysis of ferret studies

**DOI:** 10.1080/22221751.2026.2654278

**Published:** 2026-04-15

**Authors:** IShin Tseng, Ying-Chi Huang, Wei-Liang Shih, Chung-Hsi Chou, Sten H. Vermund, Chi-Tai Fang

**Affiliations:** a Institute of Epidemiology and Preventive Medicine, College of Public Health, National Taiwan University, Taipei, Taiwan; b National Institute of Infectious Diseases and Vaccinology, National Health Research Institutes, Zhunan, Taiwan; c Infectious Diseases Research and Education Center, Ministry of Health and Welfare and National Taiwan University, Taipei, Taiwan; d Zoonoses Research Center and School of Veterinary Medicine, National Taiwan University, Taipei, Taiwan; e Office of the Dean, University of South Florida College of Public Health, Tampa, FL, USA; f Department of Pediatrics, University of South Florida Morsani College of Medicine, Tampa, FL, USA; g Global Virus Network, Tampa, FL, USA; h Department of Internal Medicine, National Taiwan University Hospital, Taipei, Taiwan; i National Taiwan University School of Medicine, Taipei, Taiwan

**Keywords:** Avian influenza, cross-protection, seasonal influenza vaccines, neutralizing antibody, pandemic preparedness, ferrets

## Abstract

The recent surge in spillover events of highly pathogenic avian influenza A(H5N1) clade 2.3.4.4b to humans and mammals in North America has raised urgent pandemic concerns. Human H5N1 vaccines are unavailable in most countries. We synthesized data from ferret challenge trials to evaluate whether widely available seasonal influenza vaccines confer cross-protection against lethal H5N1 infection. We systematically searched PubMed, Embase, and Web of Science for ferret studies of lethal H5N1 challenge published up to 5 July 2025 (PROSPERO #CRD42024520346). Random-effects meta-analyses were conducted to compare vaccine efficacy (VE) of seasonal influenza vaccines and H5N1 vaccines against H5N1-related mortality. Seroprotection was defined as a neutralizing antibody titre of ≥1:40. We identified 35 studies (157 trials). Seasonal influenza vaccines without N1 did not confer significant cross-protection (five trials; VE 14.8%, 95% CI –3.6 to 30.0). In contrast, VE was 73% for N1-containing seasonal influenza vaccines (19 trials; 95% CI 54–84) and 77% for H5N1 vaccines overall (133 trials; 95% CI 72–82) (*p* = 0.52). The VE of N1-containing seasonal influenza vaccines was modestly lower than that of H5N1 vaccines with seroprotection (88%; 66 trials; 95% CI 84–91; *p* = 0.009), but comparable to H5N1 vaccines that did not achieve seroprotection (63%; 67 trials; 95% CI 52–71; *p* = 0.29). The VE of seasonal influenza vaccines against H5N1 was robust across sensitivity analyses, with no evidence of publication bias (*p* = 0.99). Seasonal influenza vaccines significantly reduce H5N1-associated mortality in ferret trials, suggesting the cross-protection potential of currently available vaccines. Human studies are warranted.

## Introduction

The recent surge in spillover of highly pathogenic avian influenza (HPAI) A(H5N1) clade 2.3.4.4b virus to mammals in North America has raised global alarm [1]. In addition to sporadic transmission from avian species to carnivores [2], sustained mammal-to-mammal transmission has occurred among cattle across 18 U.S. states, as of 5 October 2025 [3,4]. Reports have also documented cases of cattle-to-human transmission and bird-to-human transmission, including one fatality, underscoring the zoonotic potential of this virus [5]. The possibility of human-to-human transmission is becoming increasingly concerning. A recent U.S. study found that three of 150 surveyed bovine veterinarians had serological evidence of recent infection despite no known exposure to infected cattle [6]. Given the historically high fatality rates of H5N1 infection in humans [7], the spillover of H5N1 clade 2.3.4.4b into human populations could initiate a global pandemic with serious consequences [8].

The availability of an effective H5N1 vaccine is critical to prevent severe disease and mortality during a future H5N1 pandemic [9]. However, H5N1 vaccines have been comparatively less immunogenic in humans, typically requiring the use of aluminum [10] or MF59 adjuvants [11]. Several H5N1 vaccines have completed phase 3 clinical trials using immunogenicity endpoints [12], yet to date, only Finland has initiated H5N8 vaccination for persons at increased risk [13]. Although some nations have stockpiles, no human vaccine specific for the prevention of HPAI H5N1 is commercially available in the United States or elsewhere [14]. mRNA-based vaccines offer the advantage of rapid production without the need for egg-adapted seed strains [15]. Nevertheless, on 5 August 2025, the United States federal government announced the cancellation of its H5 mRNA vaccine development programme [16].

Given the limited availability of H5N1 vaccines and their associated safety concerns during the pre-pandemic stage, alternative strategies, such as leveraging cross-protection from seasonal influenza vaccines, are being explored. *In vivo* mouse studies suggest that seasonal influenza vaccination provides cross-protection against H5N1 via H5 cross-reactive antibodies [17]. Seasonal influenza vaccination has been shown to elicit cross-protective antibodies against H5N1 in 2.5% to 15% of non-elderly adults [18], and to boost titres to seroprotective levels in individuals with pre-existing H5N1 antibodies [19]. (In recent years, the human seasonal influenza vaccine has been trivalent or quadrivalent (2013–2024): an influenza A (H1N1) virus, an influenza A (H3N2) virus, and one or two influenza B lineage viruses. For example, in the 2010–2011 season, the product contained A/California/7/2009 (H1N1)-like, A/Perth/16/2009 (H3N2)-like, and B/Brisbane/60/2008-like viruses, while in 2025–2026, it had A/Victoria/4897/2022 (H1N1) pdm09-like, A/Croatia/10136RV/2023 (H3N2)-like, and B/Austria/1359417/2021 (B/Victoria lineage)-like viruses.) Seasonal influenza vaccines may confer cross-protection against H5N1, as demonstrated in an *ex vivo* human immunology study [20] and a ferret challenge study [21], through N1-targeted T cell-mediated immunity and antibody-dependent mechanisms. Potentially challenging is evidence that recent seasonal influenza vaccination can interfere with seroconversion following H5N1 vaccination, raising concerns about its impact on immune responses [22]. Human challenge trials, which have long been used to study the pathogenesis and transmission of seasonal influenza [23,24], would be required to provide definitive conclusions, but they pose insurmountable ethical and practical challenges because of the high fatality rate associated with H5N1 infection. Likewise, randomized clinical trials are not feasible given the low incidence of H5N1 outside pandemic conditions.

Ferrets, given their similarity to humans in influenza pathogenesis and transmission, have been established as the standard animal model for studying influenza viruses [25]. Challenge trials using ferret models offer an excellent alternative to human trials for evaluating the cross-protective efficacy of seasonal influenza vaccination against HPAI H5N1. However, past ferret challenge trials have had small sample sizes, limiting the generalizability of individual studies. Given that substantial uncertainty remains regarding the extent and consistency of cross-protection conferred by seasonal influenza vaccines against H5N1, we conducted a systematic review and meta-analysis of mortality data from controlled ferret challenge trials to determine the extent of cross-protection provided by seasonal influenza vaccines against HPAI, compared with that of H5N1 vaccines.

## Methods

### Search strategy and selection criteria

Our systematic review and meta-analysis were conducted in accordance with the Preferred Reporting Items for Systematic Reviews and Meta-Analyses (PRISMA) guidelines. The study protocol, part of a systematic review and meta-analysis project on vaccines against HPAI H5N1 across experimental animal models, was prospectively registered in PROSPERO (CRD42024520346).

We searched PubMed, Embase, and Web of Science for articles published between 1 January 1997, and 5 July 2025, that reported lethal HPAI H5N1 challenge trials in ferrets assessing vaccine efficacy (VE), defined here as reduction in H5N1-associated mortality, for seasonal influenza or H5N1 vaccines, using placebo controls as the comparison group and with no language restrictions. The following search string was applied to article titles: [(avian influenza) OR (bird flu) OR (HPAI) OR (H5N1) OR (H5Nx) OR (influenza A)] AND [(efficacy) OR (protect) OR (protection) OR (immunization) OR (prevent)] AND [(vaccine) OR (vaccination) OR (hemagglutinin)]. Full search strategies are provided in Supplementary Table 1. This query was applied uniformly across all databases. Records were excluded if they were duplicates, meta-analyses, reviews, reader comments, or protocols.

Two reviewers (IST and YCH) independently conducted the literature search, applied the inclusion and exclusion criteria, identified eligible trials from each article, and assessed the risk of bias using the Collaborative Approach to Meta-analysis and Review of Animal Data in Experimental Studies (CAMARADES) checklist [26]. Because multiple trials within a study shared identical methodology and conditions, quality was assessed at the study level. Any discrepancies, including continuous variables with a difference exceeding 5%, were resolved through discussion with a third reviewer (CTF).

We compared VE against lethal H5N1 challenge between seasonal influenza vaccines and H5N1 vaccines. We included studies if they were experimental trials conducted in ferrets that compared mortality between vaccinated and placebo groups (unvaccinated, saline/PBS control, or vector control) following lethal H5N1 challenge, with at least 50% mortality in the placebo group. We imposed no restriction on the type of seasonal influenza or H5N1 vaccine and treated commercially available formulations and vaccines prepared for experimental use equivalently. Studies were excluded if the challenge strain was not H5N1, if the challenge dose was sublethal or could not be determined because of missing survival data, or if the vaccine strain was not H5N1, H1N1, H3N2, or influenza B. For publications that included multiple trials using different challenge doses, trials with sublethal challenge were excluded.

### Data extraction

Data from eligible trials (experiments) were extracted using a standardized form. Extracted variables comprised: (1) study characteristics (first author, publication year, and number of trials per publication); (2) experimental animal characteristics (baseline influenza serostatus and sample size per trial); (3) intervention characteristics (vaccine type [seasonal influenza or H5N1] and dosing regimen [priming or booster]); (4) challenge H5N1 clade/strain; (5) time interval (days) from the last dose of vaccination to challenge; and (6) outcome measures (mortality counts and post-vaccination geometric mean titres [GMTs]). When outcome data were presented only in graphical format, values were digitized using WebPlotDigitizer (https://automeris.io/WebPlotDigitizer).

### Outcome measures

The primary outcome was mortality, defined as death from H5N1 infection or euthanasia performed for very severe illness following H5N1 challenge. The secondary outcome was seroprotection against H5N1 – measured using the challenge strain, vaccine strain, or a heterologous strain as the test strain – after study vaccination, defined as a neutralizing antibody titre of ≥1:40 by hemagglutination inhibition (HI) or microneutralization (MN) assays. When multiple HI time points were reported, the pre-challenge measurement closest to challenge was extracted. If HI data were unavailable, MN results were used. Antibody measurements were prioritized in the following order: HI with horse red blood cells, HI with chicken red blood cells, and MN with Madin–Darby canine kidney cells.

### Meta-analytic framework

VE against mortality following H5N1 challenge was estimated by comparing mortality risk between vaccinated and control groups. VE was calculated as 1 − RR, where RR is the mortality risk ratio. Trials from the same publication were treated as separate observations when they represented distinct experimental comparisons with different vaccine regimens or challenge conditions. Because substantial clinical and methodological heterogeneity across ferret challenge studies was anticipated, we used a random-effects meta-analysis with the Mantel–Haenszel method and further explored heterogeneity through prespecified subgroup and sensitivity analyses.

To examine the role of N1-targeted immunity in cross-protection, subgroup analyses of seasonal influenza vaccines were conducted according to whether the study vaccines contained the N1 antigen. Because immunogenicity varied substantially across H5N1 vaccine studies, subgroup analyses of H5N1 vaccines were performed according to whether the study vaccines achieved seroprotection. Differences in pooled VE estimates between vaccine categories were assessed by testing for subgroup differences within a random-effects model using Cochran’s Q statistic. Relative efficacy was estimated through a frequentist network meta-analysis with a random-effects model.

Heterogeneity was assessed using the I² statistic. To explore potential sources of heterogeneity, additional subgroup analyses were conducted according to challenge H5N1 clade, vaccine platform (virus-like particle [VLP], DNA, inactivated, or live vaccines), adjuvant use, booster use, and study period (before versus after the 2009 H1N1 pandemic, for N1-containing seasonal influenza vaccines).

To evaluate the robustness of the estimated cross-protective efficacy of seasonal influenza vaccines, we performed a leave-one-out sensitivity analysis.

Because sample sizes in animal experiments are constrained to a narrow range under the Replacement, Reduction, and Refinement (3R) principles, conventional methods for detecting publication bias, such as funnel plots and Egger’s test, were not considered appropriate. Instead, we evaluated potential small-study effects, including possible publication bias, by testing whether VE varied systematically according to trial arm size. Subgroup differences were assessed using Cochran’s Q statistic across sample-size categories: small (≤4 animals per arm), medium (5–9 animals per arm), and large (10–12 animals per arm). The certainty of evidence (CoE) was evaluated using the GRADE approach [27].

### Statistical analysis

All statistical analyses were performed using R version 4.0.3 (R Foundation for Statistical Computing, Vienna, Austria) with the meta and netmeta packages. Statistical significance was defined as a two-sided *p* value of less than 0.05.

## Results

**Figure 1.**
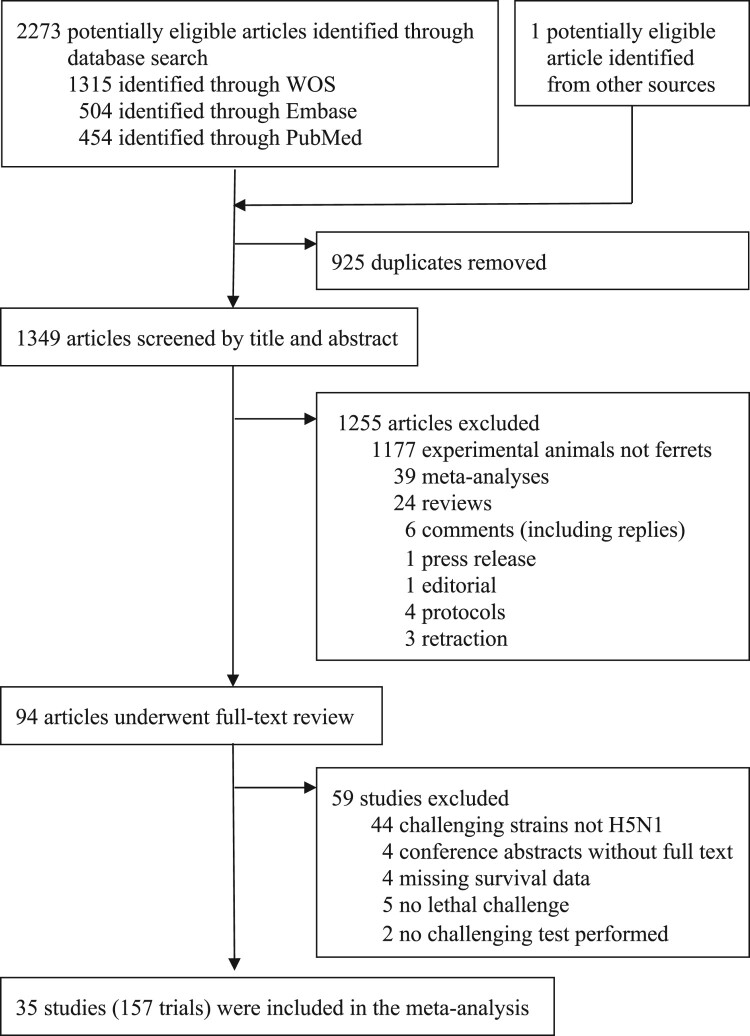
**Study selection**. Of 2,274 potentially eligible items identified, 1,349 were screened by title and abstract after removing duplicates and non-original research articles. Of these, 1,255 were excluded for not involving experimental trials or for using animal models other than ferrets. After full-text review, 59 of the remaining 94 studies were excluded, with reasons listed in Supplementary Table 2. In total, 35 studies comprising 157 eligible trials were included in the meta-analysis (Supplementary Table 3). WOS = Web of Science.

We identified 2,274 potentially eligible studies (Figure 1). After removal of duplicates and screening of titles and abstracts, 94 articles underwent full-text review, of which 59 were excluded for reasons listed in Supplementary Table 2, including four conference abstracts without available full text. We included 35 studies reporting 157 eligible trials (Supplementary Table 3). These trials involved a total of 911 ferrets in the vaccinated groups and 880 ferrets in the placebo control groups. Trials were conducted between 2005 and 2024, most commonly in 2009 (40 trials) and 2012 (20 trials).

Among the 157 trials, 24 evaluated vaccines derived from human strains, and 133 evaluated vaccines derived from avian strains, all of which were H5N1 or included an H5N1 valency.

Seasonal influenza vaccine types included inactivated vaccines (whole-virus, split-virion, or subunit; 15 trials), live vaccines (wild-type or attenuated; 4), VLP vaccines (3), and DNA vaccines (2). H5N1 vaccine types included recombinant subunit vaccines (58 trials), inactivated vaccines (whole-virus or split-virion; 46), VLP vaccines (11), DNA vaccines (9), mRNA vaccines (7), and live wild-type H5N1 vaccines (2).

The challenge H5N1 viruses belonged to A/Vietnam/1203/2004 (clade 1; 93 trials), A/Indonesia/05/2005 (clade 2.1; 26), A/Vietnam/1194/2004 (clade 1; 17), A/bald eagle/Florida/22–006544–004/2022 (clade 2.3.4.4b; 8), A/EM/Korea/W149/2006 (clade 2.2; 6), A/Whooper Swan/Mongolia/244/2005 (clade 2.2; 3), A/Egypt/2321–NAMRU3/2007 (clade 2.2.1; 2), A/Cygnus cygnus/Germany/R65/2006 (clade 2.2; 1), and A/bald eagle/Florida/W22-134-OP/2022 (clade 2.3.4.4b; 1). The interval between vaccination and challenge ranged from 7 to 140 days, with most challenges occurring 14–28 days after vaccination.

**Figure 2.**
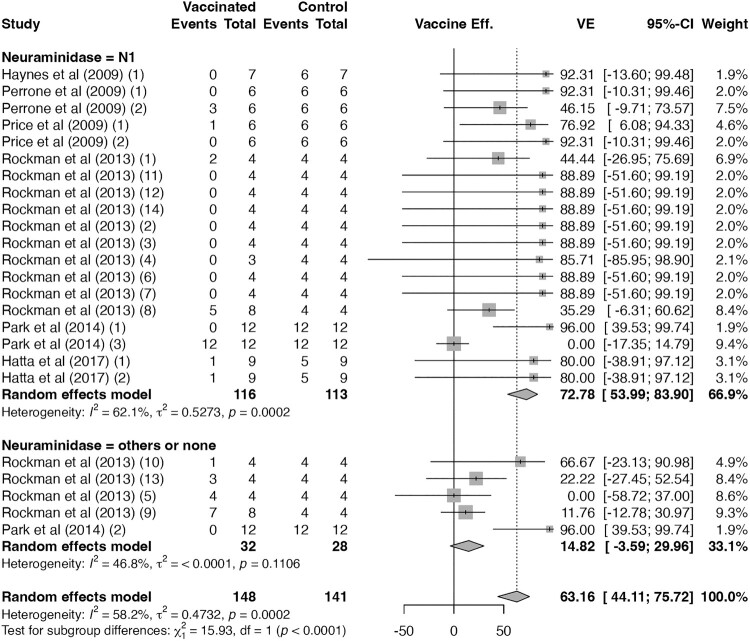
**Vaccine efficacy against H5N1-related mortality for seasonal influenza vaccines.** Forest plot showing vaccine efficacy among seasonal influenza vaccine trials, stratified by whether the vaccine contained the N1 antigen. One published study may report multiple trials, which were labelled in the order described in the article with numbers in parentheses. Diamonds represent pooled estimates under random-effects models; error bars indicate 95% confidence intervals.

The VE of seasonal influenza vaccines differed according to the presence of N1 antigens (Figure 2). Seasonal influenza vaccines lacking the N1 antigen – including monovalent H3N2 vaccine (three trials), purified H1 antigen (one), and purified H3 antigen (one), all specifically prepared for experimental use – did not confer significant cross-protection against H5N1 (VE 14.8%, 95% CI –3.6 to 30.0; I² = 47%). In contrast, N1-containing seasonal influenza vaccines (VE 73%, 95% CI 54–84; I² = 62%; CoE: moderate; Figure 2) showed protection slightly lower than that of H5N1 vaccines overall (VE 77%, 95% CI 72–82; I² = 54%; CoE: moderate; *p* = 0.52; Supplementary Figure 1).

None of the seasonal influenza vaccines achieved H5N1 seroprotection. In contrast, 66 (50%) of the 133 H5N1 vaccine trials achieved seroprotection. H5N1 vaccine trials that included booster doses were substantially more likely to achieve seroprotection than priming-only trials (55.1% [65 of 118] vs 6.7% [1 of 15]; *p* = 0.002). The likelihood of seroprotection did not differ by test strain (*p* = 0.82) or by assay method (*p* = 0.53) (Supplementary Results).

**Figure 3.**
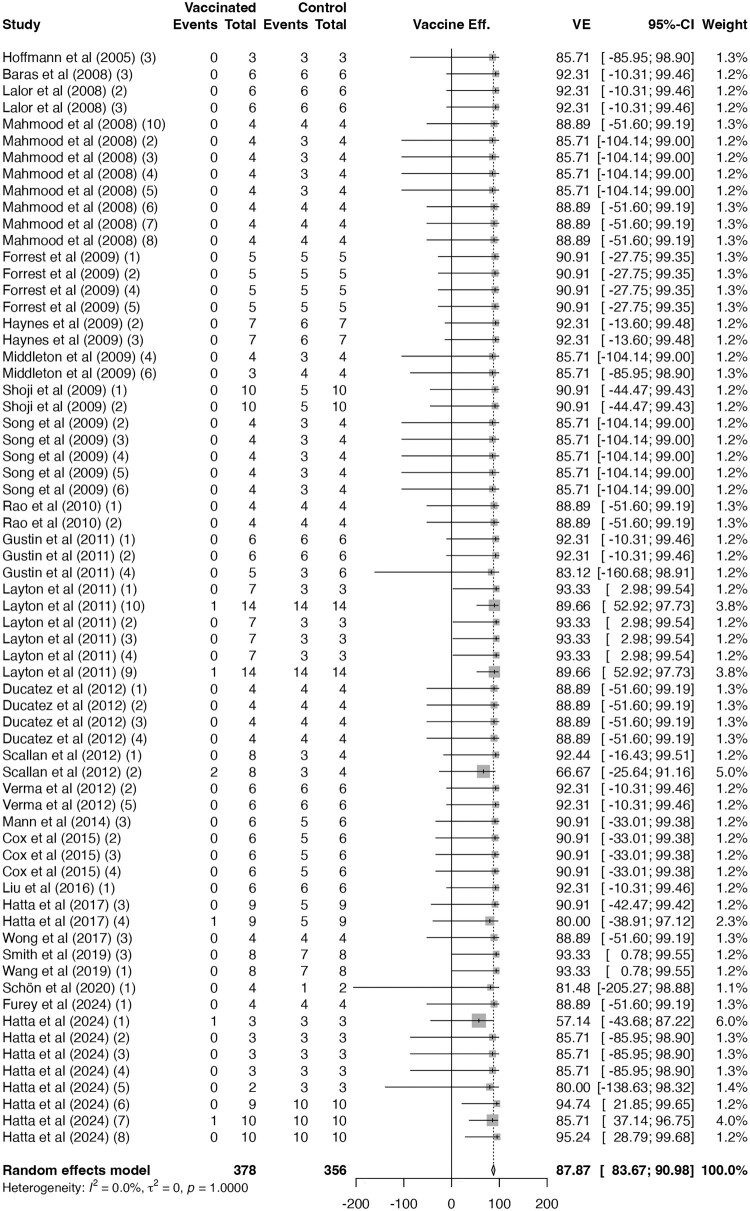
**Vaccine efficacy against H5N1-related mortality for H5N1 vaccines with seroprotection.** Forest plot showing vaccine efficacy among H5N1 vaccine trials that achieved seroprotection. One published study may report multiple trials, which were labelled in the order described in the article with numbers in parentheses. Diamonds represent pooled estimates under random-effects models; error bars indicate 95% confidence intervals.

**Figure 4.**
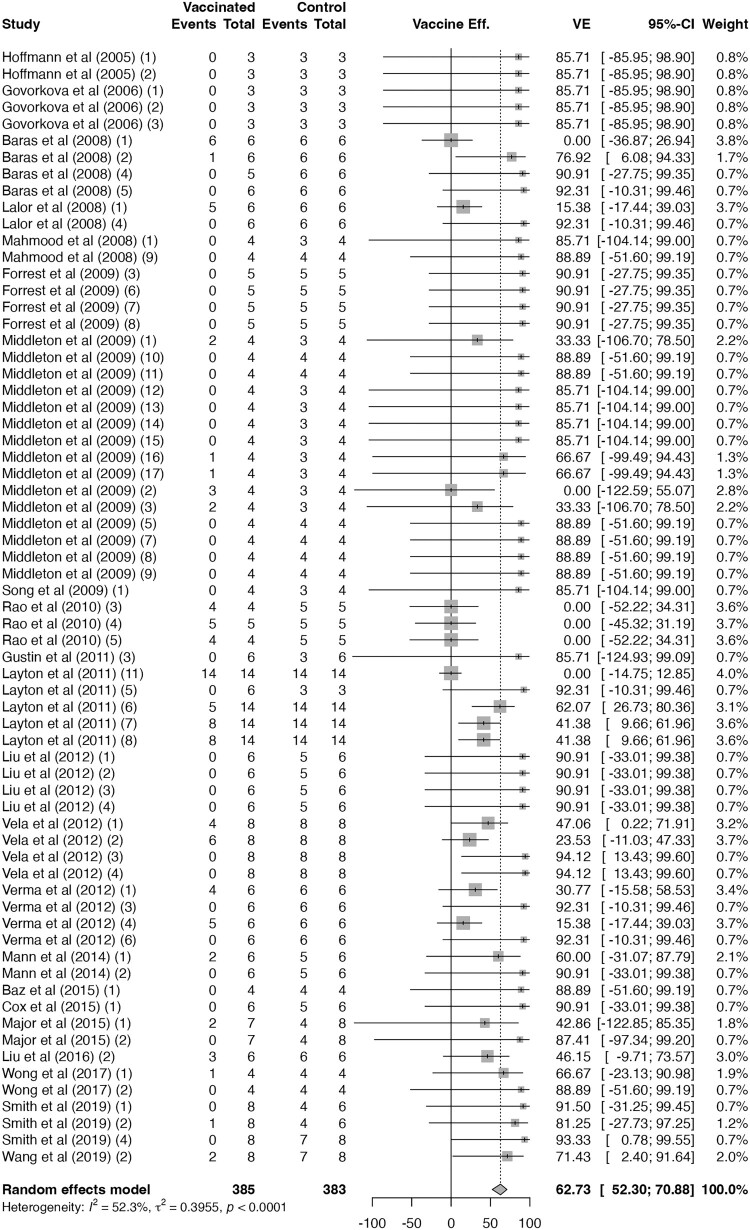
**Vaccine efficacy against H5N1-related mortality for H5N1 vaccines without seroprotection.** Forest plot showing vaccine efficacy among H5N1 vaccine trials that did not achieve seroprotection. One published study may report multiple trials, which were labelled in the order described in the article with numbers in parentheses. Diamonds represent pooled estimates under random-effects models; error bars indicate 95% confidence intervals.

The pooled VE of H5N1 vaccines with seroprotection were 88% (66 trials; 95% CI 84–91; I² = 0%; CoE: high; Figure 3), significantly higher than that of H5N1 vaccines without seroprotection (63%; 67 trials; 95% CI 52–71; I² = 52%; CoE: moderate; Figure 4; *p* < 0.01; Supplementary Figure 2). However, among H5N1 trials with seroprotection, no difference was observed across subgroups using different numbers of boosters (*p* = 0.79; Supplementary Figure 3), indicating that once seroprotection was achieved, additional boosters did not further improve VE.

N1-containing seasonal influenza vaccines had a VE of 73% (19 trials; 95% CI 54–84; I² = 62%; CoE: moderate; Figure 2), modestly lower than H5N1 vaccines with seroprotection (*p* < 0.01; Supplementary Figure 4) but comparable to those without seroprotection (*p* = 0.29; Supplementary Figure 5).

**Figure 5.**
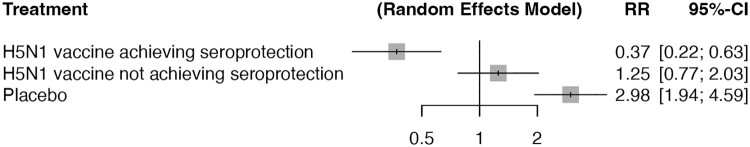
**Network meta-analysis of vaccine efficacy.** Relative risk of H5N1-related mortality after H5N1 challenge in ferrets vaccinated with H5N1 vaccines (stratified by seroprotection status) or placebo, relative to those vaccinated with seasonal influenza vaccines.

In a network meta-analysis using seasonal influenza vaccination as the reference (Figure 5), H5N1 vaccination with seroprotection was associated with a substantially lower risk of H5N1-associated mortality (RR = 0.37, 95% CI 0.22–0.63), whereas H5N1 vaccination without seroprotection conferred no additional benefit compared with seasonal influenza vaccination (RR = 1.25, 95% CI 0.77–2.03). Placebo was associated with markedly higher mortality relative to seasonal vaccination (RR = 2.98, 95% CI 1.94–4.60).

Additional subgroup analyses showed that VE did not differ significantly across H5N1 challenge virus clades (*p* = 0.60; Supplementary Figure 6). For H5N1 vaccines, VE did not differ significantly by the interval from vaccination to challenge (<14 days, 89.5%; 14–28 days, 76.4%; >28 days, 75.2%; *p* = 0.10; Supplementary Figure 7). For N1-containing seasonal influenza vaccines, VE also did not differ significantly by the interval from vaccination to challenge (14–28 days, 72.0%; >28 days, 74.0%; *p* = 0.89; Supplementary Figure 8). Moreover, the VE of N1-containing seasonal influenza vaccines did not differ significantly by platform (VLP, 76.0%; DNA vaccines, 81.8%; inactivated vaccines, 66.5%; live vaccines, 85.6%; *p* = 0.62, Supplementary Figure 9), adjuvant use or booster use (*p* = 0.40 and *p* = 0.26, respectively; Supplementary Figures 10 and 11), or study period before versus after 2009 – when H1N1pdm09 emerged and was subsequently incorporated into seasonal influenza vaccines (76.3% vs. 72.0%, *p* = 0.77; Supplementary Figure 12).

To assess the robustness of these findings, we conducted leave-one-out sensitivity analyses. The pooled VE of N1-containing seasonal influenza vaccines varied between 73% (95% CI 51–83) and 76% (95% CI 57–89) (Supplementary Figure 13), and that of H5N1 vaccines with seroprotection varied between 87.8% (95% CI 83.5–90.9) and 88.5% (95% CI 84.4–91.5) (Supplementary Figure 14). The quality assessment of the 35 included studies yielded a median score of 5.6 (IQR 5–6) out of nine items (Supplementary Figures 15 and 16), with minimal differences between seasonal influenza vaccine studies and H5N1 vaccine studies (median 5.43 vs 5.66; *p* = 0.53).

**Figure 6.**
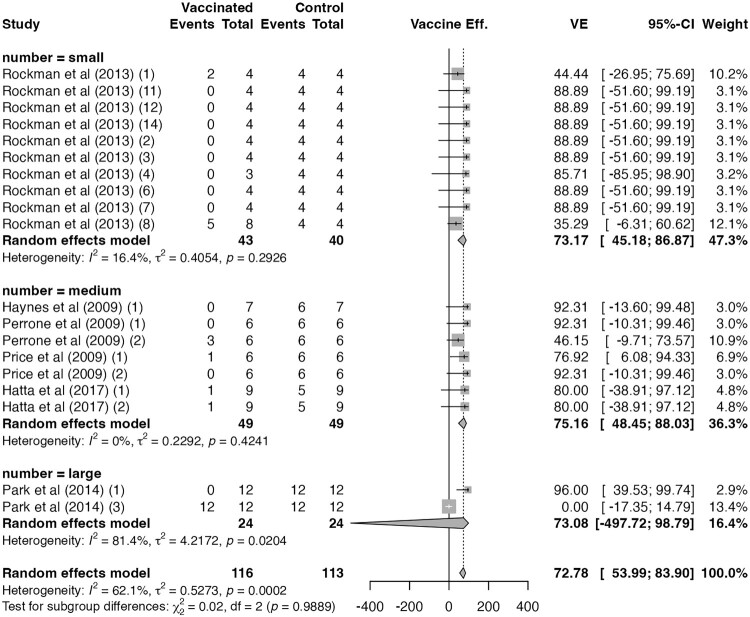
**Publication bias assessment of N1-containing seasonal influenza vaccine trials.** Forest plot showing vaccine effectiveness (VE) by trial arm sample size (small, medium, or large). Diamonds represent pooled estimates under random-effects models; error bars indicate 95% confidence intervals. No significant difference in VE was observed among subgroups (*p* = 0.99).

VE estimates did not differ across group-size categories for N1-containing seasonal influenza vaccines (*p* = 0.99; Figure 6) or for H5N1 vaccines with seroprotection (*p* = 0.27; Supplementary Figure 17). By contrast, among H5N1 vaccine trials without seroprotection, VE declined with increasing study size, raising concern about possible small-study effects, including potential publication bias (75% for ≤4 animals per arm, 63% for 5–9 per arm, and 35% for 10–12 per arm; *p* = 0.008; Supplementary Figure 18).

**Figure 7.**
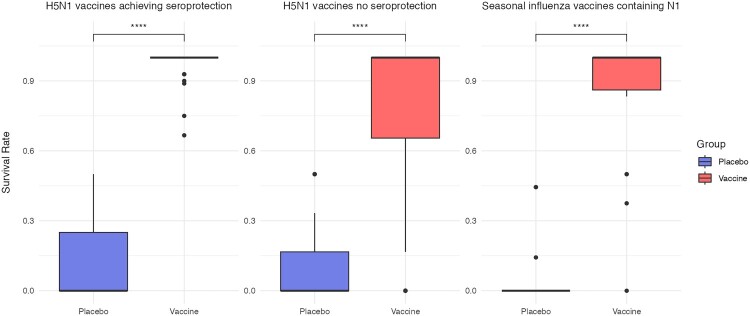
**Survival rates in vaccinated and placebo-treated ferrets.** (A) H5N1 vaccine trials achieving seroprotection, (B) H5N1 vaccine trials without seroprotection, and (C) N1-containing seasonal influenza vaccine trials. Boxes show interquartile ranges, horizontal lines indicate medians, whiskers represent ranges, and dots denote outliers. Statistical comparisons between vaccine and placebo groups were performed using Student’s *t*-test (**** *p* < 0.001). Vaccinated ferrets showed significantly higher survival in all categories.

Data for survival rate are shown in Figure 7. Vaccinated ferrets had median survival rates above 90% in seasonal influenza vaccine trials, H5N1 vaccine trials with seroprotection, and those without seroprotection, compared with 0% in the corresponding control groups (*p* < 0.001 for all comparisons). A summary of findings for VE of seasonal influenza vaccines and H5N1 vaccines (overall, with seroprotection, and without seroprotection) is presented in Supplementary Figure 19.

## Discussion

Human challenge trials are widely regarded as the gold standard for evaluating vaccine efficacy, but these are impossible since H5N1 infection in humans has been associated with a historic case fatality rate of 52% [28], markedly higher than those of SARS (10%), MERS (37%), and COVID-19 (0.007–10.1%, depending on age) [29]. Furthermore, current low incidence of H5N1 infection makes randomized clinical trials with clinical endpoints impossible. Pre-licensure human H5N1 vaccine trials have relied solely on immunogenicity endpoint. Given the ethical impossibility of conducting human H5N1 challenge studies, and the lack of feasibility for randomized clinical trials with clinical endpoints, meta-analyses of ferret challenge trials provide the best available evidence for assessing vaccine efficacy against H5N1. Ferret lung physiology closely mimics that of humans both for influenza pathogenesis and viral transmissibility.

To our knowledge, this is the first systematic review and meta-analysis of ferret challenge studies evaluating the efficacy of seasonal influenza vaccines against lethal H5N1 infection. Our findings show that seasonal influenza vaccines confer significant protection against H5N1-associated mortality in the ferret model. The VE against lethal H5N1 challenge was 73% (95% CI 54–84) for seasonal influenza vaccines containing the N1 antigen. Although this VE was lower than that of H5N1 vaccines achieving seroprotection (88%; *p* < 0.01), the difference in magnitude does not materially alter the overall interpretation that seasonal influenza vaccines provided substantial cross-protection against H5N1 mortality in ferrets.

Our findings indicate that N1-containing seasonal influenza vaccines conferred 73% efficacy against H5N1-associated mortality in ferrets, despite not achieving seroprotection by conventional HI or MN assays targeting the H5 antigen [20,30]. In contrast, seasonal influenza vaccines lacking N1 did not confer significant cross-protection. These observations are consistent with an earlier ex vivo immunology study [20] and a ferret challenge study [21], which together suggest that seasonal influenza vaccination may confer cross-protection against H5N1 through N1-directed cellular and antibody-dependent mechanisms in the absence of HA-matched immunity. This interpretation is further supported by experimental evidence in ferrets that prior H1N1 infection reduced replication and transmission of H5N1 challenge virus more effectively than prior H5N7 infection, further implicating N1-mediated immunity [31]. Taken together, these prior mechanistic studies provide a biological framework for our pooled findings and strengthen the plausibility that N1-directed immunity is a major contributor to the cross-protection observed here.

An unresolved question arising from the observed N1-directed cross-protection is the extent to which antigenic or genetic relatedness between the vaccine N1 and the challenge-virus N1 is required, particularly given the well-established strain-specific immune response patterns in ferrets. None of the included studies directly evaluated genetic or antigenic relatedness between the N1 neuraminidase of the seasonal H1N1 vaccine strain and that of the H5N1 challenge virus. Future studies addressing this question could clarify the degree of N1 matching necessary for cross-protection and help inform more rigorous characterization and quality control of the N1 component in seasonal influenza vaccine production.

We observed that seasonal influenza vaccines lacking N1 were not associated with statistically significant cross-protection; however, this estimate was based on only five trials and may therefore have been underpowered to detect smaller protective effects. Accordingly, these findings should not be interpreted as definitive evidence of no cross-protection. Additional immune mechanisms, such as vaccine-induced cell-mediated immunity or limited cross-reactive antibody responses not captured by standard neutralization assays, may also contribute. In humans, a prospective study reported that seasonal influenza vaccination increased H5N1 antibody titres in a subset of participants, with seroprotection achieved in 2.5–15.0% of non-elderly adults [18], suggesting that seasonal vaccines can elicit limited cross-reactive antibodies against H5 antigens. Similarly, seasonal influenza vaccination has been reported to induce cross-reactive antibodies recognizing the 2009 A(H1N1)pdm09 strain [32]. Together, these findings highlight the multifaceted nature of vaccine-induced cross-protection and underscore the need to clarify the underlying immunological mechanisms to inform the design of broader and more effective influenza vaccines against emerging H5N1 viruses.

Our results do not diminish the importance of H5N1 vaccines in the face of emerging H5N1 threats [8]. However, in the pre-pandemic setting, broad seasonal influenza vaccination coverage could represent a safe, widely available, and practical interim measure while strain-matched H5N1 vaccines are being developed and deployed. Current recommendations encouraging seasonal influenza vaccination aim primarily to prevent co-infection with H5N1 and seasonal influenza viruses, thereby reducing opportunities for reassortment and the emergence of novel strains with enhanced transmissibility or virulence. Beyond these indirect benefits, our findings in the ferret model raise the possibility that seasonal influenza vaccines may also provide some direct cross-protection against H5N1-associated mortality. However, whether such protection occurs in humans remains uncertain and requires confirmation in human observational and serological studies.

It is important to note that all trials included in our study were forced lethal-challenge experiments in immunologically naïve animals with mortality as the primary outcome. Therefore, these ferret-derived findings may not be directly generalizable to real-world prevention of H5N1 infection, transmission, or disease severity in humans, whose diverse histories of influenza infection and vaccination are likely to shape baseline immune memory. Nevertheless, supporting evidence from a previous study showed that H1N1-specific memory B cells induced by seasonal influenza vaccination cross-reacted with H5N1 in 45% of adult participants [33]. This finding suggests that immunity induced by seasonal influenza vaccination may facilitate a more rapid secondary immune response upon H5N1 exposure.

Although our study did not assess the effect of vaccination on viral load or transmission in the ferret model, emerging evidence suggests that seasonal influenza vaccination and prior influenza A(H1N1)pdm09 virus infection may offer some degree of protection against H5N1 viral replication, shedding, and possibly transmission in ferrets [21,31]. Even partial cross-reactive immunity could still have public health relevance if it reduces susceptibility or disease severity and thereby helps mitigate early spread, buying time for the development and deployment of strain-matched H5N1 vaccines.

Although our analysis included a clade 2.3.4.4b challenge strain and did not detect significant differences in vaccine effectiveness across H5N1 clades, the available data remain insufficient to establish whether the observed cross-protection extends to the rapidly evolving 2.3.4.4b variants currently circulating in cattle and other mammals.

An important limitation in the context of pandemic preparedness is that the apparent cross-protective effect identified in our study is most consistent with immunity directed against N1. Accordingly, these findings may be more relevant to emerging avian influenza viruses bearing N1 neuraminidase, such as H5N1, than to non-N1 viruses such as H5N8 [34]. If a future epidemic or pandemic were caused by a non-N1 avian influenza virus, the relevance of N1-directed cross-protection from seasonal influenza vaccination would be expected to be substantially lower.

Our study has several limitations. First, ferret studies did not use blinding or randomization protocols. However, the primary outcome – mortality – is unlikely to have been influenced by subjective bias. Moreover, unlike humans, ferrets used in experimental settings are confirmed influenza-negative before challenge, reducing inter-individual variability and limiting the need for strict randomization. Second, although unpublished full reports eligible for inclusion were not identified, some potentially relevant data may remain unavailable. This limitation should be considered when interpreting the findings. In particular, animal challenge studies are inherently susceptible to selective reporting. Because there is no registry for ferret experiments, we cannot completely exclude publication bias. Nevertheless, the observed cross-protection conferred by seasonal influenza vaccines is unlikely to be solely an artefact of publication bias, as five ferret trials reporting no cross-protection by seasonal influenza vaccines lacking the N1 antigen were included, and subgroup analysis by sample size – which detected possible publication bias for H5N1 vaccines without seroprotection – did not indicate such bias for N1-containing seasonal influenza vaccines. Third, multiple trials from the same publication were included as separate observations when they represented distinct experimental comparisons; however, residual non-independence within studies cannot be fully excluded. Fourth, under the 3R ethical principle, ferret trials reported within the same study often shared a common control group. However, this is not a methodological problem from biological viewpoint, as mortality rate in the control group is, by definition, not a random variable. The strain and inoculum dose of the challenge H5N1 virus determine the mortality rate in animal experiments. To assess VE against H5N1-related mortality, researchers must ensure that the challenge strain and dose are lethal. This is the purpose of using a control group in such experiments. The inherently low variance in mortality reduced the need for additional control animals to achieve precise VE estimation. Fifth, although VE did not differ significantly across platforms among N1-containing seasonal influenza vaccines but could still vary in magnitude, the diversity of vaccine platforms used in ferret studies complicates direct extrapolation to traditional non-adjuvanted trivalent inactivated seasonal influenza vaccines, which may be less immunogenic than newer platforms [21]. Thus, while current seasonal influenza vaccines generally contain an H1N1 component and therefore include N1, our findings should not be interpreted as indicating that all such vaccines would necessarily confer the same degree of protection against H5N1. Nonetheless, the landscape of commercial seasonal influenza vaccines is evolving, with cell culture-based, adjuvanted, recombinant, and live-attenuated formulations offering enhanced immunogenicity. An additional limitation is that some included trials used reassortant challenge viruses incorporating H5 on a PR8 (N1) backbone rather than fully wild-type H5N1 viruses. Because influenza pathogenicity and lethality can be influenced by internal gene constellations, mortality observed in these experiments may partly reflect properties of the PR8-derived internal genes rather than those of naturally circulating H5N1 viruses alone. This may have introduced heterogeneity into the pooled estimates. Moreover, differences in serological assays, including HI assays using different red blood cells and MN assays, may have reduced comparability of seroprotection assessments across H5N1 trials and thereby introduced bias into the pooled estimate. Finally, VE estimates from ferret trials, in which viral challenge was administered predominantly 14–28 days after vaccination, may not be directly generalizable to humans, because in pandemic preparedness scenarios the interval between vaccination and exposure to H5N1 would likely be much longer, potentially resulting in waning VE. Human investigations – such as observational cohort studies comparing vaccinated and unvaccinated high-risk individuals, and serological investigations – are needed to validate these findings and clarify their implications for human populations.

In conclusion, this systematic review and meta-analysis of available ferret H5N1 lethal-challenge studies indicates that seasonal influenza vaccines were associated with substantial protection against H5N1-associated mortality in this animal model. Although such cross-protection cannot replace the strain-specific immunity provided by H5N1 vaccines during a pandemic, it may represent a potential interim benefit in pre-pandemic preparedness. However, the relevance of these findings to humans remains uncertain. Human observational and serological studies are needed to determine whether the cross-protective effects observed in ferrets translate into clinically meaningful protection in human populations.

## Supplementary Material

ICMJE_Vermund.docx

Supplementary Appendix.pdf

ICMJE_WL Shih.docx

ICMJE_YC Huang.docx

ICMJE_IS Tesng.docx

ICMJE_CT Fang.docx

ICMJE_CH Chou.docx

## References

[1] Pulit-Penaloza JA, Brock N, Belser JA, et al. Highly pathogenic avian influenza A(H5N1) virus of clade 2.3.4.4b isolated from a human case in Chile causes fatal disease and transmits between co-housed ferrets. Emerg Microbes Infect. 2024;13(1):2332667. Epub 20240613. PubMed PMID: 38494746; PubMed Central PMCID: PMCPMC11177717. doi:10.1080/22221751.2024.2332667 38494746 PMC11177717

[2] Vreman S, Kik M, Germeraad E, et al. Zoonotic mutation of highly pathogenic avian influenza H5N1 virus identified in the brain of multiple wild carnivore species. Pathogens. 2023;12(2):168. Epub 2023 Feb 26. PubMed PMID: 36839440; PubMed Central PMCID: PMCPMC9961074. doi:10.3390/pathogens12020168 36839440 PMC9961074

[3] Burrough ER, Magstadt DR, Petersen B, et al. Highly pathogenic avian influenza A(H5N1) clade 2.3.4.4b virus infection in domestic dairy cattle and cats, United States, 2024. Emerg Infect Dis. 2024;30(7):1335–1343. Epub 2024 Apr 29. PubMed PMID: 38683888; PubMed Central PMCID: PMCPMC11210653. doi:10.3201/eid3007.240508 38683888 PMC11210653

[4] Centers for Disease Control and Prevention. Current H5N1 Bird Flu Situation in Dairy Cows [updated 2025 Jul 7] [cited 2025 Oct 18]. Available from: https://www.cdc.gov/bird-flu/situation-summary/mammals.html

[5] Uyeki TM, Milton S, Abdul Hamid C, et al. Highly pathogenic avian influenza A(H5N1) virus infection in a dairy farm worker. N Engl J Med. 2024;390(21):2028–2029. Epub 2024 May 3. PubMed PMID: 38700506. doi:10.1056/NEJMc2405371 38700506

[6] Leonard J, Harker EJ, Szablewski CM, et al. Notes from the field: seroprevalence of highly pathogenic avian influenza A(H5) virus infections among bovine veterinary practitioners – United States, September 2024. MMWR Morb Mortal Wkly Rep. 2025;74(4):50–52. Epub 20250213. PubMed PMID: 39946278; PubMed Central PMCID: PMCPMC11824947. doi:10.15585/mmwr.mm7404a2 39946278 PMC11824947

[7] World Health Organization. Cumulative number of confirmed human cases for avian influenza A(H5N1) reported to WHO, 2003-2025 [updated 25 2025 Aug] [cited 2025 Oct 18]. Available from: https://www.who.int/publications/m/item/cumulative-number-of-confirmed-human-cases-for-avian-influenza-a(h5n1)-reported-to-who–2003-2025–25-august-2025

[8] Bartlett ML, Palese P, Davis MF, et al. Enhancing the response to avian influenza in the US and globally. Lancet Reg Health Am. 2025;46:101100. Epub 20250428. PubMed PMID: 40625789; PubMed Central PMCID: PMCPMC12230410. doi:10.1016/j.lana.2025.101100 40625789 PMC12230410

[9] de Vries RD, Altenburg AF, Nieuwkoop NJ, et al. Induction of cross-clade antibody and T-cell responses by a modified vaccinia virus Ankara-based influenza A(H5N1) vaccine in a randomized phase 1/2a clinical trial. J Infect Dis. 2018;218(4):614–623. PubMed PMID: 29912453; PubMed Central PMCID: PMCPMC6047453. doi:10.1093/infdis/jiy214 29912453 PMC6047453

[10] Bresson JL, Perronne C, Launay O, et al. Safety and immunogenicity of an inactivated split-virion influenza A/Vietnam/1194/2004 (H5N1) vaccine: phase I randomised trial. Lancet. 2006;367(9523):1657–1664. PubMed PMID: 16714186. doi:10.1016/S0140-6736(06)68656-X 16714186

[11] Bernstein DI, Edwards KM, Dekker CL, et al. Effects of adjuvants on the safety and immunogenicity of an avian influenza H5N1 vaccine in adults. J Infect Dis. 2008;197(5):667–675. PubMed PMID: 18260764. doi:10.1086/527489 18260764

[12] Lin YJ, Shih YJ, Chen CH, et al. Aluminum salts as an adjuvant for pre-pandemic influenza vaccines: a meta-analysis. Sci Rep. 2018;8(1):11460. Epub 20180730. PubMed PMID: 30061656; PubMed Central PMCID: PMCPMC6065440. doi:10.1038/s41598-018-29858-w 30061656 PMC6065440

[13] Finnish Institute for Health and Welfare. Avian influenza vaccinations begin – vaccine to be offered to persons at increased risk of infection [updated 2024 Jun 25] [cited 2025 Oct 18]. Available from: https://thl.fi/en/-/avian-influenza-vaccinations-begin-vaccine-to-be-offered-to-persons-at-increased-risk-of-infection

[14] Centers for Disease Control and Prevention. Highly pathogenic avian influenza A(H5N1) virus in animals: interim recommendations for prevention, monitoring, and public health investigations [updated 2024 Nov 8] [cited 2025 Oct 18]. Available from: https://www.cdc.gov/bird-flu/prevention/hpai-interim-recommendations.html?CDC_AAref_Val=https://www.cdc.gov/flu/avianflu/hpai/hpai-interim-recommendations.html

[15] Furey C, Scher G, Ye N, et al. Development of a nucleoside-modified mRNA vaccine against clade 2.3.4.4b H5 highly pathogenic avian influenza virus. Nat Commun. 2024;15(1):4350. Epub 20240523. PubMed PMID: 38782954; PubMed Central PMCID: PMCPMC11116520. doi:10.1038/s41467-024-48555-z 38782954 PMC11116520

[16] United States Department of Health and Human Services. HHS winds down mRNA vaccine development under BARDA [updated 2025 Aug 5] [cited 2025 Oct 18]. Available from: https://www.hhs.gov/press-room/hhs-winds-down-mrna-development-under-barda.html

[17] Roos A, Roozendaal R, Theeuwsen J, et al. Protection against H5N1 by multiple immunizations with seasonal influenza vaccine in mice is correlated with H5 cross-reactive antibodies. Vaccine. 2015;33(14):1739–1747. Epub 20150207. PubMed PMID: 25659276. doi:10.1016/j.vaccine.2015.01.070 25659276

[18] Sanz-Muñoz I, Sánchez-Martínez J, Rodríguez-Crespo C, et al. Are we serologically prepared against an avian influenza pandemic and could seasonal flu vaccines help us? mBio. 2024;16(2):e0372124. Epub 20241231. PubMed PMID: 39745389. doi:10.1128/mbio.03721-24 39745389 PMC11796349

[19] Bethell D, Saunders D, Jongkaewwattana A, et al. Evaluation of in vitro cross-reactivity to avian H5N1 and pandemic H1N1 2009 influenza following prime boost regimens of seasonal influenza vaccination in healthy human subjects: a randomised trial. PLoS One. 2013;8(3):e59674. Epub 20130326. PubMed PMID: 23555741; PubMed Central PMCID: PMCPMC3608534. doi:10.1371/journal.pone.0059674 23555741 PMC3608534

[20] Gioia C, Castilletti C, Tempestilli M, et al. Cross-subtype immunity against avian influenza in persons recently vaccinated for influenza. Emerg Infect Dis. 2008;14(1):121–128. PubMed PMID: 18258091; PubMed Central PMCID: PMCPMC2600140. doi:10.3201/eid1401.061283 18258091 PMC2600140

[21] Sun X, Subbiah J, Belser JA, et al. Effect of seasonal influenza vaccines on avian influenza A(H5N1) clade 2.3.4.4b virus infection in ferrets. Emerg Infect Dis. 2025;31(10):1950–1960. Epub 2025 Sep 29. PubMed PMID: 41017046; PubMed Central PMCID: PMCPMC12483109. doi:10.3201/eid3110.250668 41017046 PMC12483109

[22] Keitel WA, Voronca DC, Atmar RL, et al. Effect of recent seasonal influenza vaccination on serum antibody responses to candidate pandemic influenza A/H5N1 vaccines: A meta-analysis. Vaccine. 2019;37(37):5535–5543. Epub 20190531. PubMed PMID: 31160101. doi:10.1016/j.vaccine.2019.04.066 31160101

[23] Shetty N, Shephard MJ, Rockey NC, et al. Influenza virus infection and aerosol shedding kinetics in a controlled human infection model. J Virol. 2024;98(12):e0161224. Epub 2024 Nov 26. PubMed PMID: 39589151; PubMed Central PMCID: PMCPMC11657674. doi:10.1128/jvi.01612-24 39589151 PMC11657674

[24] Lai J, Sobhani H, Coleman KK, et al. Evaluating modes of influenza transmission (EMIT-2): insights from lack of transmission in a controlled transmission trial with naturally infected donors. PLoS Pathog. 2026;22(1):e1013153. Epub 2026 Jan 7. PubMed PMID: 41499634; PubMed Central PMCID: PMCPMC12799188. doi:10.1371/journal.ppat.1013153 41499634 PMC12799188

[25] Belser JA, Katz JM, Tumpey TM. The ferret as a model organism to study influenza A virus infection. Dis Model Mech. 2011;4(5):575–579. Epub 20110802. PubMed PMID: 21810904; PubMed Central PMCID: PMCPMC3180220. doi:10.1242/dmm.007823 21810904 PMC3180220

[26] Macleod MR, O’Collins T, Howells DW, et al. Pooling of animal experimental data reveals influence of study design and publication bias. Stroke. 2004;35(5):1203–1208. Epub 20040401. PubMed PMID: 15060322. doi:10.1161/01.STR.0000125719.25853.20 15060322

[27] Schunemann HJ, Brennan S, Akl EA, et al. The development methods of official GRADE articles and requirements for claiming the use of GRADE – a statement by the GRADE guidance group. J Clin Epidemiol. 2023;159:79–84. Epub 20230519. PubMed PMID: 37211327. doi:10.1016/j.jclinepi.2023.05.010 37211327

[28] Adlhoch C, Fusaro A, Gonzales JL, et al. Avian influenza overview April–June 2023. EFSA J. 2023;21(7):e08191. Epub 20230720. PubMed PMID: 37485254; PubMed Central PMCID: PMCPMC10358191. doi:10.2903/j.efsa.2023.8191 37485254 PMC10358191

[29] Granata G, Simonsen L, Petrosillo N, et al. Mortality of H5N1 human infections might be due to H5N1 virus pneumonia and could decrease by switching receptor. Lancet Infect Dis. 2024;24(9):e544–e545. doi:10.1016/S1473-3099(24)00460-2 39067462

[30] Tsai C, Caillet C, Hu H, et al. Measurement of neutralizing antibody responses against H5N1 clades in immunized mice and ferrets using pseudotypes expressing influenza hemagglutinin and neuraminidase. Vaccine. 2009;27(48):6777–6790. Epub 20090902. PubMed PMID: 19732860; PubMed Central PMCID: PMCPMC7115403. doi:10.1016/j.vaccine.2009.08.056 19732860 PMC7115403

[31] Sun X, Belser JA, Li ZN, et al. Effect of prior influenza A(H1N1)pdm09 virus infection on pathogenesis and transmission of human influenza A(H5N1) clade 2.3.4.4b virus in ferret model. Emerg Infect Dis. 2025;31(3):458–466. Epub 2025 Mar 3. PubMed PMID: 40023783; PubMed Central PMCID: PMCPMC11878296. doi:10.3201/eid3103.241489 40023783 PMC11878296

[32] Huang DT, Shao PL, Huang KC, et al. Serologic status for pandemic (H1N1) 2009 virus, Taiwan. Emerg Infect Dis. 2011;17(1):76–78. PubMed PMID: 21192858; PubMed Central PMCID: PMCPMC3204618. doi:10.3201/eid1701.100014 21192858 PMC3204618

[33] Buricchi F, Bardelli M, Malzone C, et al. Impact of preexisting memory to seasonal A/H1N1 influenza virus on the immune response following vaccination against avian A/H5N1 virus. Eur J Immunol. 2013;43(3):641–648. Epub 20130124. PubMed PMID: 23238926. doi:10.1002/eji.201242563 23238926

[34] Zeng J, Du F, Xiao L, et al. Spatiotemporal genotype replacement of H5N8 avian influenza viruses contributed to H5N1 emergence in 2021/2022 panzootic. J Virol. 2024;98(3):e0140123. Epub 2024 Feb 15. PubMed PMID: 38358287; PubMed Central PMCID: PMCPMC10949427. doi:10.1128/jvi.01401-23 38358287 PMC10949427

