# Supplementary material for: Cross-protection against highly pathogenic avian influenza H5N1 virus from seasonal influenza vaccines: a systematic review and meta-analysis of ferret studies
Source: Emerg Microbes Infect. 2026 Apr 15;15(1):2654278. doi: 10.1080/22221751.2026.2654278 (PMC13084842; doi:10.1080/22221751.2026.2654278)
Supplement: ICMJE_Vermund.docx [file TEMI_A_2654278_SM0936.docx]

| ICMJE DISCLOSURE FORM | |
| --- | --- |
| **Date:** | 12/10/2025 |
| **Your Name:** | Sten H. Vermund |
| **Manuscript Title:** | Cross-protection against highly pathogenic avian influenza H5N1 from seasonal influenza vaccination: a systematic review and meta-analysis of mortality in ferret studies |
| **Manuscript Number (if known):** | Not yet known |
| In the interest of transparency, we ask you to disclose all relationships/activities/interests listed below that are related to the content of your manuscript. “Related” means any relation with for-profit or not-for-profit third parties whose interests may be affected by the content of the manuscript. Disclosure represents a commitment to transparency and does not necessarily indicate a bias. If you are in doubt about whether to list a relationship/activity/interest, it is preferable that you do so.  The author’s relationships/activities/interests should be defined broadly. For example, if your manuscript pertains to the epidemiology of hypertension, you should declare all relationships with manufacturers of antihypertensive medication, even if that medication is not mentioned in the manuscript.  In item #1 below, report all support for the work reported in this manuscript without time limit. For all other items, the time frame for disclosure is the past 36 months. | |

|  | | | **Name all entities with whom you have this relationship or indicate none (add rows as needed)** | **Specifications/Comments (e.g., if payments were made to you or to your institution)** |
| --- | --- | --- | --- | --- |
| **Time frame: Since the initial planning of the work** | | | | |
| **1** | All support for the present manuscript (e.g., funding, provision of study materials, medical writing, article processing charges, etc.)  **No time limit for this item.** | | \|  \| **None** \| \| --- \| --- \|  \| Capacity building for l'Institut National de Santé Publique du Tchad; Mother and Child Health  supported by the U.S. Agency for International Development (USAID) and the President’s Emergency Plan for AIDS Relief (PEPFAR) through the Meeting Targets and Maintaining Epidemic Control (EpiC) project to prime awardee FHI 360 through subaward ID number PO23000096 to the Yale School of Public Health (YSPH). \| Payment to Yale School of Public Health with subcontract to the University of N’Djamena from USAID funding to FHI 360. \| \| --- \| --- \| \| Initial funding was also from USAID via a Human Resources for Health (HRH) 2030 award to Chemonics Inc. with a subcontract to YSPH. \| Payment to Yale School of Public Health with subcontract to the University of N’Djamena from USAID funding to Chemonics, Inc. \| | |
| **Time frame: past 36 months** | | | | |
| **2** | | Grants or contracts from any entity (if not indicated in item #1 above). | \|  \| **None** \| \| --- \| --- \|  \| Center for Interdisciplinary Research on AIDS \| NIH grant P30MH062294-co-investigator (co-I), ended 1/1/2025 \| \| --- \| --- \| \| Prescription Medications and Motor Vehicle Crashes \| Grant from the Insurance Institute for Highway Safety-Principal Investigator (PI), ended 1/1/2025 \| \| Developing causal inference methods to evaluate and leverage spillover effects through social Interactions for designing improved HIV prevention interventions. \| NIH grant R01MH134715 – co-I, ended 1/1/2025 \| \| Molecular Virology/Epidemiology HIV Training in Kazakhstan (MoVE-Kaz) \| NIH grant D43TW012507 (PI) \| \| Consultative support for the CT Dept. of Education for COVID-19 risk reduction \| State of CT Dept of Education Project: 23SDE0099AA/22SDE0097AA (PI): now turned over to a Yale faculty member as of 1/1/2025 \| \| HIV Prevention Trials Network (HPTN) Leadership Group \| NIH grant UM1AI068619 (SHV is PI, USF sub-contract from FHI 360) \| \| Capacity building for l'Institut National de Santé Publique du Tchad; Mother and Child Health supported by the U.S. Agency for International Development (USAID) and the President’s Emergency Plan for AIDS Relief (PEPFAR) through the Meeting Targets and Maintaining Epidemic Control (EpiC) project to prime awardee FHI 360 through subaward ID number PO23000096 to the Yale School of Public Health (YSPH). \| Payment to Yale School of Public Health with subcontract to the University of N’Djamena from USAID funding to FHI 360. Terminated in June 2025 with the closure of USAID. \| | |
| **3** | | Royalties or licenses | \|  \| **None** \| \| --- \| --- \|  \| Humana Press, book royalties in theory, but none to date. \| Humphries DL, Scott ME, Vermund SH, eds. *Nutrition and Infectious Disease: Shifting the Clinical Paradigm.* Cham, Switzerland: Humana Press/Springer Nature Switzerland AG, 2021: vii-xvii, ISBN 978-3-030-56913-6. \| \| --- \| --- \| \|  \|  \| \|  \|  \| | |
| **4** | | Consulting fees | \|  \| **None** \| \| --- \| --- \|  \|  \|  \| \| --- \| --- \| \|  \|  \| | |
| **5** | | Payment or honoraria for lectures, presentations, speakers bureaus, manuscript writing or educational events | \|  \| **None** \| \| --- \| --- \|  \| Moderna, Inc. \| Training for staff in Implementation Science and Teaching for clinicians on SARS-CoV-2 and vaccines \| \| --- \| --- \| \|  \|  \| \|  \|  \| | |
| **6** | | Payment for expert testimony | \|  \| **None** \| \| --- \| --- \|  \|  \|  \| \| --- \| --- \| \|  \|  \| \|  \|  \| | |
| **7** | | Support for attending meetings and/or travel | \|  \| **None** \| \| --- \| --- \|  \| Moderna, Inc. \| See section 5 \| \| --- \| --- \| | |
| **8** | | Patents planned, issued or pending | \|  \| **None** \| \| --- \| --- \|  \|  \|  \| \| --- \| --- \| \|  \|  \| \|  \|  \| | |
| **9** | | Participation on a Data Safety Monitoring Board or Advisory Board | \|  \| **None** \| \| --- \| --- \|  \| Moderna, Inc. \| Scientific Advisory Board (vaccine hesitancy) \| \| --- \| --- \| \| Tevogen Bio, Inc. \| Scientific Advisory Board (no marketed products) \| \| Porosome Therapeutics, Inc. \| Scientific Advisory Board (no marketed products) \| \| Immugen BioPharma, Inc. \| Scientific Advisory Board (no marketed products) \| | |
| **10** | | Leadership or fiduciary role in other board, society, committee or advocacy group, paid or unpaid | \|  \| **None** \| \| --- \| --- \|  \| Global Virus Network, Inc. \| Chief Medical Officer \| \| --- \| --- \| \| Connecticut Academy of Science and Engineering \| President to 12/31/2024 \| \|  \|  \| | |
| **11** | | Stock or stock options | \|  \| **None** \| \| --- \| --- \|  \| Tevogen Bio, Inc. \| For Scientific Advisory Board service \| \| --- \| --- \| \| Porosome Therapeutics, Inc. \| For Scientific Advisory Board service \| \|  \|  \| | |
| **12** | | Receipt of equipment, materials, drugs, medical writing, gifts or other services | \|  \| **None** \| \| --- \| --- \|  \|  \|  \| \| --- \| --- \| \|  \|  \| \|  \|  \| | |
| **13** | | Other financial or non-financial interests | \|  \| **None** \| \| --- \| --- \|  \|  \|  \| \| --- \| --- \| \|  \|  \| \|  \|  \| | |
|  | |  |  | |
| **Please place an “X” next to the following statement to indicate your agreement:** | | | | |
|  | | I certify that I have answered every question and have not altered the wording of any of the questions on this form. | | |
