# Supplementary material for: Cross-protection against highly pathogenic avian influenza H5N1 virus from seasonal influenza vaccines: a systematic review and meta-analysis of ferret studies
Source: Emerg Microbes Infect. 2026 Apr 15;15(1):2654278. doi: 10.1080/22221751.2026.2654278 (PMC13084842; doi:10.1080/22221751.2026.2654278)
Supplement: Supplementary Appendix.pdf [file TEMI_A_2654278_SM0935.pdf]

Supplementary Appendix

Supplement to:

Tseng et al. Cross-protection against highly pathogenic avian influenza H5N1 virus from seasonal influenza vaccines: a systematic review and meta-analysis of ferret studies

Supplementary Results

1. Seroprotection: seasonal influenza vaccines
2. Seroprotection: H5N1 vaccines
3. Effect of H5N1 boosters

Supplementary Tables

Supplementary Table 1. The query string during literature search in PubMed, Embase, and Science Citation Index (Web of Science) databases ranging from January 1, 1997, to July 5, 2025.

Supplementary Table 2. List of excluded studies and the reasons for exclusion.

Supplementary Table 3. Study characteristics of trials evaluating the vaccine efficacy of seasonal influenza and H5N1 vaccines in ferrets. One published study may report multiple trials, which were labeled in the order described in the article with numbers in parentheses.

(a) Publication year, vaccine type, vaccine and challenging strain, description, and vaccination regimen.

(b) Numbers of animals in control and vaccinated groups, numbers of events in each group, study design, serological assay method, post-vaccination antibody titers, and seroprotection status.

Note: "Survival rate" refers to the proportion of vaccinated ferrets that survived the lethal challenge; "Time Interval" refers to the time interval from vaccination to challenge (day); "Test Virus" denotes the virus strain used to measure seroprotection; "Seroprotection" indicates whether the vaccinated group achieved seroprotective antibody levels before challenge. HI = hemagglutination inhibition assay; MN = microneutralization assay; MDCK = Madin–Darby canine kidney cell; NA = not available.

Supplementary Table 4. PRISMA checklist.

Supplementary Figures

Supplementary Figure 1. Vaccine efficacy in all H5N1 vaccine trials versus N1-containing seasonal influenza vaccine trials.

Supplementary Figure 2. Vaccine efficacy in H5N1 vaccine trials with seroprotection versus H5N1 vaccine trials without seroprotection.

Supplementary Figure 3. Vaccine efficacy in H5N1 vaccine trials with seroprotection: subgroup analysis by booster dose.

Supplementary Figure 4. Vaccine efficacy in H5N1 vaccine trials with seroprotection versus N1-containing seasonal influenza vaccine trials.

Supplementary Figure 5 Vaccine efficacy in H5N1 vaccine trials without seroprotection versus N1-containing seasonal influenza vaccine trials.

Supplementary Figure 6. Vaccine efficacy in all vaccines: subgroup analysis by the clade of the challenge virus.

Supplementary Figure 7. Vaccine efficacy of H5N1 vaccines: subgroup analysis by the time interval from vaccination to challenge.

Supplementary Figure 8. Vaccine efficacy of N1-containing seasonal vaccines: subgroup analysis by the time interval from vaccination to challenge.

Supplementary Figure 9. Vaccine efficacy in N1-containing seasonal vaccines: subgroup analysis by vaccine platform.

Supplementary Figure 10. Vaccine efficacy in N1-containing seasonal vaccines: subgroup analysis by adding adjuvant or not.

Supplementary Figure 11. Vaccine efficacy in N1-containing seasonal vaccine: subgroup analysis by booster or not.

Supplementary Figure 12. Vaccine efficacy in N1-containing seasonal influenza vaccine: subgroup analysis by the study year.

Supplementary Figure 13. Leave-one-out forest plot for N1-containing seasonal influenza vaccine trials.

Supplementary Figure 14. Leave-one-out forest plot for H5N1 vaccines trials achieving seroprotection.

Supplementary Figure 15. Quality assessment of each study according to Collaborative Approach to Meta-analysis and Review of Animal Data in Experimental Studies (CAMARADES) checklist.

Supplementary Figure 16. Summary of quality assessment.

Supplementary Figure 17. Publication bias assessment of H5N1 influenza vaccine trials with seroprotection by sample size category.

Supplementary Figure 18. Publication bias assessment of H5N1 influenza vaccine trials without seroprotection by sample size category.

Supplementary Figure 19. Summary of findings.

Supplementary Results

Seroprotection: seasonal influenza vaccines

Among the 24 trials assessing seasonal influenza vaccines, hemagglutination inhibition (HI) assays against the challenge strain were conducted in 22 (14 using chicken red blood cells, three using horse red blood cells, two using turkey red blood cells, and three not specifying the source of red blood cells). The remaining two trials did not report post-vaccination titers and were assumed not to have achieved H5N1 seroprotection. None of the seasonal influenza vaccine trials achieved H5N1 seroprotection. Only one trial, Haynes et al. (2009), reported a borderline HI titer of 1:29 using horse red blood cells.

Seroprotection: H5N1 vaccines

Among the 133 trials assessing H5N1 vaccines, HI assays were performed in 123 (75 using horse red blood cells, 38 using chicken red blood cells, six using human red blood cells, and four not specifying the source of red blood cells). Microneutralization (MN) assays were used in the remaining 10 trials. Overall, 66 (50%) of the 133 H5N1 vaccine trials achieved seroprotection. The likelihood of achieving seroprotection did not differ by neutralization assay method (HI 49% vs MN 60%; Fisher's exact test p=0.53).

The test strain used for assessing seroprotection was the challenge strain in 106 trials (identical to the vaccine strain in 68 and different in 38), the vaccine strain (different from the challenge strain) in 22 trials, and a heterologous strain (different from both the vaccine and challenge strains) in five. The likelihood of achieving seroprotection did not differ by type of test strain (challenge strain 49% vs vaccine strain 55% vs heterologous 40%; Fisher's exact test p=0.82).

Among the 123 trials using HI assays, seven reported borderline HI titers between 1:20 and 1:39—three using chicken red blood cells, two using horse red blood cells, and two using human red blood cells. (Although HI assays using horse red blood cells tend to yield higher titers, the small number of trials (eight in total) reporting borderline titers makes it unlikely that the assay type affected the subgroup analysis results stratified by seroprotection.)

Effect of H5N1 boosters

To investigate the effect of H5N1 boosters on seroprotection, we stratified H5N1 vaccine trials by booster status. Seroprotection was achieved in 55.1% (65/118) of booster trials compared with 6.7% (1/15) of priming-only trials (p = 0.002; odds ratio [OR] 8.47, 95% CI 1.8–80.0). The use of a second booster was more likely to confer seroprotection than a single booster (93.3% [14/15] vs 49.5% [48/97]; p = 0.001; OR 14.0, 95% CI 2.0–614.5). We further examined whether the number of boosters affected vaccine efficacy. In an analysis restricted to H5N1 vaccines that achieved seroprotection (Supplementary Figure 3), pooled vaccine efficacy was 89% (95% CI 84–92; I^2=0%) for trials using a single booster, 85% (95% CI 73–92; I^2=0%) for trials using a second booster, and 89% (95% CI 29–98; I^2=0%) for trials using a third booster. No significant difference was

observed across subgroups using different numbers of boosters (p=0.79; Supplementary Figure 5), indicating that once seroprotection was achieved, additional boosters did not further improve vaccine efficacy.

Supplementary Table 1. The query string during literature search in PubMed, Embase, and Science Citation Index (Web of Science) databases ranging from January 1, 1997, to July 5, 2025.

String	Terms	PubMed	Embase	Web of Science
1	(avian influenza[Title]) OR (bird flu[Title]) OR (HPAI[Title]) OR (H5N1[Title]) OR (H5Nx[Title]) OR (influenza A[Title])	24,519	28,099	38,808
2	(efficacy[Title]) OR (protect[Title]) OR (protection[Title]) OR (immunization[Title]) OR (prevent[Title])	342,836	547,442	713,563
3	(vaccine[Title]) OR (vaccination[Title]) OR (hemagglutinin[Title])	157,851	202,265	219,298
4	1 AND 2 AND 3	454	504	1,315

Supplementary Table 2. List of excluded studies and the reasons for exclusion.

Note: DOI: Digital Object Identifier; NA = not available.

Study	Reason for exclusion	DOI
Donnelly, 1997	Challenging strains not H5N1	10.1016/s0264-410x(96)00268-x.
Palker, 2004	Challenging strains not H5N1	10.1016/j.virusres.2004.05.009.
Mann, 2006	Challenging strains not H5N1	10.1016/j.vaccine.2009.03.040.
Joseph, 2008	Challenging strains not H5N1	10.1016/j.virol.2008.05.021.
Mett, 2008	Challenging strains not H5N1	10.1016/j.vaccine.2007.01.017.
Cox, 2009	Challenging strains not H5N1	10.1111/j.1750-2659.2009.00082.x.
Del Giudice, 2009	Challenging strains not H5N1	10.1126/scitranslmed.3000564.
Shin, 2010	Challenging strains not H5N1	10.1089/vim.2010.0022.
Pearce, 2011	Challenging strains not H5N1	10.1089/vim.2010.0022
Hamouda, 2011	Challenging strains not H5N1	10.1128/CVI.00035-11
Jones, 2011	Challenging strains not H5N1	10.1016/j.vaccine.2011.07.073.
Petsch, 2012	Challenging strains not H5N1	10.1128/JVI.02625-12.
Houser, 2013	Challenging strains not H5N1	10.1128/JVI.02625-12.
Houser, 2013	Challenging strains not H5N1	10.1128/JVI.02434-13.
Ann, 2014	Challenging strains not H5N1	10.1016/j.vaccine.2014.08.029
Wong, 2014	Challenging strains not H5N1	10.1016/j.vaccine.2014.06.016.
Chen, 2014	Challenging strains not H5N1	10.1093/infdis/jit414.
Sun, 2014	Challenging strains not H5N1	1 10.1016/j.virol.2014.07.004.
Kreijtz, 2015	Challenging strains not H5N1	10.1093/infdis/jiu528.
Shen, 2016	Challenging strains not H5N1	10.1016/j.vaccine.2016.09.059
Broadbent, 2016	Challenging strains not H5N1	10.1016/j.vaccine.2015.11.054
Nachbagauer, 2017	Challenging strains not H5N1	10.1038/s41541-017-0026-4.
Wang, 2017	Challenging strains not H5N1	10.1128/JVI.01512-16.
Sun, 2017	Challenging strains not H5N1	10.1016/j.virol.2017.05.010
Paules, 2017	Challenging strains not H5N1	10.1093/infdis/jix292.
Nachbagauer, 2018	Challenging strains not H5N1	10.1172/JCI122895.
Yang, 2018	Challenging strains not H5N1	10.1038/s41426-018-0154-6
Holzer, 2018	Challenging strains not H5N1	10.4049/jimmunol.1800142.
Korenkov, 2018	Challenging strains not H5N1	10.1016/j.meegid.2018.06.019
Han, 2019	Challenging strains not H5N1	10.1007/s12275-019-8504-1.
Liu, 2019	Challenging strains not H5N1	10.3389/fimmu.2019.00756.
Isakova-Sivak, 2019	Challenging strains not H5N1	10.3390/vaccines7030061.
Sia, 2021	Challenging strains not H5N1	10.1073/pnas.2025759118.

Vidaña, 2021	Challenging strains not H5N1	10.1111/irv.12784.
Guilfoyle, 2021	Challenging strains not H5N1	10.1016/j.vaccine.2020.09.062.
Mezhenskaya, 2021	Challenging strains not H5N1	10.3390/v13071280.
van Diemen, 2023	Challenging strains not H5N1	10.3201/eid2909.230066.
Hill-Batorski, 2023	Challenging strains not H5N1	10.3390/vaccines11040798.
Ko, 2024	Challenging strains not H5N1	10.1038/s41467-024-54620-4.
Kishida, 2024	Challenging strains not H5N1	10.1111/1348-0421.13179.
Kannan, 2024	Challenging strains not H5N1	10.3390/vaccines12070724.
Wang, 2025	Challenging strains not H5N1	10.1038/s44298-025-00154-5.
Shi, 2025	Challenging strains not H5N1	10.1371/journal.pone.0308680.
Smith, 2012	Challenging strains not H5N1	10.1016/j.vaccine.2011.10.092.
Layton, 2011	Full-text not available	NA
Piras, 2011	Full-text not available	NA
Liu, 2014	Full-text not available	NA
Brichacek, 2016	Full-text not available	NA
Nurpeisova, 2019	Missing survival data	10.1007/s00705-019-04147-7.
Suguitan, 2009	Missing survival data	10.1016/j.virol.2009.09.017.
Giles, 2012	Missing survival data	10.1128/CVI.05533-11.
Baz, 2015	Missing survival data	10.1128/mBio.01487-15
Nuñez, 2021	No challenging test performed	10.3390/pathogens10111352.
Moin, 2022	No challenging test performed	10.1016/j.immuni.2022.10.015.
Suguitan, 2006	No lethal challenge	10.1371/journal.pmed.0030360.
Bodewes, 2010	No lethal challenge	10.1128/JVI.00549-10.
Siegers, 2016	No lethal challenge	10.1093/infdis/jiw123.
Song, 2012	No lethal challenge	10.1007/s12275-012-1573-z.
Cox, 2015	No lethal challenge	10.1371/journal.pone.0135723.

Supplementary Table 3. Study characteristics of trials evaluating the vaccine efficacy of seasonal influenza and H5N1 vaccines in ferrets: One published study may report multiple trials, which were labeled in the order described in the article with numbers in parentheses.

(a) Trial, publication year, vaccine type, vaccine and challenging strain, description, and vaccination regimen.

Trial	Year	Vaccine	Challenging strain	Vaccine type	Vaccine strain	Description	Booster
Hoffmann (1)	2005	H5N1 vaccine	A/Vietnam/1203/2004 (H5N1)	recombinant vaccine	A/HK/213/03 (H5N1) and A/Vietnam/1203/2004 (H5N1)	H5N1/03	1
Hoffmann (2)	2005	H5N1 vaccine	A/Vietnam/1203/2004 (H5N1)	recombinant vaccine	A/HK/213/03 (H5N1) and A/Vietnam/1203/2004 (H5N1)	H5/04	1
Hoffmann (3)	2005	H5N1 vaccine	A/Vietnam/1203/2004 (H5N1)	recombinant vaccine	A/HK/213/03 (H5N1) and A/Vietnam/1203/2004 (H5N1)	H5S2233N/04	1
Govorkova (1)	2006	H5N1 vaccine	A/Vietnam/1203/2004 (H5N1)	recombinant vaccine	A/Hong Kong/213/2003 (H5N1)	7 μg, 1 dose	0
Govorkova (2)	2006	H5N1 vaccine	A/Vietnam/1203/2004 (H5N1)	recombinant vaccine	A/Hong Kong/213/2003 (H5N1)	7 μg, 2 doses	1
Govorkova (3)	2006	H5N1 vaccine	A/Vietnam/1203/2004 (H5N1)	recombinant vaccine	A/Hong Kong/213/2003 (H5N1)	15 μg, 1 dose	0
Baras (1)	2008	H5N1 vaccine	A/Indonesia/05/05 (H5N1)	split-virion vaccine	A/H5N1/Vietnam/1194/04 (H5N1)	Unadjuvanted H5N1 (15 μg)	1
Baras (2)	2008	H5N1 vaccine	A/Indonesia/05/05 (H5N1)	split-virion vaccine	A/H5N1/Vietnam/1194/04 (H5N1)	Adjuvanted H5N1 (1.7 μg)	1
Baras (3)	2008	H5N1 vaccine	A/Indonesia/05/05 (H5N1)	split-virion vaccine	A/H5N1/Vietnam/1194/04 (H5N1)	Adjuvanted H5N1 (3.8 μg)	1
Baras (4)	2008	H5N1 vaccine	A/Indonesia/05/05 (H5N1)	split-virion vaccine	A/H5N1/Vietnam/1194/04 (H5N1)	Adjuvanted H5N1 (7.5 μg)	1
Baras (5)	2008	H5N1 vaccine	A/Indonesia/05/05 (H5N1)	split-virion vaccine	A/H5N1/Vietnam/1194/04 (H5N1)	Adjuvanted H5N1 (15 μg)	1

Lalor (1)	2008	H5N1 vaccine	A/Vietnam/1203/04 (H5N1)	DNA vaccine	A/Vietnam/1203/04 (H5N1)	NP+M2	1
Lalor (2)	2008	H5N1 vaccine	A/Vietnam/1203/04 (H5N1)	DNA vaccine	A/Vietnam/1203/04 (H5N1)	H5+NPA+M2A	1
Lalor (3)	2008	H5N1 vaccine	A/Vietnam/1203/04 (H5N1)	DNA vaccine	A/Vietnam/1203/04 (H5N1)	H5+NP+M2 (2 doses)	1
Lalor (4)	2008	H5N1 vaccine	A/Vietnam/1203/04 (H5N1)	DNA vaccine	A/Vietnam/1203/04 (H5N1)	H5+NP+M2 (1 dose)	0
Mahmood (1)	2008	H5N1 vaccine	A/Indonesia/05/2005 (H5N1)	recombinant subunit vaccine	A/Vietnam/1203/2004 (H5N1)	rHA clade 1	1
Mahmood (2)	2008	H5N1 vaccine	A/Indonesia/05/2005 (H5N1)	recombinant subunit vaccine	A/Indonesia/05/2005 (H5N1)	rHA clade 2.1	1
Mahmood (3)	2008	H5N1 vaccine	A/Indonesia/05/2005 (H5N1)	virus-like particles (VLPs)	A/Indonesia/05/2005 (H5N1)	VLP 0.6 µg	1
Mahmood (4)	2008	H5N1 vaccine	A/Indonesia/05/2005 (H5N1)	virus-like particles (VLPs)	A/Indonesia/05/2005 (H5N1)	VLP 3 µg	1
Mahmood (5)	2008	H5N1 vaccine	A/Indonesia/05/2005 (H5N1)	virus-like particles (VLPs)	A/Indonesia/05/2005 (H5N1)	VLP 15 µg	1
Mahmood (6)	2008	H5N1 vaccine	A/Vietnam/1203/2004 (H5N1)	recombinant subunit vaccine	A/Vietnam/1203/2004 (H5N1)	rHA clade 1	1
Mahmood (7)	2008	H5N1 vaccine	A/Vietnam/1203/2004 (H5N1)	recombinant subunit vaccine	A/Indonesia/05/2005 (H5N1)	rHA clade 2.1	1
Mahmood (8)	2008	H5N1 vaccine	A/Vietnam/1203/2004 (H5N1)	virus-like particles (VLPs)	A/Indonesia/05/2005 (H5N1)	VLP 0.6 µg	1
Mahmood (9)	2008	H5N1 vaccine	A/Vietnam/1203/2004 (H5N1)	virus-like particles (VLPs)	A/Indonesia/05/2005 (H5N1)	VLP 3 µg	1
Mahmood (10)	2008	H5N1 vaccine	A/Vietnam/1203/2004 (H5N1)	virus-like particles (VLPs)	A/Indonesia/05/2005 (H5N1)	VLP 15 µg	1

Forrest (1)	2009	H5N1 vaccine	A/Vietnam/1203/04 (H5N1)	recombinant vaccine	rg-A/Vietnam/1203/04	rg-A/Vietnam/1203/04 + adjuvant	1
Forrest (2)	2009	H5N1 vaccine	A/Vietnam/1203/04 (H5N1)	recombinant vaccine	rg-A/Hong Kong/213/03	rg-A/Hong Kong/213/03 + adjuvant	1
Forrest (3)	2009	H5N1 vaccine	A/Vietnam/1203/04 (H5N1)	recombinant vaccine	rg-A/Japanese white eye/Hong Kong/1038/06	rg-A/Japanese white eye/Hong Kong/1038/06 + adjuvant	1
Forrest (4)	2009	H5N1 vaccine	A/Vietnam/1203/04 (H5N1)	recombinant vaccine	rg-A/Vietnam/1203/04, rg-A/Hong Kong/213/03, rg-A/Japanese white eye/Hong Kong/1038/06, and rg-A/Whooper Swan/Mongolia/244/05	Multiple-clade+ Adjuvant	1
Forrest (5)	2009	H5N1 vaccine	A/Vietnam/1203/04 (H5N1)	recombinant vaccine	rg-A/Vietnam/1203/04	rg-A/Vietnam/1203/04 (no adjuvant)	1
Forrest (6)	2009	H5N1 vaccine	A/Vietnam/1203/04 (H5N1)	recombinant vaccine	rg-A/Hong Kong/213/03	rg-A/Hong Kong/213/03 (no adjuvant)	1
Forrest (7)	2009	H5N1 vaccine	A/Vietnam/1203/04 (H5N1)	recombinant vaccine	rg-A/Japanese white eye/Hong Kong/1038/06	rg-A/Japanese white eye/Hong Kong/1038/06 (no adjuvant)	1
Forrest (8)	2009	H5N1 vaccine	A/Vietnam/1203/04 (H5N1)	recombinant vaccine	rg-A/Vietnam/1203/04, rg-A/Hong Kong/213/03, rg-A/Japanese white eye/Hong Kong/1038/06, and rg-A/Whooper Swan/Mongolia/244/05	Multiple-clade (no adjuvant)	1
Haynes (1)	2009	seasonal influenza	A/Vietnam/1203/04 (H5N1)	virus-like particles (VLPs)	A/PR/8/34 (H1N1)	A/PR/8/34	1

		vaccine					
Haynes (2)	2009	H5N1 vaccine	A/Vietnam/1203/04 (H5N1)	virus-like particles (VLPs)	A/Indonesia/5/2005 (H5N1)	A/Indonesia/5/2005	1
Haynes (3)	2009	H5N1 vaccine	A/Vietnam/1203/04 (H5N1)	virus-like particles (VLPs)	A/Vietnam/1203/2004 (H5N1)	A/Vietnam/1203/2004	1
Middleton (1)	2009	H5N1 vaccine	A/Vietnam/1194/04 (H5N1)	split-virion vaccine	A/Vietnam/1194/04 (H5N1)	7.5 µg	1
Middleton (2)	2009	H5N1 vaccine	A/Vietnam/1194/04 (H5N1)	split-virion vaccine	A/Vietnam/1194/04 (H5N1)	15 µg	1
Middleton (3)	2009	H5N1 vaccine	A/Vietnam/1194/04 (H5N1)	split-virion vaccine	A/Vietnam/1194/04 (H5N1)	7.5 µg + AlPO4	1
Middleton (4)	2009	H5N1 vaccine	A/Vietnam/1194/04 (H5N1)	split-virion vaccine	A/Vietnam/1194/04 (H5N1)	15 µg + AlPO4	1
Middleton (5)	2009	H5N1 vaccine	A/Indonesia/05/05 (H5N1)	split-virion vaccine	A/Vietnam/1194/04 (H5N1)	15 µg + AlPO4	1
Middleton (6)	2009	H5N1 vaccine	A/Indonesia/05/05 (H5N1)	split-virion vaccine	A/Vietnam/1194/04 (H5N1)	15 µg + IMX	1
Middleton (7)	2009	H5N1 vaccine	A/Indonesia/05/05 (H5N1)	split-virion vaccine	A/Vietnam/1194/04 (H5N1)	3.8 µg + IMX	1
Middleton (8)	2009	H5N1 vaccine	A/Vietnam/1194/04 (H5N1)	split-virion vaccine	A/Vietnam/1194/04 (H5N1)	3.8 µg + IMX (2 doses)	1
Middleton (9)	2009	H5N1 vaccine	A/Vietnam/1194/04 (H5N1)	split-virion vaccine	A/Vietnam/1194/04 (H5N1)	30 µg + IMX	0
Middleton (10)	2009	H5N1 vaccine	A/Vietnam/1194/04 (H5N1)	split-virion vaccine	A/Vietnam/1194/04 (H5N1)	30 µg + AlPO4	0
Middleton (11)	2009	H5N1 vaccine	A/Vietnam/1194/04 (H5N1)	split-virion vaccine	A/Vietnam/1194/04 (H5N1)	30 µg	0
Middleton (12)	2009	H5N1 vaccine	A/Vietnam/1194/04 (H5N1)	split-virion vaccine	A/Vietnam/1194/04 (H5N1)	15 µg + IMX	0
Middleton (13)	2009	H5N1 vaccine	A/Vietnam/1194/04 (H5N1)	split-virion vaccine	A/Vietnam/1194/04 (H5N1)	7.5 µg + IMX	0
Middleton (14)	2009	H5N1 vaccine	A/Vietnam/1194/04 (H5N1)	split-virion vaccine	A/Vietnam/1194/04 (H5N1)	3.8 µg + IMX	0
Middleton (15)	2009	H5N1 vaccine	A/Vietnam/1194/04 (H5N1)	split-virion vaccine	A/Vietnam/1194/04 (H5N1)	15 µg + AlPO4	0
Middleton (16)	2009	H5N1 vaccine	A/Vietnam/1194/04 (H5N1)	split-virion vaccine	A/Vietnam/1194/04 (H5N1)	7.5 µg + AlPO4	0
Middleton (17)	2009	H5N1 vaccine	A/Vietnam/1194/04 (H5N1)	split-virion vaccine	A/Vietnam/1194/04 (H5N1)	3.8 µg + AlPO4	0
Perrone (1)	2009	seasonal influenza	A/Vietnam/1203/2004 (H5N1)	virus-like particles (VLPs)	A/South Carolina/1/18 (H1N1) and A/Brevig Mission/1/18 (H1N1)	intranasal	1

		vaccine					
Perrone (2)	2009	seasonal influenza vaccine	A/Vietnam/1203/2004 (H5N1)	virus-like particles (VLPs)	A/South Carolina/1/18 (H1N1) and A/Brevig Mission/1/18 (H1N1)	intramuscular	1
Price (1)	2009	seasonal influenza vaccine	A/Vietnam/1203/04 (H5N1)	DNA/ recombinant adenovirus vaccine	A/PR/8/34 (H1N1)	intranasal	1
Price (2)	2009	seasonal influenza vaccine	A/Vietnam/1203/04 (H5N1)	DNA/ recombinant adenovirus vaccine	A/PR/8/34 (H1N1)	intramuscular	1
Shoji (1)	2009	H5N1 vaccine	A/Indonesia/05/05 (H5N1)	recombinant subunit vaccine	A/Indonesia/05/2005	45 μg	2
Shoji (2)	2009	H5N1 vaccine	A/Indonesia/05/05 (H5N1)	recombinant subunit vaccine	A/Indonesia/05/2005	90 μg	2
Song (1)	2009	H5N1 vaccine	A/EM/Korea/W149/06	recombinant vaccine	A/EM/Korea/W149/06	RgKoreaES223N/03xPR8 3.5 μg	1
Song (2)	2009	H5N1 vaccine	A/EM/Korea/W149/06	recombinant vaccine	A/EM/Korea/W149/06	RgKoreaES223N/03xPR8 7.5 μg	1
Song (3)	2009	H5N1 vaccine	A/EM/Korea/W149/06	recombinant vaccine	A/EM/Korea/W149/06	RgKoreaES223N/03xPR8 15 μg	1
Song (4)	2009	H5N1 vaccine	A/EM/Korea/W149/06	recombinant vaccine	A/Ck/Korea/ES/03	RgKoreaW149/06xPR8 3.5 μg	1
Song (5)	2009	H5N1 vaccine	A/EM/Korea/W149/06	recombinant vaccine	A/Ck/Korea/ES/03	RgKoreaW149/06xPR8 7.5 μg	1

Song (6)	2009	H5N1 vaccine	A/EM/Korea/W149/06	recombinant vaccine	A/Ck/Korea/ES/03	RgKoreaW149/06xPR8 15 µg	1
Rao (1)	2010	H5N1 vaccine	A/Vietnam/1203/2004 (H5N1)	DNA vaccine/ non-replicating adenovirus vaccine	A/Thailand/1(KAN- 1)/2004	HA	3
Rao (2)	2010	H5N1 vaccine	A/Vietnam/1203/2004 (H5N1)	DNA vaccine/ non-replicating adenovirus vaccine	A/Thailand/1(KAN- 1)/2004	HA+NP+M2	3
Rao (3)	2010	H5N1 vaccine	A/Vietnam/1203/2004 (H5N1)	DNA vaccine/ non-replicating adenovirus vaccine	A/Thailand/1(KAN- 1)/2004	NP	3
Rao (4)	2010	H5N1 vaccine	A/Vietnam/1203/2004 (H5N1)	DNA vaccine/ non-replicating adenovirus vaccine	A/Thailand/1(KAN- 1)/2004	NP+M2	3
Rao (5)	2010	H5N1 vaccine	A/Vietnam/1203/2004 (H5N1)	DNA vaccine/ non-replicating adenovirus vaccine	A/Thailand/1(KAN- 1)/2004	M2	3
Gustin (1)	2011	H5N1 vaccine	A/Vietnam/1203/2004 (H5N1)	recombinant vaccine	A/Vietnam/1203/2004 (H5N1) and (ca/ts)A/Leningrad/134/17/57 (H2N2)	LAIV	1
Gustin (2)	2011	H5N1 vaccine	A/Vietnam/1203/2004 (H5N1)	recombinant vaccine	A/Vietnam/1203/2004 (H5N1) and (ca/ts)A/Leningrad/134/17/57 (H2N2)	IIV	1
Gustin (3)	2011	H5N1 vaccine	A/Egypt/2321-NAMRU3/2007	recombinant vaccine	A/Vietnam/1203/2004 (H5N1) and (ca/ts)A/Leningrad/134/17/57 (H2N2)	LAIV	1
Gustin (4)	2011	H5N1 vaccine	A/Egypt/2321-NAMRU3/2007	recombinant vaccine	A/Vietnam/1203/2004 (H5N1) and	IIV	1

					(ca/ts)A/Leningrad/134/17/57 (H2N2)		
Layton (1)	2011	H5N1 vaccine	A/Vietnam/1203/ 2004 (H5N1)	split-virion vaccine	A/Vietnam/1203/ 2004 (H5N1)	7.5 μg + Adjuvant 1 (two doses)	1
Layton (2)	2011	H5N1 vaccine	A/Vietnam/1203/2004 (H5N1)	split-virion vaccine	A/Vietnam/1203/ 2004 (H5N1)	7.5 μg + Adjuvant 2 (two doses)	1
Layton (3)	2011	H5N1 vaccine	A/Vietnam/1203/2004 (H5N1)	split-virion vaccine	A/Vietnam/1203/ 2004 (H5N1)	22.5 μg + Adjuvant 1 (two doses)	1
Layton (4)	2011	H5N1 vaccine	A/Vietnam/1203/2004 (H5N1)	split-virion vaccine	A/Vietnam/1203/ 2004 (H5N1)	22.5 μg + Adjuvant 2 (two doses)	1
Layton (5)	2011	H5N1 vaccine	A/Vietnam/1203/2004 (H5N1)	split-virion vaccine	A/Vietnam/1203/ 2004 (H5N1)	22.5 μg + Ad2 (one dose)	0
Layton (6)	2011	H5N1 vaccine	A/Vietnam/1203/2004 (H5N1)	split-virion vaccine	A/Vietnam/1203/ 2004 (H5N1)	30 μg (No adjuvant)	1
Layton (7)	2011	H5N1 vaccine	A/Vietnam/1203/2004 (H5N1)	split-virion vaccine	A/Vietnam/1203/ 2004 (H5N1)	7.5 μg (No adjuvant)	1
Layton (8)	2011	H5N1 vaccine	A/Vietnam/1203/2004 (H5N1)	split-virion vaccine	A/Vietnam/1203/ 2004 (H5N1)	1.9 μg (No adjuvant)	1
Layton (9)	2011	H5N1 vaccine	A/Vietnam/1203/2004 (H5N1)	split-virion vaccine	A/Vietnam/1203/ 2004 (H5N1)	22.5 μg + adjuvant	1
Layton (10)	2011	H5N1 vaccine	A/Vietnam/1203/2004 (H5N1)	split-virion vaccine	A/Vietnam/1203/ 2004 (H5N1)	7.5 μg + adjuvant	1
Layton (11)	2011	H5N1 vaccine	A/Vietnam/1203/2004 (H5N1)	split-virion vaccine	A/Vietnam/1203/ 2004 (H5N1)	22.5 μg	1
Ducatez (1)	2012	H5N1 vaccine	A/Vietnam/1203/2004 (H5N1)	inactivated whole-virus vaccines	A/Vietnam/1203/ 04 (H5N1)	VN1203	2
Ducatez (2)	2012	H5N1 vaccine	A/Vietnam/1203/2004 (H5N1)	inactivated whole-virus vaccines	A／duck／Hunan／795／02	DKHUN795	2
Ducatez (3)	2012	H5N1 vaccine	A/Vietnam/1203/2004 (H5N1)	inactivated whole-virus vaccines	reconstructed H5N1 ancestral sequences	A	2
Ducatez (4)	2012	H5N1 vaccine	A/Vietnam/1203/2004 (H5N1)	inactivated whole-virus	reconstructed H5N1 ancestral sequences	D	2

				vaccines			
Liu (1)	2012	H5N1 vaccine	A/Vietnam/1203/2004 (H5N1)	recombinant H5 HA vaccine from H5N1	A/Indonesia/5/2005, A/Anhui/1/2005, or A/Vietnam/1203/2004	R3. HA5VN 15 µg	1
Liu (2)	2012	H5N1 vaccine	A/Vietnam/1203/2004 (H5N1)	recombinant H5 HA vaccine from H5N2	A/Indonesia/5/2005, A/Anhui/1/2005, or A/Vietnam/1203/2004	R3. HA5VN 45 µg	1
Liu (3)	2012	H5N1 vaccine	A/Vietnam/1203/2004 (H5N1)	recombinant H5 HA vaccine from H5N3	A/Indonesia/5/2005, A/Anhui/1/2005, or A/Vietnam/1203/2004	R3.2xH5VN 15 µg	1
Liu (4)	2012	H5N1 vaccine	A/Vietnam/1203/2004 (H5N1)	recombinant H5 HA vaccine from H5N4	A/Indonesia/5/2005, A/Anhui/1/2005, or A/Vietnam/1203/2004	R3.2xH5VN 45 µg	1
Scallan (1)	2012	H5N1 vaccine	A/Indonesia/05/05 (H5N1)	Adenovirus-based Vaccine	A/Indo/05/2005	IM rAd-HA-dsDNA	1
Scallan (2)	2012	H5N1 vaccine	A/Indonesia/05/05 (H5N1)	Adenovirus-Based Vaccine	A/Indo/05/2005	PO rAd-HA-dsDNA	1
Vela (1)	2012	H5N1 vaccine	A/Vietnam/1203/2004 (H5N1)	inactivated monovalent subvirion vaccines	A／Indonesia／05／05	CLAG-1324	1
Vela (2)	2012	H5N1 vaccine	A/Vietnam/1203/2004 (H5N1)	inactivated monovalent subvirion vaccines	A／Indonesia／05／05	CLAG-1323	1
Vela (3)	2012	H5N1 vaccine	A/Vietnam/1203/2004 (H5N1)	inactivated monovalent subvirion vaccines	A／Indonesia／05／05	CLAG-1324 + CLAG-1073	1
Vela (4)	2012	H5N1 vaccine	A/Vietnam/1203/2004 (H5N1)	inactivated monovalent subvirion vaccines	A／Indonesia／05／05	CLAG-1323 + CLAG-1073	1
Verma (1)	2012	H5N1 vaccine	A/Vietnam/1203/2004 (H5N1)	recombinant vaccine	A/Vietnam/1203/2004 (H5N1)	monomeric HA1	1
Verma (2)	2012	H5N1 vaccine	A/Vietnam/1203/2004 (H5N1)	recombinant vaccine	A/Vietnam/1203/2004 (H5N1)	oligomeric HA1	1
Verma (3)	2012	H5N1 vaccine	A/Vietnam/1203/2004 (H5N1)	egg-based licensed	A/Vietnam/1203/2004 (H5N1)	SU-H5N1 vaccines	1

				subunit H5N1			
Verma (4)	2012	H5N1 vaccine	A/Whooper Swan/Mongolia/244/2005 (H5N1)	recombinant vaccine	A/Vietnam/1203/2004 (H5N1)	monomeric HA1	1
Verma (5)	2012	H5N1 vaccine	A/Whooper Swan/Mongolia/244/2005 (H5N1)	recombinant vaccine	A/Vietnam/1203/2004 (H5N1)	oligomeric HA1	1
Verma (6)	2012	H5N1 vaccine	A/Whooper Swan/Mongolia/244/2005 (H5N1)	recombinant vaccine	A/Vietnam/1203/2004 (H5N1)	SU-H5N1 vaccines	1
Rockman (1)	2013	seasonal influenza vaccine	A/Vietnam/1203/2004 (H5N1)	split-virion vaccine	A/New Caledonia/20/1999 (H1N1), A/Brisbane/59/ 2007 (H1N1), A/Hiroshima/52/2005 (H3N2), and B/Malaysia/2506/2004	Fluvax	1
Rockman (2)	2013	seasonal influenza vaccine	A/Vietnam/1203/2004 (H5N1)	split-virion vaccine	A/New Caledonia/20/1999 (H1N1), A/Brisbane/59/ 2007 (H1N1), A/Hiroshima/52/2005 (H3N2), and B/Malaysia/2506/2004	Fluvax + AIPO4	1
Rockman (3)	2013	seasonal influenza vaccine	A/Vietnam/1203/2004 (H5N1)	split-virion vaccine	A/New Caledonia/20/1999 (H1N1), A/Brisbane/59/ 2007 (H1N1), A/Hiroshima/52/2005 (H3N2), and B/Malaysia/2506/2004	Fluvax + IMX	1
Rockman (4)	2013	seasonal influenza vaccine	A/Vietnam/1203/2004 (H5N1)	split-virion vaccine	A/New Caledonia/20/99 (H1N1)	H1N1 + IMX	1
Rockman (5)	2013	seasonal influenza	A/Vietnam/1203/2004 (H5N1)	split-virion vaccine	A/Wisconsin/67/2005 (H3N2)	H3N2 + IMX	1

		vaccine					
Rockman (6)	2013	seasonal influenza vaccine	A/Vietnam/1203/2004 (H5N1)	split-virion vaccine	A/Brisbane/59/ 2007 (H1N1)	H1N1 (HA 15 μg)	1
Rockman (7)	2013	seasonal influenza vaccine	A/Vietnam/1203/2004 (H5N1)	split-virion vaccine	A/Brisbane/59/ 2007 (H1N1)	H1N1 (HA 30 μg)	1
Rockman (8)	2013	seasonal influenza vaccine	A/Vietnam/1203/2004 (H5N1)	split-virion vaccine	A/Wisconsin/67/2005 (H3N2) and A/New Caledonia/20/1999 (H1N1)	H3N1 (HA 30 μg)	1
Rockman (9)	2013	seasonal influenza vaccine	A/Vietnam/1203/2004 (H5N1)	split-virion vaccine	A/Wisconsin/67/2005 (H3N2)	H3N2 (HA 30 μg)	1
Rockman (10)	2013	seasonal influenza vaccine	A/Vietnam/1203/2004 (H5N1)	subunit vaccine	A/Brisbane/59/ 2007 (H1N1)	pH1 HA	1
Rockman (11)	2013	seasonal influenza vaccine	A/Vietnam/1203/2004 (H5N1)	subunit vaccine	A/Brisbane/59/ 2007 (H1N1)	rN1 NA	1
Rockman (12)	2013	seasonal influenza vaccine	A/Vietnam/1203/2004 (H5N1)	subunit vaccine	A/Brisbane/59/ 2007 (H1N1)	pH1 HA + rN1 NA	1
Rockman (13)	2013	seasonal influenza	A/Vietnam/1203/2004 (H5N1)	subunit vaccine	A/Wisconsin/67/2005 (H3N2)	pH3 HA	1

		vaccine					
Rockman (14)	2013	seasonal influenza vaccine	A/Vietnam/1203/2004 (H5N1)	split-virion vaccine	A/Brisbane/59/ 2007 (H1N1)	Split H1N1	1
Mann (1)	2014	H5N1 vaccine	A/Vietnam/1194/2004 (H5N1)	subunit vaccine	NIBRG-14(H5N1 and H1N1)	Unadjuvanted (IT challenge)	1
Mann (2)	2014	H5N1 vaccine	A/Vietnam/1194/2004 (H5N1)	subunit vaccine	NIBRG-14(H5N1 and H1N1)	CSN adjuvanted (IT challenge)	1
Mann (3)	2014	H5N1 vaccine	A/Vietnam/1194/2004 (H5N1)	subunit vaccine	NIBRG-14(H5N1 and H1N1)	TM-CSN adjuvanted (IT challenge)	1
Park (1)	2014	seasonal influenza vaccine	A/Vietnam/1203/2004 (H5N1)	live virus	A/California/07/2009 (H1N1)	H1N1 (group B)	0
Park (2)	2014	seasonal influenza vaccine	A/Vietnam/1203/2004 (H5N1)	live virus	A/Perth/16/2009 (H3N2)	H3N2 (group C)	0
Park (3)	2014	seasonal influenza vaccine	A/Vietnam/1203/2004 (H5N1)	inactivated vaccine	A/California/07/2009 (H1N1), A/Victoria/210/2009 (H3N2) and B/Brisbane/60/ 2008	seasonal TIV (group D)	2
Baz (1)	2015	H5N1 vaccine	A/Vietnam/1203/2004 (H5N1)	virus-like particles (VLPs)	A/Vietnam/1203/2004 (H5N1), A/New York/107/2003 (H7N2) and A/Hong Kong/33982/2009 (H9N2) viruses	VLPs	1
Cox (1)	2015	H5N1 vaccine	A/Vietnam/1203/2004 (H5N1)	recombinant vaccine	human A/Vietnam/1194/2004 (H5N1) and A/Puerto Rico/8/34 (H1N1) (PR8) strains	30 µg HA	1

Cox (2)	2015	H5N1 vaccine	A/Vietnam/1203/2004 (H5N1)	recombinant vaccine	human A/Vietnam/1194/2004 (H5N1) and A/Puerto Rico/8/34 (H1N1) (PR8) strains	1.5 μg HA + Matrix M	1
Cox (3)	2015	H5N1 vaccine	A/Vietnam/1203/2004 (H5N1)	recombinant vaccine	human A/Vietnam/1194/2004 (H5N1) and A/Puerto Rico/8/34 (H1N1) (PR8) strains	7.5 μg HA+ Matrix M	1
Cox (4)	2015	H5N1 vaccine	A/Vietnam/1203/2004 (H5N1)	recombinant vaccine	human A/Vietnam/1194/2004 (H5N1) and A/Puerto Rico/8/34 (H1N1) (PR8) strains	30 μg HA + Matrix M	1
Major (1)	2015	H5N1 vaccine	A/Indonesia/05/05 (H5N1)	recombinant vaccine	A/Indonesia/05/05(H5N1)	HA	1
Major (2)	2015	H5N1 vaccine	A/Indonesia/05/05 (H5N1)	recombinant vaccine	A/Indonesia/05/05(H5N1)	HA+c-di-GMP	1
Liu (1)	2016	H5N1 vaccine	A/Indonesia/05/05 (H5N1)	split-virion vaccine	A/bar-headed goose/Qinghai/1A/2005	7.5 μg HA+JVRS-100	1
Liu (2)	2016	H5N1 vaccine	A/Indonesia/05/05 (H5N1)	split-virion vaccine	A/bar-headed goose/Qinghai/1A/2005	7.5 μg HA	1
Hatta (1)	2017	seasonal influenza vaccine	A/Vietnam/1203/2004 (H5N1)	live virus	A/California/07/2009 (H1N1)	M2SR H1N1, 2 doses	1
Hatta (2)	2017	seasonal influenza vaccine	A/Vietnam/1203/2004 (H5N1)	live virus	A/California/07/2009 (H1N1)	M2SR H1N1, 1 dose	0
Hatta (3)	2017	H5N1 vaccine	A/Vietnam/1203/2004 (H5N1)	live virus	A/Vietnam/1203/2004 (H5N1)	M2SR H5N1, 2 doses	1
Hatta (4)	2017	H5N1 vaccine	A/Vietnam/1203/2004 (H5N1)	live virus	A/Vietnam/1203/2004 (H5N1)	M2SR H5N1, 1 dose	0
Wong (1)	2017	H5N1 vaccine	A/Vietnam/1203/2004 (H5N1)	split-virion vaccine	A/Vietnam/1203/2004 (H5N1)	no adjuvant	1
Wong (2)	2017	H5N1 vaccine	A/Vietnam/1203/2004 (H5N1)	split-virion vaccine	A/Vietnam/1203/2004 (H5N1)	MF59 (low titer)	1
Wong (3)	2017	H5N1 vaccine	A/Vietnam/1203/2004 (H5N1)	split-virion vaccine	A/Vietnam/1203/2004 (H5N1)	AS03 (high titer)	1
Smith (1)	2019	H5N1 vaccine	A/Indonesia/05/2005 (H5N1)	recombinant vaccine	A/Indonesia/05/2005	intranasal rH5-NE01 x2	1
Smith (2)	2019	H5N1 vaccine	A/Indonesia/05/2005 (H5N1)	recombinant vaccine	A/Indonesia/05/2005	intranasal rH5-NE01 x1	0
Smith (3)	2019	H5N1 vaccine	A/Indonesia/05/2005 (H5N1)	recombinant vaccine	A/Indonesia/05/2005	intranasal rH5-NE01(45	1

						mg/dose) x2	
Smith (4)	2019	H5N1 vaccine	A/Vietnam/1203/2004 (H5N1)	recombinant vaccine	A/Indonesia/05/2005	intranasal rH5-NE01(45 mg/dose) x2	1
Wang (1)	2019	H5N1 vaccine	A/Indonesia/05/05 (H5N1)	recombinant vaccine	A/Indonesia/05/05(H5N1)	20 µg rH5 + NE01	2
Wang (2)	2019	H5N1 vaccine	A/Indonesia/05/05 (H5N1)	recombinant vaccine	A/Indonesia/05/05(H5N1)	20 µg rH5	2
Schön (1)	2020	H5N1 vaccine	A/Cygnus cygnus/Germany/R65/2006(H5N1)	recombinant vaccine	A/swan/Germany/R65/2006 (H5N1)	R65 mono/H17N10	1
Furey	2024	H5N1 vaccine	A/bald eagle/Florida/W22-134-OP/2022 (H5N1)	mRNA vaccine	A/Astrakhan/3212/2020	H5 mRNA-LNP	1
Hatta (1)	2024	H5N1 vaccine	A/bald eagle/Florida/22–006544-004/2022	mRNA vaccine	A/American wigeon/South Carolina/22–000345-001/2021	50 µg HA-WT mRNA	2
Hatta (2)	2024	H5N1 vaccine	A/bald eagle/Florida/22–006544-004/2022	mRNA vaccine	A/American wigeon/South Carolina/22–000345-001/2021	50 µg HA-del mRNA	2
Hatta (3)	2024	H5N1 vaccine	A/bald eagle/Florida/22–006544-004/2022	mRNA vaccine	A/American wigeon/South Carolina/22–000345-001/2021	50 µg HA-del + NA mRNA	2
Hatta (4)	2024	H5N1 vaccine	A/bald eagle/Florida/22–006544-004/2022	mRNA vaccine	A/American wigeon/South Carolina/22–000345-001/2021	100 µg HA-del + NA mRNA	2
Hatta (5)	2024	H5N1 vaccine	A/bald eagle/Florida/22–006544-004/2022	recombinant protein	A/American wigeon/South Carolina/22–000345-001/2021	50 µg + adjuvant	2
Hatta (6)	2024	H5N1 vaccine	A/bald eagle/Florida/22–006544-004/2022	mRNA vaccine	A/American wigeon/South Carolina/22–000345-001/2021	50 µg HA-WT mRNA	2
Hatta (7)	2024	H5N1 vaccine	A/bald eagle/Florida/22–006544-004/2022	mRNA vaccine	A/American wigeon/South Carolina/22–000345-001/2021	50 µg HA-WT + NA mRNA	2

Hatta (8)	2024	H5N1 vaccine	A/bald eagle/Florida/22–006544-004/2022	recombinant protein	A/American wigeon/South Carolina/22–000345-001/2021	50 µg H5-del	2

Supplementary Table 3. Study characteristics of trials evaluating the vaccine efficacy of seasonal influenza and H5N1 vaccines in ferrets. One published study may report multiple trials, which were labeled in the order described in the article with numbers in parentheses.

(b) Numbers of animals in control and vaccinated groups, numbers of events in each group, study design, serological assay method, post-vaccination antibody titers, and seroprotection status.

Note: "Survival rate" refers to the proportion of vaccinated ferrets that survived the lethal challenge; "Time Interval" = time interval from vaccination to challenge (day); "Test Virus" denotes the virus strain used to measure seroprotection; "Seroprotection" indicates whether the vaccinated group achieved seroprotective antibody levels before challenge; HI = hemagglutination inhibition assay; MN = microneutralization assay; MDCK = Madin–Darby canine kidney cell; NA = not available.

Trial	Control (total)	Vaccine (total)	Control (survival)	Vaccine (survival)	Survival rate	Time Interval	Method	Cell	Test Virus	Post-vaccination Titer	Seroprotection Status
Hoffmann (1)	3	3	0	3	1	14	HI	chicken RBC	A/Vietnam/1203/2004	16	not achieving
Hoffmann (2)	3	3	0	3	1	14	HI	chicken RBC	A/Vietnam/1203/2004	20	not achieving
Hoffmann (3)	3	3	0	3	1	14	HI	chicken RBC	A/Vietnam/1203/2004	254	achieving
Govorkova (1)	3	3	0	3	1	14	HI	chicken RBC	A/Vietnam/1203/2004	0	not achieving
Govorkova (2)	3	3	0	3	1	14	HI	chicken RBC	A/Vietnam/1203/2004	6	not achieving
Govorkova (3)	3	3	0	3	1	14	HI	chicken RBC	A/Vietnam/1203/2004	0	not achieving
Baras (1)	6	6	0	0	0	14	MN	NA	A/Indonesia/05/2005	14	not achieving
Baras (2)	6	6	0	5	0.83	14	MN	NA	A/Indonesia/05/2005	36	not achieving
Baras (3)	6	6	0	6	1	14	MN	NA	A/Indonesia/05/2005	43	achieving

Baras (4)	6	5	0	5	1	14	MN	NA	A/Indonesia/05/2005	35	not achieving
Baras (5)	6	6	0	6	1	14	MN	NA	A/Indonesia/05/2005	26	not achieving
Lalor (1)	6	6	0	1	0.17	14	HI	NA	A/Vietnam/1203/ 2004	10	not achieving
Lalor (2)	6	6	0	6	1	14	HI	NA	A/Vietnam/1203/ 2004	640	achieving
Lalor (3)	6	6	0	6	1	14	HI	NA	A/Vietnam/1203/ 2004	226	achieving
Lalor (4)	6	6	0	6	1	14	HI	NA	A/Vietnam/1203/ 2004	10	not achieving
Mahmood (1)	4	4	1	4	1	14	HI	horse RBC	A/Indonesia/05/2005	0	not achieving
Mahmood (2)	4	4	1	4	1	14	HI	horse RBC	A/Indonesia/05/2005	145	achieving
Mahmood (3)	4	4	1	4	1	14	HI	horse RBC	A/Indonesia/05/2005	143	achieving
Mahmood (4)	4	4	1	4	1	14	HI	horse RBC	A/Indonesia/05/2005	160	achieving
Mahmood (5)	4	4	1	4	1	14	HI	horse RBC	A/Indonesia/05/2005	453	achieving
Mahmood (6)	4	4	0	4	1	14	HI	horse RBC	A/Vietnam/1203/2004	57	achieving
Mahmood (7)	4	4	0	4	1	14	HI	horse RBC	A/Vietnam/1203/2004	160	achieving
Mahmood (8)	4	4	0	4	1	14	HI	horse RBC	A/Vietnam/1203/2004	57	achieving
Mahmood (9)	4	4	0	4	1	14	HI	horse RBC	A/Vietnam/1203/2004	40	not achieving
Mahmood (10)	4	4	0	4	1	14	HI	horse RBC	A/Vietnam/1203/2004	80	achieving
Forrest (1)	5	5	0	5	1	14	HI	chicken RBC	Rg-A/Vietnam/1203/2004	194	achieving

Forrest (2)	5	5	0	5	1	14	HI	chicken RBC	Rg-A/Vietnam/1203/2004	42	achieving
Forrest (3)	5	5	0	5	1	14	HI	chicken RBC	Rg-A/Vietnam/1203/2004	10	not achieving
Forrest (4)	5	5	0	5	1	14	HI	chicken RBC	Rg-A/Vietnam/1203/2004	57	achieving
Forrest (5)	5	5	0	5	1	14	HI	chicken RBC	Rg-A/Vietnam/1203/2004	60	achieving
Forrest (6)	5	5	0	5	1	14	HI	chicken RBC	Rg-A/Vietnam/1203/2004	13	not achieving
Forrest (7)	5	5	0	5	1	14	HI	chicken RBC	Rg-A/Vietnam/1203/2004	5	not achieving
Forrest (8)	5	5	0	5	1	14	HI	chicken RBC	Rg-A/Vietnam/1203/2004	23	not achieving
Haynes (1)	7	7	1	7	1	14	HI	horse RBC	A/Vietnam/1203/2004	29	not achieving
Haynes (2)	7	7	1	7	1	14	HI	horse RBC	A/Vietnam/1203/2004	251	achieving
Haynes (3)	7	7	1	7	1	14	HI	horse RBC	A/Vietnam/1203/2004	217	achieving
Middleton (1)	4	4	1	2	0.5	14	HI	chicken RBC	A/Vietnam/1203/2004	<4	not achieving
Middleton (2)	4	4	1	1	0.25	14	HI	chicken RBC	A/Vietnam/1203/2004	<4	not achieving
Middleton (3)	4	4	1	2	0.5	14	HI	chicken RBC	A/Vietnam/1203/2004	27	not achieving

Middleton (4)	4	4	1	4	1	14	HI	chicken RBC	A/Vietnam/1203/2004	48	achieving
Middleton (5)	4	4	0	4	1	14	HI	chicken RBC	A/Indonesia/05/2005	27	not achieving
Middleton (6)	4	3	0	3	1	14	HI	chicken RBC	A/Indonesia/05/2005	64	achieving
Middleton (7)	4	4	0	4	1	14	HI	chicken RBC	A/Indonesia/05/2005	19	not achieving
Middleton (8)	4	4	0	4	1	14	HI	chicken RBC	A/Vietnam/1203/2004	16	not achieving
Middleton (9)	4	4	0	4	1	14	HI	chicken RBC	A/Vietnam/1203/2004	8	not achieving
Middleton (10)	4	4	0	4	1	14	HI	chicken RBC	A/Vietnam/1203/2004	19	not achieving
Middleton (11)	4	4	0	4	1	14	HI	chicken RBC	A/Vietnam/1203/2004	<4	not achieving
Middleton (12)	4	4	1	4	1	14	HI	chicken RBC	A/Vietnam/1203/2004	19	not achieving
Middleton (13)	4	4	1	4	1	14	HI	chicken RBC	A/Vietnam/1203/2004	14	not achieving
Middleton (14)	4	4	1	4	1	14	HI	chicken RBC	A/Vietnam/1203/2004	10	not achieving
Middleton (15)	4	4	1	4	1	14	HI	chicken RBC	A/Vietnam/1203/2004	16	not achieving

Middleton (16)	4	4	1	3	0.75	14	HI	chicken RBC	A/Vietnam/1203/2004	7	not achieving
Middleton (17)	4	4	1	3	0.75	14	HI	chicken RBC	A/Vietnam/1203/2004	11	not achieving
Perrone (1)	6	6	0	6	1	14	HI	horse RBC	A/Vietnam/1203/2004	10	not achieving
Perrone (2)	6	6	0	3	0.5	14	HI	horse RBC	A/Vietnam/1203/2004	10	not achieving
Price (1)	6	6	0	5	0.83	14	NA	NA	NA	NA	not achieving
Price (2)	6	6	0	6	1	14	NA	NA	NA	NA	not achieving
Shoji (1)	10	10	5	10	1	14	HI	horse RBC	A/Indonesia/05/2005	520	achieving
Shoji (2)	10	10	5	10	1	14	HI	horse RBC	A/Indonesia/05/2005	781	achieving
Song (1)	4	4	1	4	1	14	HI	chicken RBC	A/EM/Korea/W149/06	29	not achieving
Song (2)	4	4	1	4	1	14	HI	chicken RBC	A/EM/Korea/W149/06	81	achieving
Song (3)	4	4	1	4	1	14	HI	chicken RBC	A/EM/Korea/W149/06	197	achieving
Song (4)	4	4	1	4	1	14	HI	chicken RBC	A/EM/Korea/W149/06	40	not achieving
Song (5)	4	4	1	4	1	14	HI	chicken RBC	A/EM/Korea/W149/06	162	achieving
Song (6)	4	4	1	4	1	14	HI	chicken RBC	A/EM/Korea/W149/06	324	achieving
Rao (1)	4	4	0	4	1	14	HI	horse RBC	H5N1-PR8 re-assortants	226	achieving
Rao (2)	4	4	0	4	1	14	HI	horse RBC	H5N1-PR8 re-assortants	905	achieving

Rao (3)	5	4	0	0	0	14	HI	horse RBC	H5N1-PR8 re-assortants	10	not achieving
Rao (4)	5	5	0	0	0	14	HI	horse RBC	H5N1-PR8 re-assortants	10	not achieving
Rao (5)	5	4	0	0	0	14	HI	horse RBC	H5N1-PR8 re-assortants	10	not achieving
Gustin (1)	6	6	0	6	1	14	HI	horse RBC	A/Vietnam/1203/2004	86	achieving
Gustin (2)	6	6	0	6	1	14	HI	horse RBC	A/Vietnam/1203/2004	635	achieving
Gustin (3)	6	6	3	6	1	14	HI	horse RBC	A/Egypt/2321-NAMRU3/2007	17	not achieving
Gustin (4)	6	5	3	5	1	14	HI	horse RBC	A/Egypt/2321-NAMRU3/2007	128	achieving
Layton (1)	3	7	0	7	1	14	HI	horse RBC	A/Vietnam/1203/2004	76	achieving
Layton (2)	3	7	0	7	1	14	HI	horse RBC	A/Vietnam/1203/2004	82	achieving
Layton (3)	3	7	0	7	1	14	HI	horse RBC	A/Vietnam/1203/2004	149	achieving
Layton (4)	3	7	0	7	1	14	HI	horse RBC	A/Vietnam/1203/2004	174	achieving
Layton (5)	3	6	0	6	1	14	HI	horse RBC	A/Vietnam/1203/2004	23	not achieving
Layton (6)	14	14	0	9	0.64	14	HI	horse RBC	A/Vietnam/1203/2004	10	not achieving
Layton (7)	14	14	0	6	0.43	14	HI	horse RBC	A/Vietnam/1203/2004	10	not achieving
Layton (8)	14	14	0	6	0.43	14	HI	horse RBC	A/Vietnam/1203/2004	10	not achieving
Layton (9)	14	14	0	13	0.93	14	HI	horse RBC	A/Vietnam/1203/2004	326	achieving
Layton (10)	14	14	0	13	0.93	14	HI	horse RBC	A/Vietnam/1203/2004	326	achieving
Layton (11)	14	14	0	0	0	14	HI	horse RBC	A/Vietnam/1203/2004	10	not achieving
Ducatez (1)	4	4	0	4	1	14	MN	MDCK	A/Vietnam/1203/2004	135	achieving
Ducatez (2)	4	4	0	4	1	14	MN	MDCK	A/Vietnam/1203/2004	63	achieving
Ducatez (3)	4	4	0	4	1	14	MN	MDCK	A/Vietnam/1203/2004	45	achieving
Ducatez (4)	4	4	0	4	1	14	MN	MDCK	A/Vietnam/1203/2004	120	achieving

Liu (1)	6	6	1	6	1	14	HI	horse RBC	A/Indonesia/05/2005	10	not achieving
Liu (2)	6	6	1	6	1	14	HI	horse RBC	A/Indonesia/05/2005	10	not achieving
Liu (3)	6	6	1	6	1	14	HI	horse RBC	A/Indonesia/05/2005	10	not achieving
Liu (4)	6	6	1	6	1	14	HI	horse RBC	A/Indonesia/05/2005	10	not achieving
Scallan (1)	4	8	1	8	1	14	HI	horse RBC	A/Indonesia/05/2005	65	achieving
Scallan (2)	4	8	1	6	0.75	14	HI	horse RBC	A/Indonesia/05/2005	90	achieving
Vela (1)	8	8	0	4	0.5	14	HI	horse RBC	A/Vietnam/1203/2004	5	not achieving
Vela (2)	8	8	0	2	0.25	14	HI	horse RBC	A/Vietnam/1203/2004	5	not achieving
Vela (3)	8	8	0	8	1	14	HI	horse RBC	A/Vietnam/1203/2004	6	not achieving
Vela (4)	8	8	0	8	1	14	HI	horse RBC	A/Vietnam/1203/2004	5	not achieving
Verma (1)	6	6	0	2	0.3	14	HI	human RBC	A/Vietnam/1203/2004	37	not achieving
Verma (2)	6	6	0	6	1	14	HI	human RBC	A/Vietnam/1203/2004	105	achieving
Verma (3)	6	6	0	6	1	14	HI	human RBC	A/Vietnam/1203/2004	39	not achieving
Verma (4)	6	6	0	1	0.17	14	HI	human RBC	A/Vietnam/1203/2004	10	not achieving
Verma (5)	6	6	0	6	1	14	HI	human RBC	A/Vietnam/1203/2004	90	achieving
Verma (6)	6	6	0	6	1	14	HI	human RBC	A/Vietnam/1203/2004	17	not achieving
Rockman (1)	4	4	0	2	0.5	14	HI	chicken RBC	A/Vietnam/1203/2004	< 4	not achieving

Rockman (2)	4	4	0	4	1	14	HI	chicken RBC	A/Vietnam/1203/2004	< 4	not achieving
Rockman (3)	4	4	0	4	1	14	HI	chicken RBC	A/Vietnam/1203/2004	< 4	not achieving
Rockman (4)	4	3	0	3	1	14	HI	chicken RBC	A/Vietnam/1203/2004	< 4	not achieving
Rockman (5)	4	4	0	0	0	14	HI	chicken RBC	A/Vietnam/1203/2004	< 4	not achieving
Rockman (6)	4	4	0	4	1	14	NA	NA	NA	NA	not achieving
Rockman (7)	4	4	0	4	1	14	NA	NA	NA	NA	not achieving
Rockman (8)	4	8	0	3	0.38	14	NA	NA	NA	NA	not achieving
Rockman (9)	4	8	0	1	0.13	14	NA	NA	NA	NA	not achieving
Rockman (10)	4	4	0	3	0.75	14	NA	NA	NA	NA	not achieving
Rockman (11)	4	4	0	4	1	14	NA	NA	NA	NA	not achieving
Rockman (12)	4	4	0	4	1	14	NA	NA	NA	NA	not achieving
Rockman (13)	4	4	0	1	0.25	14	NA	NA	NA	NA	not achieving
Rockman (14)	4	4	0	4	1	14	NA	NA	NA	NA	not achieving
Mann (1)	6	6	1	4	0.67	14	HI	horse RBC	A/Vietnam/1194/2004	2	not achieving
Mann (2)	6	6	1	6	1	14	HI	horse RBC	A/Vietnam/1194/2004	20	not achieving
Mann (3)	6	6	1	6	1	14	HI	horse RBC	A/Vietnam/1194/2004	188	achieving
Park (1)	12	12	0	12	1	14	HI	NA	A/Vietnam/1203/2004	10	not achieving
Park (2)	12	12	0	12	1	14	HI	NA	A/Vietnam/1203/2004	10	not achieving
Park (3)	12	12	0	0	0	14	HI	NA	A/Vietnam/1203/2004	10	not achieving
Baz (1)	4	4	0	4	1	14	HI	horse RBC	A/Vietnam/1203/2004	7	not achieving

Cox (1)	6	6	1	6	1	14	HI	horse RBC	A/Vietnam/1203/2004	5	not achieving
Cox (2)	6	6	1	6	1	14	HI	horse RBC	A/Vietnam/1203/2004	45	achieving
Cox (3)	6	6	1	6	1	14	HI	horse RBC	A/Vietnam/1203/2004	130	achieving
Cox (4)	6	6	1	6	1	14	HI	horse RBC	A/Vietnam/1203/2004	97	achieving
Major (1)	8	7	4	5	0.71	14	HI	NA	A/Indonesia/05/2005	10	not achieving
Major (2)	8	7	4	7	1	14	HI	NA	A/Indonesia/05/2005	10	not achieving
Liu (1)	6	6	0	6	1	14	HI	horse RBC	A/bar-headed goose/Qinghai/1A/2005	140	achieving
Liu (2)	6	6	0	3	0.5	14	HI	horse RBC	A/bar-headed goose/Qinghai/1A/2005	5	not achieving
Hatta (1)	9	9	4	8	0.89	14	HI	turkey RBC	A/Vietnam/1203/2004	<10	not achieving
Hatta (2)	9	9	4	8	0.89	14	HI	turkey RBC	A/Vietnam/1203/2004	<10	not achieving
Hatta (3)	9	9	4	9	1	14	HI	horse RBC	A/Vietnam/1203/2004	47	achieving
Hatta (4)	9	9	4	8	0.89	14	HI	horse RBC	A/Vietnam/1203/2004	47	achieving
Wong (1)	4	4	0	3	0.75	14	HI	horse RBC	A/Vietnam/1203/2004	5	not achieving
Wong (2)	4	4	0	4	1	14	HI	horse RBC	A/Vietnam/1203/2004	28	not achieving
Wong (3)	4	4	0	4	1	14	HI	horse RBC	A/Vietnam/1203/2004	761	achieving
Smith (1)	6	8	2	8	1	14	HI	horse RBC	A/Indonesia/05/2005	6	not achieving
Smith (2)	6	8	2	7	0.88	14	HI	horse RBC	A/Indonesia/05/2005	5	not achieving
Smith (3)	8	8	1	8	1	14	HI	horse RBC	A/Vietnam/1203/2004	83	achieving
Smith (4)	8	8	1	8	1	14	HI	horse RBC	A/Indonesia/05/2005	13	not achieving
Wang (1)	8	8	1	8	1	14	HI	MDCK	A/Indonesia/05/2005	704	achieving

Wang (2)	8	8	1	6	0.75	14	HI	MDCK	A/Indonesia/05/2005	10	not achieving
Schön (1)	2	4	1	4	1	14	HI	chicken RBC	A/Cygnus cygnus/Germany/R65/2006	102	achieving
Furey	4	4	0	4	1	14	MN	MDCK	A/Astrakhan/3212/2020	>1024	achieving
Hatta (1)	3	3	0	2	0.67	14	HI	horse RBC	A/American wigeon/South Carolina/22–000345-001/2021	1545	achieving
Hatta (2)	3	3	0	3	1	14	HI	horse RBC	A/American wigeon/South Carolina/22–000345-001/2021	222	achieving
Hatta (3)	3	3	0	3	1	14	HI	horse RBC	A/American wigeon/South Carolina/22–000345-001/2021	1654	achieving
Hatta (4)	3	3	0	3	1	14	HI	horse RBC	A/American wigeon/South Carolina/22–000345-001/2021	553	achieving
Hatta (5)	3	2	0	2	1	14	HI	horse RBC	A/American wigeon/South	301	achieving

									Carolina/22–000345-001/2021		
Hatta (6)	10	9	0	9	1	14	HI	horse RBC	A/American wigeon/South Carolina/22–000345-001/2021	320	achieving
Hatta (7)	10	10	0	9	0.9	14	HI	horse RBC	A/American wigeon/South Carolina/22–000345-001/2021	424	achieving
Hatta (8)	10	10	0	10	1	14	HI	horse RBC	A/American wigeon/South Carolina/22–000345-001/2021	60	achieving

Supplementary Table 4. PRISMA checklist.

Section and Topic	Item #	Checklist item	Location where item is reported
**TITLE**			
Title	1	Identify the report as a systematic review.	Title
**ABSTRACT**			
Abstract	2	See the PRISMA 2020 for Abstracts checklist.	Abstract, paragraphs 1–4
**INTRODUCTION**			
Rationale	3	Describe the rationale for the review in the context of existing knowledge.	Introduction, paragraphs 1-4
Objectives	4	Provide an explicit statement of the objective(s) or question(s) the review addresses.	Introduction, paragraphs 3-4
**METHODS**			
Eligibility criteria	5	Specify the inclusion and exclusion criteria for the review and how studies were grouped for the syntheses.	Method, paragraph 2-3
Information	6	Specify all databases, registers, websites, organisations, reference lists and other sources searched or consulted to identify	Method, paragraph 1;

Section and Topic	Item #	Checklist item	Location where item is reported
sources		studies. Specify the date when each source was last searched or consulted.	Supplementary Table 1
Search strategy	7	Present the full search strategies for all databases, registers and websites, including any filters and limits used.	Method, paragraphs 1; Supplementary Table 1
Selection process	8	Specify the methods used to decide whether a study met the inclusion criteria of the review, including how many reviewers screened each record and each report retrieved, whether they worked independently, and if applicable, details of automation tools used in the process.	Method, paragraph 3
Data collection process	9	Specify the methods used to collect data from reports, including how many reviewers collected data from each report, whether they worked independently, any processes for obtaining or confirming data from study investigators, and if applicable, details of automation tools used in the process.	Method, paragraph 3-5
Data items	10a	List and define all outcomes for which data were sought. Specify whether all results that were compatible with each outcome domain in each study were sought (e.g. for all measures, time points, analyses), and if not, the methods used to decide which results to collect.	Method, paragraphs 5-6
	10b	List and define all other variables for which data were sought (e.g. participant and intervention characteristics, funding sources). Describe any assumptions made about any missing or unclear information.	Method, paragraphs 5-6

Section and Topic	Item #	Checklist item	Location where item is reported
Study risk of bias assessment	11	Specify the methods used to assess risk of bias in the included studies, including details of the tool(s) used, how many reviewers assessed each study and whether they worked independently, and if applicable, details of automation tools used in the process.	Method, paragraph 3 and 8; Supplementary Figure 15-16
Effect measures	12	Specify for each outcome the effect measure(s) (e.g. risk ratio, mean difference) used in the synthesis or presentation of results.	Method, paragraphs 6-7
Synthesis methods	13a	Describe the processes used to decide which studies were eligible for each synthesis (e.g. tabulating the study intervention characteristics and comparing against the planned groups for each synthesis (item #5)).	Method, paragraph 6; Figure 1; Supplementary Table 3
	13b	Describe any methods required to prepare the data for presentation or synthesis, such as handling of missing summary statistics, or data conversions.	Method, paragraph 3-4; Supplementary Table 3
	13c	Describe any methods used to tabulate or visually display results of individual studies and syntheses.	Method, paragraph 6; Results

Section and Topic	Item #	Checklist item	Location where item is reported
			paragraphs 4–7; Figures 2–5; Supplementary Table 3
	13d	Describe any methods used to synthesize results and provide a rationale for the choice(s). If meta-analysis was performed, describe the model(s), method(s) to identify the presence and extent of statistical heterogeneity, and software package(s) used.	Method, paragraph 6, 7, and 12.
	13e	Describe any methods used to explore possible causes of heterogeneity among study results (e.g. subgroup analysis, meta-regression).	Methods, paragraph 6-7; Results paragraphs 4–7; Figures 3–5; Supplementary Table 3
	13f	Describe any sensitivity analyses conducted to assess robustness of the synthesized results.	Method, paragraph 9-11; Supplementary Figure 6–14

Section and Topic	Item #	Checklist item	Location where item is reported
Reporting bias assessment	14	Describe any methods used to assess risk of bias due to missing results in a synthesis (arising from reporting biases).	Method, paragraph 9-11; Results paragraph 10; Supplementary Figures 13, 14, 17 and 18.
Certainty assessment	15	Describe any methods used to assess certainty (or confidence) in the body of evidence for an outcome.	Method, paragraph 9-11; Results paragraphs 4-9; Supplementary Figure 19
**RESULTS**			
Study selection	16a	Describe the results of the search and selection process, from the number of records identified in the search to the number of studies included in the review, ideally using a flow diagram.	Result, paragraph 1; Figure 1
	16b	Cite studies that might appear to meet the inclusion criteria, but which were excluded, and explain why they were excluded.	Supplementary

Section and Topic	Item #	Checklist item	Location where item is reported
			Table 2
Study characteristics	17	Cite each included study and present its characteristics.	Supplementary Table 3
Risk of bias in studies	18	Present assessments of risk of bias for each included study.	Result, paragraph 10; Supplementary Figure 13-14
Results of individual studies	19	For all outcomes, present, for each study: (a) summary statistics for each group (where appropriate) and (b) an effect estimate and its precision (e.g. confidence/credible interval), ideally using structured tables or plots.	Result, paragraphs 4-8; Figure 2-4, and 6; Supplementary Table 3
Results of syntheses	20a	For each synthesis, briefly summarise the characteristics and risk of bias among contributing studies.	Method, paragraph 1; Result, paragraphs 1-3, and 10; Supplementary

Section and Topic	Item #	Checklist item	Location where item is reported
			Table 3; ; Supplementary Figure 15-16
	20b	Present results of all statistical syntheses conducted. If meta-analysis was done, present for each the summary estimate and its precision (e.g. confidence/credible interval) and measures of statistical heterogeneity. If comparing groups, describe the direction of the effect.	Result, paragraphs 4-12; Figure 2-7; Supplementary Figure 1-19
	20c	Present results of all investigations of possible causes of heterogeneity among study results.	Result, paragraphs 9-11; Supplementary Figure 6-14, 17, and 18.
	20d	Present results of all sensitivity analyses conducted to assess the robustness of the synthesized results.	Result, paragraph 10; Supplementary Figure 13-14

Section and Topic	Item #	Checklist item	Location where item is reported
Reporting biases	21	Present assessments of risk of bias due to missing results (arising from reporting biases) for each synthesis assessed.	Method, paragraph 12; Result, paragraph 11; Supplementary Figure 17-18
Certainty of evidence	22	Present assessments of certainty (or confidence) in the body of evidence for each outcome assessed.	Method, paragraph 11,; Result, paragraph 4 and 6; Supplementary Figure 19
**DISCUSSION**			
Discussion	23a	Provide a general interpretation of the results in the context of other evidence.	Discussion, paragraphs 1
	23b	Discuss any limitations of the evidence included in the review.	Discussion, paragraph 8-10

Section and Topic	Item #	Checklist item	Location where item is reported
	23c	Discuss any limitations of the review processes used.	Method, paragraph 11; Discussion, paragraph 10
	23d	Discuss implications of the results for practice, policy, and future research.	Discussion, paragraph 6, 7, and 10.
**OTHER INFORMATION**			
Registration and protocol	24a	Provide registration information for the review, including register name and registration number, or state that the review was not registered.	Method, paragraph 1
	24b	Indicate where the review protocol can be accessed, or state that a protocol was not prepared.	Method, paragraph 1
	24c	Describe and explain any amendments to information provided at registration or in the protocol.	NA
Support	25	Describe sources of financial or non-financial support for the review, and the role of the funders or sponsors in the review.	Summary, funding

Section and Topic	Item #	Checklist item	Location where item is reported
Competing interests	26	Declare any competing interests of review authors.	Declaration of interests
Availability of data, code and other materials	27	Report which of the following are publicly available and where they can be found: template data collection forms; data extracted from included studies; data used for all analyses; analytic code; any other materials used in the review.	Data sharing

From: Page MJ, McKenzie JE, Bossuyt PM, Boutron I, Hoffmann TC, Mulrow CD, et al. The PRISMA 2020 statement: an updated guideline for reporting systematic reviews. BMJ 2021;372:n71. doi: 10.1136/bmj.n71. This work is licensed under CC BY 4.0. To view a copy of this license, visit https://creativecommons.org/licenses/by/4.0/

Supplementary Figure 1. Vaccine efficacy in all H5N1 vaccine trials versus N1-containing seasonal influenza vaccine trials.

Study	Vaccinated Events	Vaccinated Total	Control Events	Control Total	VE	95%-CI	Weight
**Vaccine = seasonal influenza vaccine with N1**							
Haynes et al (2009) (1)	0	7	6	7	92.31	[-13.60; 99.48]	0.4%
Perrone et al (2009) (1)	0	6	6	6	92.31	[-10.31; 99.46]	0.4%
Perrone et al (2009) (2)	3	6	6	6	46.15	[-9.71; 73.57]	1.5%
Price et al (2009) (1)	1	6	6	6	76.92	[6.08; 94.33]	1.0%
Price et al (2009) (2)	0	6	6	6	92.31	[-10.31; 99.46]	0.4%
Rockman et al (2013) (1)	2	4	4	4	44.44	[-26.95; 75.69]	1.4%
Rockman et al (2013) (11)	0	4	4	4	88.89	[-51.60; 99.19]	0.4%
Rockman et al (2013) (12)	0	4	4	4	88.89	[-51.60; 99.19]	0.4%
Rockman et al (2013) (14)	0	4	4	4	88.89	[-51.60; 99.19]	0.4%
Rockman et al (2013) (2)	0	4	4	4	88.89	[-51.60; 99.19]	0.4%
Rockman et al (2013) (3)	0	4	4	4	88.89	[-51.60; 99.19]	0.4%
Rockman et al (2013) (4)	0	3	4	4	85.71	[-85.95; 98.90]	0.5%
Rockman et al (2013) (6)	0	4	4	4	88.89	[-51.60; 99.19]	0.4%
Rockman et al (2013) (7)	0	4	4	4	88.89	[-51.60; 99.19]	0.4%
Rockman et al (2013) (8)	5	8	4	4	35.29	[-6.31; 60.62]	1.7%
Park et al (2014) (1)	0	12	12	12	96.00	[39.53; 99.74]	0.4%
Park et al (2014) (3)	12	12	12	12	0.00	[-17.35; 14.79]	1.9%
Hatta et al (2017) (1)	1	9	5	9	80.00	[-38.91; 97.12]	0.7%
Hatta et al (2017) (2)	1	9	5	9	80.00	[-38.91; 97.12]	0.7%
**Random effects model**		**116**		**113**	**72.78**	**[53.99; 83.90]**	**14.2%**
Heterogeneity: $I^2 = 62.1\%$, $\tau^2 = 0.5272$, $p = 0.0002$							
**Vaccine = H5N1 vaccine**							
Hoffmann et al (2005) (1)	0	3	3	3	85.71	[-85.95; 98.90]	0.5%
Hoffmann et al (2005) (2)	0	3	3	3	85.71	[-85.95; 98.90]	0.5%
Govorkova et al (2006) (1)	0	3	3	3	85.71	[-85.95; 98.90]	0.5%
Govorkova et al (2006) (2)	0	3	3	3	85.71	[-85.95; 98.90]	0.5%
Govorkova et al (2006) (3)	0	3	3	3	85.71	[-85.95; 98.90]	0.5%
Baras et al (2008) (1)	6	6	6	6	0.00	[-36.87; 26.94]	1.8%
Baras et al (2008) (2)	1	6	6	6	76.92	[6.08; 94.33]	1.0%
Baras et al (2008) (4)	0	5	6	6	90.91	[-27.75; 99.35]	0.4%
Baras et al (2008) (5)	0	6	6	6	92.31	[-10.31; 99.46]	0.4%
Lalor et al (2008) (1)	5	6	6	6	15.38	[-17.44; 39.03]	1.8%
Lalor et al (2008) (4)	0	6	6	6	92.31	[-10.31; 99.46]	0.4%
Mahmood et al (2008) (1)	0	4	3	4	85.71	[-104.14; 99.00]	0.4%
Mahmood et al (2008) (9)	0	4	4	4	88.89	[-51.60; 99.19]	0.4%
Forrest et al (2009) (3)	0	5	5	5	90.91	[-27.75; 99.35]	0.4%
Forrest et al (2009) (6)	0	5	5	5	90.91	[-27.75; 99.35]	0.4%
Forrest et al (2009) (7)	0	5	5	5	90.91	[-27.75; 99.35]	0.4%
Forrest et al (2009) (8)	0	5	5	5	90.91	[-27.75; 99.35]	0.4%
Middleton et al (2009) (1)	2	4	3	4	33.33	[-106.70; 78.50]	1.2%
Middleton et al (2009) (10)	0	4	4	4	88.89	[-51.60; 99.19]	0.4%
Middleton et al (2009) (11)	0	4	4	4	88.89	[-51.60; 99.19]	0.4%
Middleton et al (2009) (12)	0	4	3	4	85.71	[-104.14; 99.00]	0.4%
Middleton et al (2009) (13)	0	4	3	4	85.71	[-104.14; 99.00]	0.4%
Middleton et al (2009) (14)	0	4	3	4	85.71	[-104.14; 99.00]	0.4%
Middleton et al (2009) (15)	0	4	3	4	85.71	[-104.14; 99.00]	0.4%
Middleton et al (2009) (16)	1	4	3	4	66.67	[-99.49; 94.43]	0.8%
Middleton et al (2009) (17)	1	4	3	4	66.67	[-99.49; 94.43]	0.8%
Middleton et al (2009) (2)	3	4	3	4	0.00	[-122.59; 55.07]	1.5%
Middleton et al (2009) (3)	2	4	3	4	33.33	[-106.70; 78.50]	1.2%
Middleton et al (2009) (5)	0	4	4	4	88.89	[-51.60; 99.19]	0.4%
Middleton et al (2009) (7)	0	4	4	4	88.89	[-51.60; 99.19]	0.4%
Middleton et al (2009) (8)	0	4	4	4	88.89	[-51.60; 99.19]	0.4%
Middleton et al (2009) (9)	0	4	4	4	88.89	[-51.60; 99.19]	0.4%
Song et al (2009) (1)	0	4	3	4	85.71	[-104.14; 99.00]	0.4%
Rao et al (2010) (3)	4	4	5	5	0.00	[-52.22; 34.31]	1.7%
Rao et al (2010) (4)	5	5	5	5	0.00	[-45.32; 31.19]	1.8%
Rao et al (2010) (5)	4	4	5	5	0.00	[-52.22; 34.31]	1.7%
Gustin et al (2011) (3)	0	6	3	6	85.71	[-124.93; 99.09]	0.4%
Layton et al (2011) (11)	14	14	14	14	0.00	[-14.75; 12.85]	1.9%
Layton et al (2011) (5)	0	6	3	3	92.31	[-10.31; 99.46]	0.4%
Layton et al (2011) (6)	5	14	14	14	62.07	[26.73; 80.36]	1.6%
Layton et al (2011) (7)	8	14	14	14	41.38	[9.66; 61.96]	1.7%
Layton et al (2011) (8)	8	14	14	14	41.38	[9.66; 61.96]	1.7%
Liu et al (2012) (1)	0	6	5	6	90.91	[-33.01; 99.38]	0.4%
Liu et al (2012) (2)	0	6	5	6	90.91	[-33.01; 99.38]	0.4%
Liu et al (2012) (3)	0	6	5	6	90.91	[-33.01; 99.38]	0.4%
Liu et al (2012) (4)	0	6	5	6	90.91	[-33.01; 99.38]	0.4%
Vela et al (2012) (1)	4	8	8	8	47.06	[0.22; 71.91]	1.6%
Vela et al (2012) (2)	6	8	8	8	23.53	[-11.03; 47.33]	1.8%
Vela et al (2012) (3)	0	8	8	8	94.12	[13.43; 99.60]	0.4%
Vela et al (2012) (4)	0	8	8	8	94.12	[13.43; 99.60]	0.4%
Verma et al (2012) (1)	4	6	6	6	30.77	[-15.58; 58.53]	1.7%
Verma et al (2012) (3)	0	6	6	6	92.31	[-10.31; 99.46]	0.4%
Verma et al (2012) (4)	5	6	6	6	15.38	[-17.44; 39.03]	1.8%
Verma et al (2012) (6)	0	6	6	6	92.31	[-10.31; 99.46]	0.4%
Mann et al (2014) (1)	2	6	5	6	60.00	[-31.07; 87.79]	1.1%
Mann et al (2014) (2)	0	6	5	6	90.91	[-33.01; 99.38]	0.4%
Baz et al (2015) (1)	0	4	4	4	88.89	[-51.60; 99.19]	0.4%
Cox et al (2015) (1)	0	6	5	6	90.91	[-33.01; 99.38]	0.4%
Major et al (2015) (1)	2	7	4	8	42.86	[-122.85; 85.35]	1.0%
Major et al (2015) (2)	0	7	4	8	87.41	[-97.34; 99.20]	0.4%
Liu et al (2016) (2)	3	6	6	6	46.15	[-9.71; 73.57]	1.5%
Wong et al (2017) (1)	1	4	4	4	66.67	[-23.13; 90.98]	1.0%
Wong et al (2017) (2)	0	4	4	4	88.89	[-51.60; 99.19]	0.4%
Smith et al (2019) (1)	0	8	4	6	91.50	[-31.25; 99.45]	0.4%
Smith et al (2019) (2)	1	8	4	6	81.25	[-27.73; 97.25]	0.7%
Smith et al (2019) (4)	0	8	7	8	93.33	[0.78; 99.55]	0.4%
Wang et al (2019) (2)	2	8	7	8	71.43	[2.40; 91.64]	1.1%
Hoffmann et al (2005) (3)	0	3	3	3	85.71	[-85.95; 98.90]	0.5%
Baras et al (2008) (3)	0	6	6	6	92.31	[-10.31; 99.46]	0.4%
Lalor et al (2008) (2)	0	6	6	6	92.31	[-10.31; 99.46]	0.4%
Lalor et al (2008) (3)	0	6	6	6	92.31	[-10.31; 99.46]	0.4%
Mahmood et al (2008) (10)	0	4	4	4	88.89	[-51.60; 99.19]	0.4%
Mahmood et al (2008) (2)	0	4	3	4	85.71	[-104.14; 99.00]	0.4%
Mahmood et al (2008) (3)	0	4	3	4	85.71	[-104.14; 99.00]	0.4%
Mahmood et al (2008) (4)	0	4	3	4	85.71	[-104.14; 99.00]	0.4%
Mahmood et al (2008) (5)	0	4	3	4	85.71	[-104.14; 99.00]	0.4%
Mahmood et al (2008) (6)	0	4	4	4	88.89	[-51.60; 99.19]	0.4%
Mahmood et al (2008) (7)	0	4	4	4	88.89	[-51.60; 99.19]	0.4%
Mahmood et al (2008) (8)	0	4	4	4	88.89	[-51.60; 99.19]	0.4%
Forrest et al (2009) (1)	0	5	5	5	90.91	[-27.75; 99.35]	0.4%
Forrest et al (2009) (2)	0	5	5	5	90.91	[-27.75; 99.35]	0.4%
Forrest et al (2009) (4)	0	5	5	5	90.91	[-27.75; 99.35]	0.4%
Forrest et al (2009) (5)	0	5	5	5	90.91	[-27.75; 99.35]	0.4%
Haynes et al (2009) (2)	0	7	6	7	92.31	[-13.60; 99.48]	0.4%
Haynes et al (2009) (3)	0	7	6	7	92.31	[-13.60; 99.48]	0.4%
Middleton et al (2009) (4)	0	4	3	4	85.71	[-104.14; 99.00]	0.4%
Middleton et al (2009) (6)	0	3	4	4	85.71	[-85.95; 98.90]	0.5%
Shoji et al (2009) (1)	0	10	5	10	90.91	[-44.47; 99.43]	0.4%
Shoji et al (2009) (2)	0	10	5	10	90.91	[-44.47; 99.43]	0.4%
Song et al (2009) (2)	0	4	3	4	85.71	[-104.14; 99.00]	0.4%
Song et al (2009) (3)	0	4	3	4	85.71	[-104.14; 99.00]	0.4%
Song et al (2009) (4)	0	4	3	4	85.71	[-104.14; 99.00]	0.4%
Song et al (2009) (5)	0	4	3	4	85.71	[-104.14; 99.00]	0.4%
Song et al (2009) (6)	0	4	3	4	85.71	[-104.14; 99.00]	0.4%
Rao et al (2010) (1)	0	4	4	4	88.89	[-51.60; 99.19]	0.4%
Rao et al (2010) (2)	0	4	4	4	88.89	[-51.60; 99.19]	0.4%
Gustin et al (2011) (1)	0	6	6	6	92.31	[-10.31; 99.46]	0.4%
Gustin et al (2011) (2)	0	6	6	6	92.31	[-10.31; 99.46]	0.4%
Gustin et al (2011) (4)	0	5	3	6	83.12	[-160.68; 98.91]	0.4%
Layton et al (2011) (1)	0	7	3	3	93.33	[2.98; 99.54]	0.4%
Layton et al (2011) (10)	1	14	14	14	89.66	[52.92; 97.73]	0.9%
Layton et al (2011) (2)	0	7	3	3	93.33	[2.98; 99.54]	0.4%
Layton et al (2011) (3)	0	7	3	3	93.33	[2.98; 99.54]	0.4%
Layton et al (2011) (4)	0	7	3	3	93.33	[2.98; 99.54]	0.4%
Layton et al (2011) (9)	1	14	14	14	89.66	[52.92; 97.73]	0.9%
Ducatez et al (2012) (1)	0	4	4	4	88.89	[-51.60; 99.19]	0.4%
Ducatez et al (2012) (2)	0	4	4	4	88.89	[-51.60; 99.19]	0.4%
Ducatez et al (2012) (3)	0	4	4	4	88.89	[-51.60; 99.19]	0.4%
Ducatez et al (2012) (4)	0	4	4	4	88.89	[-51.60; 99.19]	0.4%
Scallan et al (2012) (1)	0	8	3	4	92.44	[-16.43; 99.51]	0.4%
Scallan et al (2012) (2)	2	8	3	4	66.67	[-25.64; 91.16]	1.0%
Verma et al (2012) (2)	0	6	6	6	92.31	[-10.31; 99.46]	0.4%
Verma et al (2012) (5)	0	6	6	6	92.31	[-10.31; 99.46]	0.4%
Mann et al (2014) (3)	0	6	5	6	90.91	[-33.01; 99.38]	0.4%
Cox et al (2015) (2)	0	6	5	6	90.91	[-33.01; 99.38]	0.4%
Cox et al (2015) (3)	0	6	5	6	90.91	[-33.01; 99.38]	0.4%
Cox et al (2015) (4)	0	6	5	6	90.91	[-33.01; 99.38]	0.4%
Liu et al (2016) (1)	0	6	6	6	92.31	[-10.31; 99.46]	0.4%
Hatta et al (2017) (3)	0	9	5	9	90.91	[-42.47; 99.42]	0.4%
Hatta et al (2017) (4)	1	9	5	9	80.00	[-38.91; 97.12]	0.7%
Wong et al (2017) (3)	0	4	4	4	88.89	[-51.60; 99.19]	0.4%
Smith et al (2019) (3)	0	8	7	8	93.33	[0.78; 99.55]	0.4%
Wang et al (2019) (1)	0	8	7	8	93.33	[0.78; 99.55]	0.4%
Schön et al (2020) (1)	0	4	1	2	81.48	[-205.27; 98.88]	0.4%
Furey et al (2024) (1)	0	4	4	4	88.89	[-51.60; 99.19]	0.4%
Hatta et al (2024) (1)	1	3	3	3	57.14	[-43.68; 87.22]	1.1%
Hatta et al (2024) (2)	0	3	3	3	85.71	[-85.95; 98.90]	0.5%
Hatta et al (2024) (3)	0	3	3	3	85.71	[-85.95; 98.90]	0.5%
Hatta et al (2024) (4)	0	3	3	3	85.71	[-85.95; 98.90]	0.5%
Hatta et al (2024) (5)	0	2	3	3	80.00	[-138.63; 98.32]	0.5%
Hatta et al (2024) (6)	0	9	10	10	94.74	[21.85; 99.65]	0.4%
Hatta et al (2024) (7)	1	10	10	10	85.71	[37.14; 96.75]	0.9%
Hatta et al (2024) (8)	0	10	10	10	95.24	[28.79; 99.68]	0.4%
**Random effects model**		**763**		**739**	**77.45**	**[71.98; 81.85]**	**85.8%**
Heterogeneity: $I^2 = 54.3\%$, $\tau^2 = 0.5615$, $p < 0.0001$							
**Random effects model**		**879**		**852**	**76.80**	**[71.65; 81.02]**	**100.0%**

Heterogeneity: $I^2 = 55.8\%$, $\tau^2 = 0.5532$, $p < 0.0001$

Test for subgroup differences: $\chi^2_1 = 0.42$, df = 1 ($p = 0.5164$)

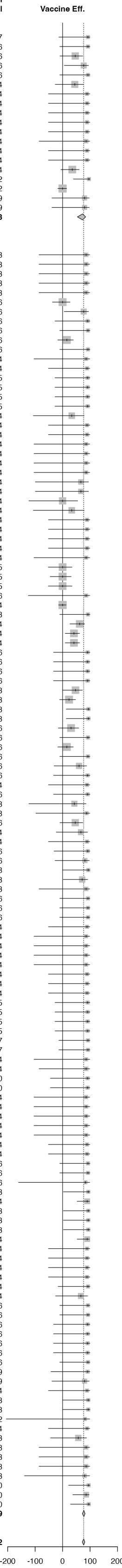

Supplementary Figure 2. Vaccine efficacy in H5N1 vaccine trials with seroprotection versus H5N1 vaccine trials without seroprotection.

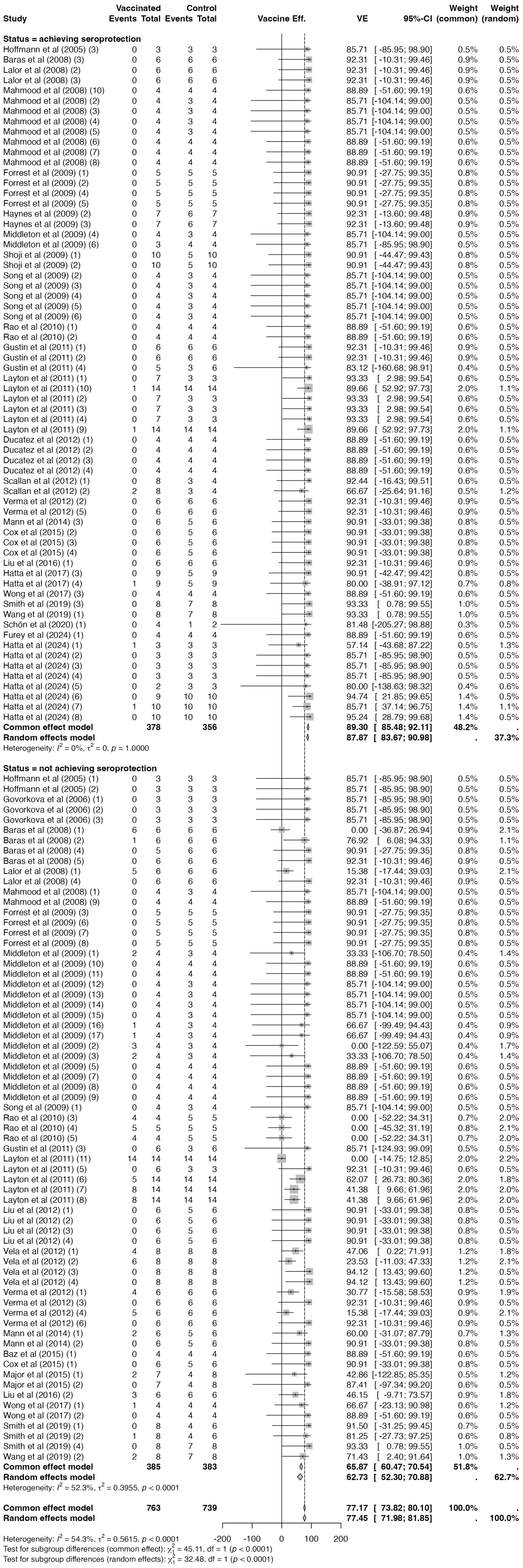

Study	Vaccinated Events	Vaccinated Total	Control Events	Control Total	VE	95%-CI	Weight (common)	Weight (random)
**Status = achieving seroprotection**								
Hoffmann et al (2005) (3)	0	3	3	3	85.71	[-85.95; 98.90]	0.5%	0.5%
Baras et al (2008) (3)	0	6	6	6	92.31	[-10.31; 99.46]	0.9%	0.5%
Lalor et al (2008) (2)	0	6	6	6	92.31	[-10.31; 99.46]	0.9%	0.5%
Lalor et al (2008) (3)	0	6	6	6	92.31	[-10.31; 99.46]	0.9%	0.5%
Mahmood et al (2008) (10)	0	4	4	4	88.89	[-51.60; 99.19]	0.6%	0.5%
Mahmood et al (2008) (2)	0	4	3	4	85.71	[-104.14; 99.00]	0.5%	0.5%
Mahmood et al (2008) (3)	0	4	3	4	85.71	[-104.14; 99.00]	0.5%	0.5%
Mahmood et al (2008) (4)	0	4	3	4	85.71	[-104.14; 99.00]	0.5%	0.5%
Mahmood et al (2008) (5)	0	4	3	4	85.71	[-104.14; 99.00]	0.5%	0.5%
Mahmood et al (2008) (6)	0	4	4	4	88.89	[-51.60; 99.19]	0.6%	0.5%
Mahmood et al (2008) (7)	0	4	4	4	88.89	[-51.60; 99.19]	0.6%	0.5%
Mahmood et al (2008) (8)	0	4	4	4	88.89	[-51.60; 99.19]	0.6%	0.5%
Forrest et al (2009) (1)	0	5	5	5	90.91	[-27.75; 99.35]	0.8%	0.5%
Forrest et al (2009) (2)	0	5	5	5	90.91	[-27.75; 99.35]	0.8%	0.5%
Forrest et al (2009) (4)	0	5	5	5	90.91	[-27.75; 99.35]	0.8%	0.5%
Forrest et al (2009) (5)	0	5	5	5	90.91	[-27.75; 99.35]	0.8%	0.5%
Haynes et al (2009) (2)	0	7	6	7	92.31	[-13.60; 99.48]	0.9%	0.5%
Haynes et al (2009) (3)	0	7	6	7	92.31	[-13.60; 99.48]	0.9%	0.5%
Middleton et al (2009) (4)	0	4	3	4	85.71	[-104.14; 99.00]	0.5%	0.5%
Middleton et al (2009) (6)	0	3	4	4	85.71	[-85.95; 98.90]	0.5%	0.5%
Shoji et al (2009) (1)	0	10	5	10	90.91	[-44.47; 99.43]	0.8%	0.5%
Shoji et al (2009) (2)	0	10	5	10	90.91	[-44.47; 99.43]	0.8%	0.5%
Song et al (2009) (2)	0	4	3	4	85.71	[-104.14; 99.00]	0.5%	0.5%
Song et al (2009) (3)	0	4	3	4	85.71	[-104.14; 99.00]	0.5%	0.5%
Song et al (2009) (4)	0	4	3	4	85.71	[-104.14; 99.00]	0.5%	0.5%
Song et al (2009) (5)	0	4	3	4	85.71	[-104.14; 99.00]	0.5%	0.5%
Song et al (2009) (6)	0	4	3	4	85.71	[-104.14; 99.00]	0.5%	0.5%
Rao et al (2010) (1)	0	4	4	4	88.89	[-51.60; 99.19]	0.6%	0.5%
Rao et al (2010) (2)	0	4	4	4	88.89	[-51.60; 99.19]	0.6%	0.5%
Gustin et al (2011) (1)	0	6	6	6	92.31	[-10.31; 99.46]	0.9%	0.5%
Gustin et al (2011) (2)	0	6	6	6	92.31	[-10.31; 99.46]	0.9%	0.5%
Gustin et al (2011) (4)	0	5	3	6	83.12	[-160.68; 98.91]	0.4%	0.5%
Layton et al (2011) (1)	0	7	3	3	93.33	[2.98; 99.54]	0.6%	0.5%
Layton et al (2011) (10)	1	14	14	14	89.66	[52.92; 97.73]	2.0%	1.1%
Layton et al (2011) (2)	0	7	3	3	93.33	[2.98; 99.54]	0.6%	0.5%
Layton et al (2011) (3)	0	7	3	3	93.33	[2.98; 99.54]	0.6%	0.5%
Layton et al (2011) (4)	0	7	3	3	93.33	[2.98; 99.54]	0.6%	0.5%
Layton et al (2011) (9)	1	14	14	14	89.66	[52.92; 97.73]	2.0%	1.1%
Ducatez et al (2012) (1)	0	4	4	4	88.89	[-51.60; 99.19]	0.6%	0.5%
Ducatez et al (2012) (2)	0	4	4	4	88.89	[-51.60; 99.19]	0.6%	0.5%
Ducatez et al (2012) (3)	0	4	4	4	88.89	[-51.60; 99.19]	0.6%	0.5%
Ducatez et al (2012) (4)	0	4	4	4	88.89	[-51.60; 99.19]	0.6%	0.5%
Scallan et al (2012) (1)	0	8	3	4	92.44	[-16.43; 99.51]	0.6%	0.5%
Scallan et al (2012) (2)	2	8	3	4	66.67	[-25.64; 91.16]	0.5%	1.2%
Verma et al (2012) (2)	0	6	6	6	92.31	[-10.31; 99.46]	0.9%	0.5%
Verma et al (2012) (5)	0	6	6	6	92.31	[-10.31; 99.46]	0.9%	0.5%
Mann et al (2014) (3)	0	6	5	6	90.91	[-33.01; 99.38]	0.8%	0.5%
Cox et al (2015) (2)	0	6	5	6	90.91	[-33.01; 99.38]	0.8%	0.5%
Cox et al (2015) (3)	0	6	5	6	90.91	[-33.01; 99.38]	0.8%	0.5%
Cox et al (2015) (4)	0	6	5	6	90.91	[-33.01; 99.38]	0.8%	0.5%
Liu et al (2016) (1)	0	6	6	6	92.31	[-10.31; 99.46]	0.9%	0.5%
Hatta et al (2017) (3)	0	9	5	9	90.91	[-42.47; 99.42]	0.8%	0.5%
Hatta et al (2017) (4)	1	9	5	9	80.00	[-38.91; 97.12]	0.7%	0.8%
Wong et al (2017) (3)	0	4	4	4	88.89	[-51.60; 99.19]	0.6%	0.5%
Smith et al (2019) (3)	0	8	7	8	93.33	[0.78; 99.55]	1.0%	0.5%
Wang et al (2019) (1)	0	8	7	8	93.33	[0.78; 99.55]	1.0%	0.5%
Schön et al (2020) (1)	0	4	1	2	81.48	[-205.27; 98.88]	0.3%	0.5%
Furey et al (2024) (1)	0	4	4	4	88.89	[-51.60; 99.19]	0.6%	0.5%
Hatta et al (2024) (1)	1	3	3	3	57.14	[-43.68; 87.22]	0.5%	1.3%
Hatta et al (2024) (2)	0	3	3	3	85.71	[-85.95; 98.90]	0.5%	0.5%
Hatta et al (2024) (3)	0	3	3	3	85.71	[-85.95; 98.90]	0.5%	0.5%
Hatta et al (2024) (4)	0	3	3	3	85.71	[-85.95; 98.90]	0.5%	0.5%
Hatta et al (2024) (5)	0	2	3	3	80.00	[-138.63; 98.32]	0.4%	0.6%
Hatta et al (2024) (6)	0	9	10	10	94.74	[21.85; 99.65]	1.4%	0.5%
Hatta et al (2024) (7)	1	10	10	10	85.71	[37.14; 96.75]	1.4%	1.1%
Hatta et al (2024) (8)	0	10	10	10	95.24	[28.79; 99.68]	1.4%	0.5%
**Common effect model**		**378**		**356**	**89.30**	**[85.48; 92.11]**	**48.2%**	**.**
**Random effects model**					**87.87**	**[83.67; 90.98]**	**.**	**37.3%**
Heterogeneity: $I^2 = 0\%$, $\tau^2 = 0$, $p = 1.0000$								
**Status = not achieving seroprotection**								
Hoffmann et al (2005) (1)	0	3	3	3	85.71	[-85.95; 98.90]	0.5%	0.5%
Hoffmann et al (2005) (2)	0	3	3	3	85.71	[-85.95; 98.90]	0.5%	0.5%
Govorkova et al (2006) (1)	0	3	3	3	85.71	[-85.95; 98.90]	0.5%	0.5%
Govorkova et al (2006) (2)	0	3	3	3	85.71	[-85.95; 98.90]	0.5%	0.5%
Govorkova et al (2006) (3)	0	3	3	3	85.71	[-85.95; 98.90]	0.5%	0.5%
Baras et al (2008) (1)	6	6	6	6	0.00	[-36.87; 26.94]	0.9%	2.1%
Baras et al (2008) (2)	1	6	6	6	76.92	[6.08; 94.33]	0.9%	1.1%
Baras et al (2008) (4)	0	5	6	6	90.91	[-27.75; 99.35]	0.8%	0.5%
Baras et al (2008) (5)	0	6	6	6	92.31	[-10.31; 99.46]	0.9%	0.5%
Lalor et al (2008) (1)	5	6	6	6	15.38	[-17.44; 39.03]	0.9%	2.1%
Lalor et al (2008) (4)	0	6	6	6	92.31	[-10.31; 99.46]	0.9%	0.5%
Mahmood et al (2008) (1)	0	4	3	4	85.71	[-104.14; 99.00]	0.5%	0.5%
Mahmood et al (2008) (9)	0	4	4	4	88.89	[-51.60; 99.19]	0.6%	0.5%
Forrest et al (2009) (3)	0	5	5	5	90.91	[-27.75; 99.35]	0.8%	0.5%
Forrest et al (2009) (6)	0	5	5	5	90.91	[-27.75; 99.35]	0.8%	0.5%
Forrest et al (2009) (7)	0	5	5	5	90.91	[-27.75; 99.35]	0.8%	0.5%
Forrest et al (2009) (8)	0	5	5	5	90.91	[-27.75; 99.35]	0.8%	0.5%
Middleton et al (2009) (1)	2	4	3	4	33.33	[-106.70; 78.50]	0.4%	1.4%
Middleton et al (2009) (10)	0	4	4	4	88.89	[-51.60; 99.19]	0.6%	0.5%
Middleton et al (2009) (11)	0	4	4	4	88.89	[-51.60; 99.19]	0.6%	0.5%
Middleton et al (2009) (12)	0	4	3	4	85.71	[-104.14; 99.00]	0.5%	0.5%
Middleton et al (2009) (13)	0	4	3	4	85.71	[-104.14; 99.00]	0.5%	0.5%
Middleton et al (2009) (14)	0	4	3	4	85.71	[-104.14; 99.00]	0.5%	0.5%
Middleton et al (2009) (15)	0	4	3	4	85.71	[-104.14; 99.00]	0.5%	0.5%
Middleton et al (2009) (16)	1	4	3	4	66.67	[-99.49; 94.43]	0.4%	0.9%
Middleton et al (2009) (17)	1	4	3	4	66.67	[-99.49; 94.43]	0.4%	0.9%
Middleton et al (2009) (2)	3	4	3	4	0.00	[-122.59; 55.07]	0.4%	1.7%
Middleton et al (2009) (3)	2	4	3	4	33.33	[-106.70; 78.50]	0.4%	1.4%
Middleton et al (2009) (5)	0	4	4	4	88.89	[-51.60; 99.19]	0.6%	0.5%
Middleton et al (2009) (7)	0	4	4	4	88.89	[-51.60; 99.19]	0.6%	0.5%
Middleton et al (2009) (8)	0	4	4	4	88.89	[-51.60; 99.19]	0.6%	0.5%
Middleton et al (2009) (9)	0	4	4	4	88.89	[-51.60; 99.19]	0.6%	0.5%
Song et al (2009) (1)	0	4	3	4	85.71	[-104.14; 99.00]	0.5%	0.5%
Rao et al (2010) (3)	4	4	5	5	0.00	[-52.22; 34.31]	0.7%	2.0%
Rao et al (2010) (4)	5	5	5	5	0.00	[-45.32; 31.19]	0.8%	2.1%
Rao et al (2010) (5)	4	4	5	5	0.00	[-52.22; 34.31]	0.7%	2.0%
Gustin et al (2011) (3)	0	6	3	6	85.71	[-124.93; 99.09]	0.5%	0.5%
Layton et al (2011) (11)	14	14	14	14	0.00	[-14.75; 12.85]	2.0%	2.2%
Layton et al (2011) (5)	0	6	3	3	92.31	[-10.31; 99.46]	0.6%	0.5%
Layton et al (2011) (6)	5	14	14	14	62.07	[26.73; 80.36]	2.0%	1.8%
Layton et al (2011) (7)	8	14	14	14	41.38	[9.66; 61.96]	2.0%	2.0%
Layton et al (2011) (8)	8	14	14	14	41.38	[9.66; 61.96]	2.0%	2.0%
Liu et al (2012) (1)	0	6	5	6	90.91	[-33.01; 99.38]	0.8%	0.5%
Liu et al (2012) (2)	0	6	5	6	90.91	[-33.01; 99.38]	0.8%	0.5%
Liu et al (2012) (3)	0	6	5	6	90.91	[-33.01; 99.38]	0.8%	0.5%
Liu et al (2012) (4)	0	6	5	6	90.91	[-33.01; 99.38]	0.8%	0.5%
Vela et al (2012) (1)	4	8	8	8	47.06	[0.22; 71.91]	1.2%	1.8%
Vela et al (2012) (2)	6	8	8	8	23.53	[-11.03; 47.33]	1.2%	2.1%
Vela et al (2012) (3)	0	8	8	8	94.12	[13.43; 99.60]	1.2%	0.5%
Vela et al (2012) (4)	0	8	8	8	94.12	[13.43; 99.60]	1.2%	0.5%
Verma et al (2012) (1)	4	6	6	6	30.77	[-15.58; 58.53]	0.9%	1.9%
Verma et al (2012) (3)	0	6	6	6	92.31	[-10.31; 99.46]	0.9%	0.5%
Verma et al (2012) (4)	5	6	6	6	15.38	[-17.44; 39.03]	0.9%	2.1%
Verma et al (2012) (6)	0	6	6	6	92.31	[-10.31; 99.46]	0.9%	0.5%
Mann et al (2014) (1)	2	6	5	6	60.00	[-31.07; 87.79]	0.7%	1.3%
Mann et al (2014) (2)	0	6	5	6	90.91	[-33.01; 99.38]	0.8%	0.5%
Baz et al (2015) (1)	0	4	4	4	88.89	[-51.60; 99.19]	0.6%	0.5%
Cox et al (2015) (1)	0	6	5	6	90.91	[-33.01; 99.38]	0.8%	0.5%
Major et al (2015) (1)	2	7	4	8	42.86	[-122.85; 85.35]	0.5%	1.2%
Major et al (2015) (2)	0	7	4	8	87.41	[-97.34; 99.20]	0.6%	0.5%
Liu et al (2016) (2)	3	6	6	6	46.15	[-9.71; 73.57]	0.9%	1.8%
Wong et al (2017) (1)	1	4	4	4	66.67	[-23.13; 90.98]	0.6%	1.2%
Wong et al (2017) (2)	0	4	4	4	88.89	[-51.60; 99.19]	0.6%	0.5%
Smith et al (2019) (1)	0	8	4	6	91.50	[-31.25; 99.45]	0.7%	0.5%
Smith et al (2019) (2)	1	8	4	6	81.25	[-27.73; 97.25]	0.6%	0.8%
Smith et al (2019) (4)	0	8	7	8	93.33	[0.78; 99.55]	1.0%	0.5%
Wang et al (2019) (2)	2	8	7	8	71.43	[2.40; 91.64]	1.0%	1.3%
**Common effect model**		**385**		**383**	**65.87**	**[60.47; 70.54]**	**51.8%**	**.**
**Random effects model**					**62.73**	**[52.30; 70.88]**	**.**	**62.7%**
Heterogeneity: $I^2 = 52.3\%$, $\tau^2 = 0.3955$, $p < 0.0001$								
**Common effect model**		**763**		**739**	**77.17**	**[73.82; 80.10]**	**100.0%**	**.**
**Random effects model**					**77.45**	**[71.98; 81.85]**	**.**	**100.0%**

Heterogeneity: $I^2 = 54.3\%$, $\tau^2 = 0.5615$, $p < 0.0001$
Test for subgroup differences (common effect): $\chi^2_1 = 45.11$, df = 1 ($p < 0.0001$)
Test for subgroup differences (random effects): $\chi^2_1 = 32.48$, df = 1 ($p < 0.0001$)

Supplementary Figure 3. Vaccine efficacy in H5N1 vaccine trials with seroprotection: subgroup analysis by booster dose.

Study	Vaccinated Events	Vaccinated Total	Control Events	Control Total	VE	95%-CI	Weight
**booster = 0**							
Hatta et al (2017) (4)	1	9	5	9	80.00	[-38.91; 97.12]	2.3%
**booster = 1**							
Hoffmann et al (2005) (3)	0	3	3	3	85.71	[-85.95; 98.90]	1.3%
Baras et al (2008) (3)	0	6	6	6	92.31	[-10.31; 99.46]	1.2%
Lalor et al (2008) (2)	0	6	6	6	92.31	[-10.31; 99.46]	1.2%
Lalor et al (2008) (3)	0	6	6	6	92.31	[-10.31; 99.46]	1.2%
Mahmood et al (2008) (10)	0	4	4	4	88.89	[-51.60; 99.19]	1.3%
Mahmood et al (2008) (2)	0	4	3	4	85.71	[-104.14; 99.00]	1.2%
Mahmood et al (2008) (3)	0	4	3	4	85.71	[-104.14; 99.00]	1.2%
Mahmood et al (2008) (4)	0	4	3	4	85.71	[-104.14; 99.00]	1.2%
Mahmood et al (2008) (5)	0	4	3	4	85.71	[-104.14; 99.00]	1.2%
Mahmood et al (2008) (6)	0	4	4	4	88.89	[-51.60; 99.19]	1.3%
Mahmood et al (2008) (7)	0	4	4	4	88.89	[-51.60; 99.19]	1.3%
Mahmood et al (2008) (8)	0	4	4	4	88.89	[-51.60; 99.19]	1.3%
Forrest et al (2009) (1)	0	5	5	5	90.91	[-27.75; 99.35]	1.3%
Forrest et al (2009) (2)	0	5	5	5	90.91	[-27.75; 99.35]	1.3%
Forrest et al (2009) (4)	0	5	5	5	90.91	[-27.75; 99.35]	1.3%
Forrest et al (2009) (5)	0	5	5	5	90.91	[-27.75; 99.35]	1.3%
Haynes et al (2009) (2)	0	7	6	7	92.31	[-13.60; 99.48]	1.2%
Haynes et al (2009) (3)	0	7	6	7	92.31	[-13.60; 99.48]	1.2%
Middleton et al (2009) (4)	0	4	3	4	85.71	[-104.14; 99.00]	1.2%
Middleton et al (2009) (6)	0	3	4	4	85.71	[-85.95; 98.90]	1.3%
Song et al (2009) (2)	0	4	3	4	85.71	[-104.14; 99.00]	1.2%
Song et al (2009) (3)	0	4	3	4	85.71	[-104.14; 99.00]	1.2%
Song et al (2009) (4)	0	4	3	4	85.71	[-104.14; 99.00]	1.2%
Song et al (2009) (5)	0	4	3	4	85.71	[-104.14; 99.00]	1.2%
Song et al (2009) (6)	0	4	3	4	85.71	[-104.14; 99.00]	1.2%
Gustin et al (2011) (1)	0	6	6	6	92.31	[-10.31; 99.46]	1.2%
Gustin et al (2011) (2)	0	6	6	6	92.31	[-10.31; 99.46]	1.2%
Gustin et al (2011) (4)	0	5	3	6	83.12	[-160.68; 98.91]	1.2%
Layton et al (2011) (1)	0	7	3	3	93.33	[2.98; 99.54]	1.2%
Layton et al (2011) (10)	1	14	14	14	89.66	[52.92; 97.73]	3.8%
Layton et al (2011) (2)	0	7	3	3	93.33	[2.98; 99.54]	1.2%
Layton et al (2011) (3)	0	7	3	3	93.33	[2.98; 99.54]	1.2%
Layton et al (2011) (4)	0	7	3	3	93.33	[2.98; 99.54]	1.2%
Layton et al (2011) (9)	1	14	14	14	89.66	[52.92; 97.73]	3.8%
Scallan et al (2012) (1)	0	8	3	4	92.44	[-16.43; 99.51]	1.2%
Scallan et al (2012) (2)	2	8	3	4	66.67	[-25.64; 91.16]	5.0%
Verma et al (2012) (2)	0	6	6	6	92.31	[-10.31; 99.46]	1.2%
Verma et al (2012) (5)	0	6	6	6	92.31	[-10.31; 99.46]	1.2%
Mann et al (2014) (3)	0	6	5	6	90.91	[-33.01; 99.38]	1.2%
Cox et al (2015) (2)	0	6	5	6	90.91	[-33.01; 99.38]	1.2%
Cox et al (2015) (3)	0	6	5	6	90.91	[-33.01; 99.38]	1.2%
Cox et al (2015) (4)	0	6	5	6	90.91	[-33.01; 99.38]	1.2%
Liu et al (2016) (1)	0	6	6	6	92.31	[-10.31; 99.46]	1.2%
Hatta et al (2017) (3)	0	9	5	9	90.91	[-42.47; 99.42]	1.2%
Wong et al (2017) (3)	0	4	4	4	88.89	[-51.60; 99.19]	1.3%
Smith et al (2019) (3)	0	8	7	8	93.33	[0.78; 99.55]	1.2%
Schön et al (2020) (1)	0	4	1	2	81.48	[-205.27; 98.88]	1.1%
Furey et al (2024) (1)	0	4	4	4	88.89	[-51.60; 99.19]	1.3%
**Random effects model**		**274**		**250**	**88.98**	**[84.23; 92.30]**	**68.5%**
Heterogeneity: I^2 = 0%, τ^2 = 0, p = 1.0000							
**booster = 2**							
Shoji et al (2009) (1)	0	10	5	10	90.91	[-44.47; 99.43]	1.2%
Shoji et al (2009) (2)	0	10	5	10	90.91	[-44.47; 99.43]	1.2%
Ducatez et al (2012) (1)	0	4	4	4	88.89	[-51.60; 99.19]	1.3%
Ducatez et al (2012) (2)	0	4	4	4	88.89	[-51.60; 99.19]	1.3%
Ducatez et al (2012) (3)	0	4	4	4	88.89	[-51.60; 99.19]	1.3%
Ducatez et al (2012) (4)	0	4	4	4	88.89	[-51.60; 99.19]	1.3%
Wang et al (2019) (1)	0	8	7	8	93.33	[0.78; 99.55]	1.2%
Hatta et al (2024) (1)	1	3	3	3	57.14	[-43.68; 87.22]	6.0%
Hatta et al (2024) (2)	0	3	3	3	85.71	[-85.95; 98.90]	1.3%
Hatta et al (2024) (3)	0	3	3	3	85.71	[-85.95; 98.90]	1.3%
Hatta et al (2024) (4)	0	3	3	3	85.71	[-85.95; 98.90]	1.3%
Hatta et al (2024) (5)	0	2	3	3	80.00	[-138.63; 98.32]	1.4%
Hatta et al (2024) (6)	0	9	10	10	94.74	[21.85; 99.65]	1.2%
Hatta et al (2024) (7)	1	10	10	10	85.71	[37.14; 96.75]	4.0%
Hatta et al (2024) (8)	0	10	10	10	95.24	[28.79; 99.68]	1.2%
**Random effects model**		**87**		**89**	**84.99**	**[73.31; 91.56]**	**26.5%**
Heterogeneity: I^2 = 0%, τ^2 = 0, p = 0.9855							
**booster = 3**							
Rao et al (2010) (1)	0	4	4	4	88.89	[-51.60; 99.19]	1.3%
Rao et al (2010) (2)	0	4	4	4	88.89	[-51.60; 99.19]	1.3%
**Random effects model**		**8**		**8**	**88.89**	**[29.49; 98.25]**	**2.6%**
Heterogeneity: I^2 = 0%, τ^2 = 0, p = 1.0000							
**Random effects model**		**378**		**356**	**87.87**	**[83.67; 90.98]**	**100.0%**
Heterogeneity: I^2 = 0.0%, τ^2 = 0, p = 1.0000							
Test for subgroup differences: χ^2_3 = 1.06, df = 3 (p = 0.7862)							

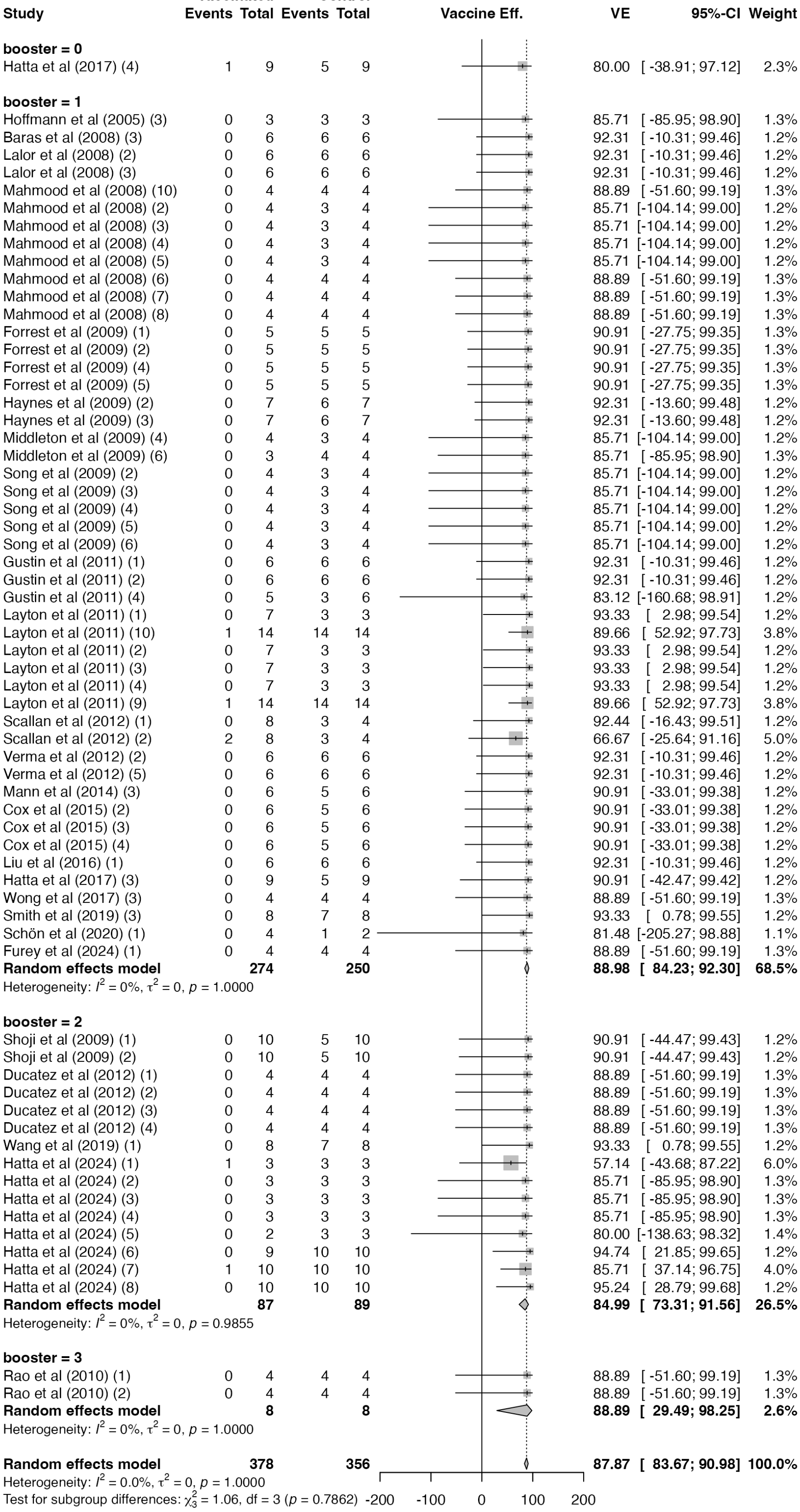

Supplementary Figure 4. Vaccine efficacy in H5N1 vaccine trials with seroprotection versus N1-containing seasonal influenza vaccine trials.

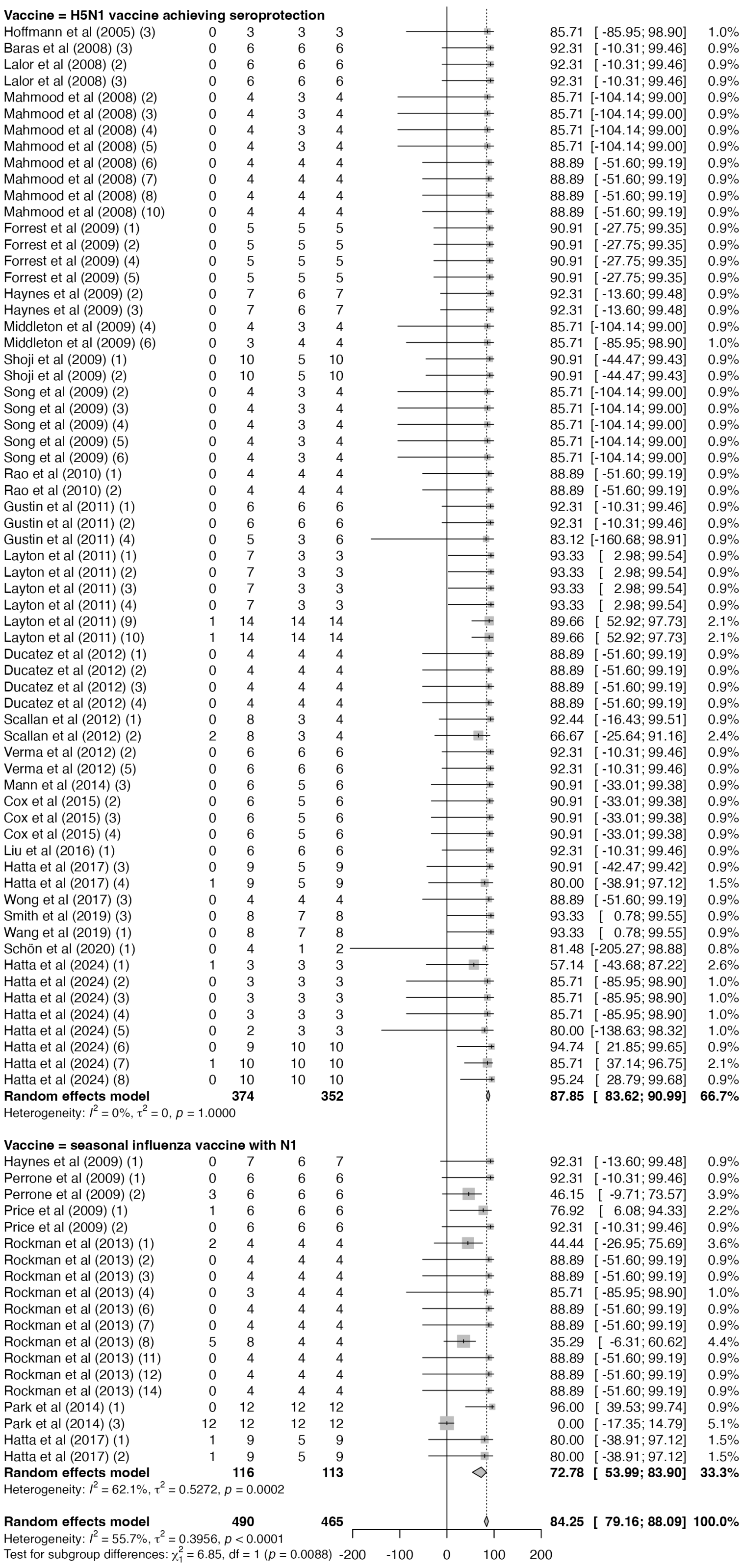

Study	Vaccinated Events	Vaccinated Total	Control Events	Control Total	VE	95%-CI	Weight
**Vaccine = H5N1 vaccine achieving seroprotection**							
Hoffmann et al (2005) (3)	0	3	3	3	85.71	[-85.95; 98.90]	1.0%
Baras et al (2008) (3)	0	6	6	6	92.31	[-10.31; 99.46]	0.9%
Lalor et al (2008) (2)	0	6	6	6	92.31	[-10.31; 99.46]	0.9%
Lalor et al (2008) (3)	0	6	6	6	92.31	[-10.31; 99.46]	0.9%
Mahmood et al (2008) (2)	0	4	3	4	85.71	[-104.14; 99.00]	0.9%
Mahmood et al (2008) (3)	0	4	3	4	85.71	[-104.14; 99.00]	0.9%
Mahmood et al (2008) (4)	0	4	3	4	85.71	[-104.14; 99.00]	0.9%
Mahmood et al (2008) (5)	0	4	3	4	85.71	[-104.14; 99.00]	0.9%
Mahmood et al (2008) (6)	0	4	4	4	88.89	[-51.60; 99.19]	0.9%
Mahmood et al (2008) (7)	0	4	4	4	88.89	[-51.60; 99.19]	0.9%
Mahmood et al (2008) (8)	0	4	4	4	88.89	[-51.60; 99.19]	0.9%
Mahmood et al (2008) (10)	0	4	4	4	88.89	[-51.60; 99.19]	0.9%
Forrest et al (2009) (1)	0	5	5	5	90.91	[-27.75; 99.35]	0.9%
Forrest et al (2009) (2)	0	5	5	5	90.91	[-27.75; 99.35]	0.9%
Forrest et al (2009) (4)	0	5	5	5	90.91	[-27.75; 99.35]	0.9%
Forrest et al (2009) (5)	0	5	5	5	90.91	[-27.75; 99.35]	0.9%
Haynes et al (2009) (2)	0	7	6	7	92.31	[-13.60; 99.48]	0.9%
Haynes et al (2009) (3)	0	7	6	7	92.31	[-13.60; 99.48]	0.9%
Middleton et al (2009) (4)	0	4	3	4	85.71	[-104.14; 99.00]	0.9%
Middleton et al (2009) (6)	0	3	4	4	85.71	[-85.95; 98.90]	1.0%
Shoji et al (2009) (1)	0	10	5	10	90.91	[-44.47; 99.43]	0.9%
Shoji et al (2009) (2)	0	10	5	10	90.91	[-44.47; 99.43]	0.9%
Song et al (2009) (2)	0	4	3	4	85.71	[-104.14; 99.00]	0.9%
Song et al (2009) (3)	0	4	3	4	85.71	[-104.14; 99.00]	0.9%
Song et al (2009) (4)	0	4	3	4	85.71	[-104.14; 99.00]	0.9%
Song et al (2009) (5)	0	4	3	4	85.71	[-104.14; 99.00]	0.9%
Song et al (2009) (6)	0	4	3	4	85.71	[-104.14; 99.00]	0.9%
Rao et al (2010) (1)	0	4	4	4	88.89	[-51.60; 99.19]	0.9%
Rao et al (2010) (2)	0	4	4	4	88.89	[-51.60; 99.19]	0.9%
Gustin et al (2011) (1)	0	6	6	6	92.31	[-10.31; 99.46]	0.9%
Gustin et al (2011) (2)	0	6	6	6	92.31	[-10.31; 99.46]	0.9%
Gustin et al (2011) (4)	0	5	3	6	83.12	[-160.68; 98.91]	0.9%
Layton et al (2011) (1)	0	7	3	3	93.33	[2.98; 99.54]	0.9%
Layton et al (2011) (2)	0	7	3	3	93.33	[2.98; 99.54]	0.9%
Layton et al (2011) (3)	0	7	3	3	93.33	[2.98; 99.54]	0.9%
Layton et al (2011) (4)	0	7	3	3	93.33	[2.98; 99.54]	0.9%
Layton et al (2011) (9)	1	14	14	14	89.66	[52.92; 97.73]	2.1%
Layton et al (2011) (10)	1	14	14	14	89.66	[52.92; 97.73]	2.1%
Ducatez et al (2012) (1)	0	4	4	4	88.89	[-51.60; 99.19]	0.9%
Ducatez et al (2012) (2)	0	4	4	4	88.89	[-51.60; 99.19]	0.9%
Ducatez et al (2012) (3)	0	4	4	4	88.89	[-51.60; 99.19]	0.9%
Ducatez et al (2012) (4)	0	4	4	4	88.89	[-51.60; 99.19]	0.9%
Scallan et al (2012) (1)	0	8	3	4	92.44	[-16.43; 99.51]	0.9%
Scallan et al (2012) (2)	2	8	3	4	66.67	[-25.64; 91.16]	2.4%
Verma et al (2012) (2)	0	6	6	6	92.31	[-10.31; 99.46]	0.9%
Verma et al (2012) (5)	0	6	6	6	92.31	[-10.31; 99.46]	0.9%
Mann et al (2014) (3)	0	6	5	6	90.91	[-33.01; 99.38]	0.9%
Cox et al (2015) (2)	0	6	5	6	90.91	[-33.01; 99.38]	0.9%
Cox et al (2015) (3)	0	6	5	6	90.91	[-33.01; 99.38]	0.9%
Cox et al (2015) (4)	0	6	5	6	90.91	[-33.01; 99.38]	0.9%
Liu et al (2016) (1)	0	6	6	6	92.31	[-10.31; 99.46]	0.9%
Hatta et al (2017) (3)	0	9	5	9	90.91	[-42.47; 99.42]	0.9%
Hatta et al (2017) (4)	1	9	5	9	80.00	[-38.91; 97.12]	1.5%
Wong et al (2017) (3)	0	4	4	4	88.89	[-51.60; 99.19]	0.9%
Smith et al (2019) (3)	0	8	7	8	93.33	[0.78; 99.55]	0.9%
Wang et al (2019) (1)	0	8	7	8	93.33	[0.78; 99.55]	0.9%
Schön et al (2020) (1)	0	4	1	2	81.48	[-205.27; 98.88]	0.8%
Hatta et al (2024) (1)	1	3	3	3	57.14	[-43.68; 87.22]	2.6%
Hatta et al (2024) (2)	0	3	3	3	85.71	[-85.95; 98.90]	1.0%
Hatta et al (2024) (3)	0	3	3	3	85.71	[-85.95; 98.90]	1.0%
Hatta et al (2024) (4)	0	3	3	3	85.71	[-85.95; 98.90]	1.0%
Hatta et al (2024) (5)	0	2	3	3	80.00	[-138.63; 98.32]	1.0%
Hatta et al (2024) (6)	0	9	10	10	94.74	[21.85; 99.65]	0.9%
Hatta et al (2024) (7)	1	10	10	10	85.71	[37.14; 96.75]	2.1%
Hatta et al (2024) (8)	0	10	10	10	95.24	[28.79; 99.68]	0.9%
**Random effects model**		**374**		**352**	**87.85**	**[83.62; 90.99]**	**66.7%**
Heterogeneity: $I^2 = 0\%$, $\tau^2 = 0$, $p = 1.0000$							
**Vaccine = seasonal influenza vaccine with N1**							
Haynes et al (2009) (1)	0	7	6	7	92.31	[-13.60; 99.48]	0.9%
Perrone et al (2009) (1)	0	6	6	6	92.31	[-10.31; 99.46]	0.9%
Perrone et al (2009) (2)	3	6	6	6	46.15	[-9.71; 73.57]	3.9%
Price et al (2009) (1)	1	6	6	6	76.92	[6.08; 94.33]	2.2%
Price et al (2009) (2)	0	6	6	6	92.31	[-10.31; 99.46]	0.9%
Rockman et al (2013) (1)	2	4	4	4	44.44	[-26.95; 75.69]	3.6%
Rockman et al (2013) (2)	0	4	4	4	88.89	[-51.60; 99.19]	0.9%
Rockman et al (2013) (3)	0	4	4	4	88.89	[-51.60; 99.19]	0.9%
Rockman et al (2013) (4)	0	3	4	4	85.71	[-85.95; 98.90]	1.0%
Rockman et al (2013) (6)	0	4	4	4	88.89	[-51.60; 99.19]	0.9%
Rockman et al (2013) (7)	0	4	4	4	88.89	[-51.60; 99.19]	0.9%
Rockman et al (2013) (8)	5	8	4	4	35.29	[-6.31; 60.62]	4.4%
Rockman et al (2013) (11)	0	4	4	4	88.89	[-51.60; 99.19]	0.9%
Rockman et al (2013) (12)	0	4	4	4	88.89	[-51.60; 99.19]	0.9%
Rockman et al (2013) (14)	0	4	4	4	88.89	[-51.60; 99.19]	0.9%
Park et al (2014) (1)	0	12	12	12	96.00	[39.53; 99.74]	0.9%
Park et al (2014) (3)	12	12	12	12	0.00	[-17.35; 14.79]	5.1%
Hatta et al (2017) (1)	1	9	5	9	80.00	[-38.91; 97.12]	1.5%
Hatta et al (2017) (2)	1	9	5	9	80.00	[-38.91; 97.12]	1.5%
**Random effects model**		**116**		**113**	**72.78**	**[53.99; 83.90]**	**33.3%**
Heterogeneity: $I^2 = 62.1\%$, $\tau^2 = 0.5272$, $p = 0.0002$							
**Random effects model**		**490**		**465**	**84.25**	**[79.16; 88.09]**	**100.0%**
Heterogeneity: $I^2 = 55.7\%$, $\tau^2 = 0.3956$, $p < 0.0001$							
Test for subgroup differences: $\chi^2_1 = 6.85$, df = 1 ($p = 0.0088$)							

Supplementary Figure 5. Vaccine efficacy in H5N1 vaccine trials without seroprotection versus N1-containing seasonal influenza vaccine trials.

Study	Vaccinated Events	Vaccinated Total	Control Events	Control Total	VE	95%-CI	Weight (common)	Weight (random)
**Vaccine = H5N1 vaccine not achieving seroprotection**								
Hoffmann et al (2005) (1)	0	3	3	3	85.71	[-85.95; 98.90]	0.7%	0.6%
Hoffmann et al (2005) (2)	0	3	3	3	85.71	[-85.95; 98.90]	0.7%	0.6%
Govorkova et al (2006) (1)	0	3	3	3	85.71	[-85.95; 98.90]	0.7%	0.6%
Govorkova et al (2006) (2)	0	3	3	3	85.71	[-85.95; 98.90]	0.7%	0.6%
Govorkova et al (2006) (3)	0	3	3	3	85.71	[-85.95; 98.90]	0.7%	0.6%
Baras et al (2008) (1)	6	6	6	6	0.00	[-36.87; 26.94]	1.3%	2.9%
Baras et al (2008) (2)	1	6	6	6	76.92	[6.08; 94.33]	1.3%	1.4%
Baras et al (2008) (4)	0	5	6	6	90.91	[-27.75; 99.35]	1.2%	0.6%
Baras et al (2008) (5)	0	6	6	6	92.31	[-10.31; 99.46]	1.3%	0.6%
Lalor et al (2008) (1)	5	6	6	6	15.38	[-17.44; 39.03]	1.3%	2.9%
Lalor et al (2008) (4)	0	6	6	6	92.31	[-10.31; 99.46]	1.3%	0.6%
Mahmood et al (2008) (1)	0	4	3	4	85.71	[-104.14; 99.00]	0.7%	0.6%
Mahmood et al (2008) (9)	0	4	4	4	88.89	[-51.60; 99.19]	0.9%	0.6%
Forrest et al (2009) (3)	0	5	5	5	90.91	[-27.75; 99.35]	1.1%	0.6%
Forrest et al (2009) (6)	0	5	5	5	90.91	[-27.75; 99.35]	1.1%	0.6%
Forrest et al (2009) (7)	0	5	5	5	90.91	[-27.75; 99.35]	1.1%	0.6%
Forrest et al (2009) (8)	0	5	5	5	90.91	[-27.75; 99.35]	1.1%	0.6%
Middleton et al (2009) (1)	2	4	3	4	33.33	[-106.70; 78.50]	0.6%	1.7%
Middleton et al (2009) (10)	0	4	4	4	88.89	[-51.60; 99.19]	0.9%	0.6%
Middleton et al (2009) (11)	0	4	4	4	88.89	[-51.60; 99.19]	0.9%	0.6%
Middleton et al (2009) (12)	0	4	3	4	85.71	[-104.14; 99.00]	0.7%	0.6%
Middleton et al (2009) (13)	0	4	3	4	85.71	[-104.14; 99.00]	0.7%	0.6%
Middleton et al (2009) (14)	0	4	3	4	85.71	[-104.14; 99.00]	0.7%	0.6%
Middleton et al (2009) (15)	0	4	3	4	85.71	[-104.14; 99.00]	0.7%	0.6%
Middleton et al (2009) (16)	1	4	3	4	66.67	[-99.49; 94.43]	0.6%	1.0%
Middleton et al (2009) (17)	1	4	3	4	66.67	[-99.49; 94.43]	0.6%	1.0%
Middleton et al (2009) (2)	3	4	3	4	0.00	[-122.59; 55.07]	0.6%	2.2%
Middleton et al (2009) (3)	2	4	3	4	33.33	[-106.70; 78.50]	0.6%	1.7%
Middleton et al (2009) (5)	0	4	4	4	88.89	[-51.60; 99.19]	0.9%	0.6%
Middleton et al (2009) (7)	0	4	4	4	88.89	[-51.60; 99.19]	0.9%	0.6%
Middleton et al (2009) (8)	0	4	4	4	88.89	[-51.60; 99.19]	0.9%	0.6%
Middleton et al (2009) (9)	0	4	4	4	88.89	[-51.60; 99.19]	0.9%	0.6%
Song et al (2009) (1)	0	4	3	4	85.71	[-104.14; 99.00]	0.7%	0.6%
Rao et al (2010) (3)	4	4	5	5	0.00	[-52.22; 34.31]	1.0%	2.8%
Rao et al (2010) (4)	5	5	5	5	0.00	[-45.32; 31.19]	1.1%	2.9%
Rao et al (2010) (5)	4	4	5	5	0.00	[-52.22; 34.31]	1.0%	2.8%
Gustin et al (2011) (3)	0	6	3	6	85.71	[-124.93; 99.09]	0.7%	0.5%
Layton et al (2011) (11)	14	14	14	14	0.00	[-14.75; 12.85]	3.0%	3.1%
Layton et al (2011) (5)	0	6	3	3	92.31	[-10.31; 99.46]	0.9%	0.6%
Layton et al (2011) (6)	5	14	14	14	62.07	[26.73; 80.36]	3.0%	2.4%
Layton et al (2011) (7)	8	14	14	14	41.38	[9.66; 61.96]	3.0%	2.8%
Layton et al (2011) (8)	8	14	14	14	41.38	[9.66; 61.96]	3.0%	2.8%
Liu et al (2012) (1)	0	6	5	6	90.91	[-33.01; 99.38]	1.1%	0.6%
Liu et al (2012) (2)	0	6	5	6	90.91	[-33.01; 99.38]	1.1%	0.6%
Liu et al (2012) (3)	0	6	5	6	90.91	[-33.01; 99.38]	1.1%	0.6%
Liu et al (2012) (4)	0	6	5	6	90.91	[-33.01; 99.38]	1.1%	0.6%
Vela et al (2012) (1)	4	8	8	8	47.06	[0.22; 71.91]	1.7%	2.5%
Vela et al (2012) (2)	6	8	8	8	23.53	[-11.03; 47.33]	1.7%	2.9%
Vela et al (2012) (3)	0	8	8	8	94.12	[13.43; 99.60]	1.7%	0.6%
Vela et al (2012) (4)	0	8	8	8	94.12	[13.43; 99.60]	1.7%	0.6%
Verma et al (2012) (1)	4	6	6	6	30.77	[-15.58; 58.53]	1.3%	2.7%
Verma et al (2012) (3)	0	6	6	6	92.31	[-10.31; 99.46]	1.3%	0.6%
Verma et al (2012) (4)	5	6	6	6	15.38	[-17.44; 39.03]	1.3%	2.9%
Verma et al (2012) (6)	0	6	6	6	92.31	[-10.31; 99.46]	1.3%	0.6%
Mann et al (2014) (1)	2	6	5	6	60.00	[-31.07; 87.79]	1.0%	1.7%
Mann et al (2014) (2)	0	6	5	6	90.91	[-33.01; 99.38]	1.1%	0.6%
Baz et al (2015) (1)	0	4	4	4	88.89	[-51.60; 99.19]	0.9%	0.6%
Cox et al (2015) (1)	0	6	5	6	90.91	[-33.01; 99.38]	1.1%	0.6%
Major et al (2015) (1)	2	7	4	8	42.86	[-122.85; 85.35]	0.8%	1.4%
Major et al (2015) (2)	0	7	4	8	87.41	[-97.34; 99.20]	0.9%	0.5%
Liu et al (2016) (2)	3	6	6	6	46.15	[-9.71; 73.57]	1.3%	2.4%
Wong et al (2017) (1)	1	4	4	4	66.67	[-23.13; 90.98]	0.9%	1.5%
Wong et al (2017) (2)	0	4	4	4	88.89	[-51.60; 99.19]	0.9%	0.6%
Smith et al (2019) (1)	0	8	4	6	91.50	[-31.25; 99.45]	1.0%	0.6%
Smith et al (2019) (2)	1	8	4	6	81.25	[-27.73; 97.25]	0.9%	0.9%
Smith et al (2019) (4)	0	8	7	8	93.33	[0.78; 99.55]	1.5%	0.6%
Wang et al (2019) (2)	2	8	7	8	71.43	[2.40; 91.64]	1.4%	1.6%
**Common effect model**		**385**		**383**	**65.87**	**[60.47; 70.54]**	**76.9%**	**.**
**Random effects model**					**62.73**	**[52.30; 70.88]**	**.**	**79.4%**
Heterogeneity: $I^2 = 52.3\%$, $\tau^2 = 0.3955$, $p < 0.0001$								
**Vaccine = seasonal influenza vaccine with N1**								
Haynes et al (2009) (1)	0	7	6	7	92.31	[-13.60; 99.48]	1.3%	0.6%
Perrone et al (2009) (1)	0	6	6	6	92.31	[-10.31; 99.46]	1.3%	0.6%
Perrone et al (2009) (2)	3	6	6	6	46.15	[-9.71; 73.57]	1.3%	2.4%
Price et al (2009) (1)	1	6	6	6	76.92	[6.08; 94.33]	1.3%	1.4%
Price et al (2009) (2)	0	6	6	6	92.31	[-10.31; 99.46]	1.3%	0.6%
Rockman et al (2013) (1)	2	4	4	4	44.44	[-26.95; 75.69]	0.9%	2.2%
Rockman et al (2013) (11)	0	4	4	4	88.89	[-51.60; 99.19]	0.9%	0.6%
Rockman et al (2013) (12)	0	4	4	4	88.89	[-51.60; 99.19]	0.9%	0.6%
Rockman et al (2013) (14)	0	4	4	4	88.89	[-51.60; 99.19]	0.9%	0.6%
Rockman et al (2013) (2)	0	4	4	4	88.89	[-51.60; 99.19]	0.9%	0.6%
Rockman et al (2013) (3)	0	4	4	4	88.89	[-51.60; 99.19]	0.9%	0.6%
Rockman et al (2013) (4)	0	3	4	4	85.71	[-85.95; 98.90]	0.8%	0.6%
Rockman et al (2013) (6)	0	4	4	4	88.89	[-51.60; 99.19]	0.9%	0.6%
Rockman et al (2013) (7)	0	4	4	4	88.89	[-51.60; 99.19]	0.9%	0.6%
Rockman et al (2013) (8)	5	8	4	4	35.29	[-6.31; 60.62]	1.2%	2.7%
Park et al (2014) (1)	0	12	12	12	96.00	[39.53; 99.74]	2.5%	0.6%
Park et al (2014) (3)	12	12	12	12	0.00	[-17.35; 14.79]	2.5%	3.0%
Hatta et al (2017) (1)	1	9	5	9	80.00	[-38.91; 97.12]	1.0%	0.9%
Hatta et al (2017) (2)	1	9	5	9	80.00	[-38.91; 97.12]	1.0%	0.9%
**Common effect model**		**116**		**113**	**71.77**	**[61.74; 79.16]**	**23.1%**	**.**
**Random effects model**					**72.78**	**[53.99; 83.90]**	**.**	**20.6%**
Heterogeneity: $I^2 = 62.1\%$, $\tau^2 = 0.5272$, $p = 0.0002$								
**Common effect model**		**501**		**496**	**67.23**	**[62.59; 71.30]**	**100.0%**	**.**
**Random effects model**					**65.13**	**[56.36; 72.14]**	**.**	**100.0%**

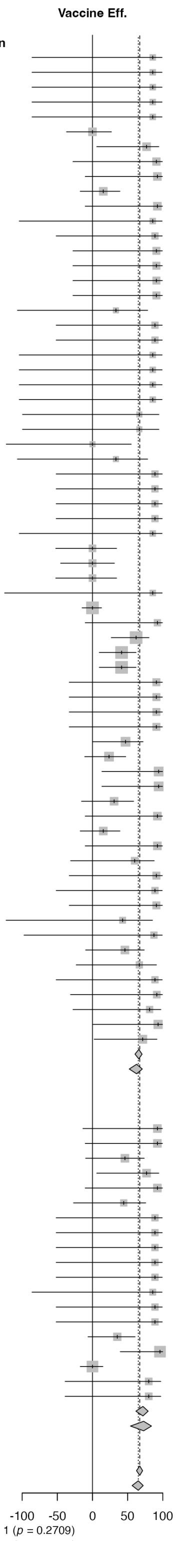

Heterogeneity: $I^2 = 54.4\%$, $\tau^2 = 0.4228$, $p < 0.0001$
Test for subgroup differences (common effect): $\chi^2_1 = 1.21$, df = 1 ($p = 0.2709$)
Test for subgroup differences (random effects): $\chi^2_1 = 1.13$, df = 1 ($p = 0.2884$)

Supplementary Figure 6. Vaccine efficacy in all vaccines: subgroup analysis of the clade of the challenge virus.

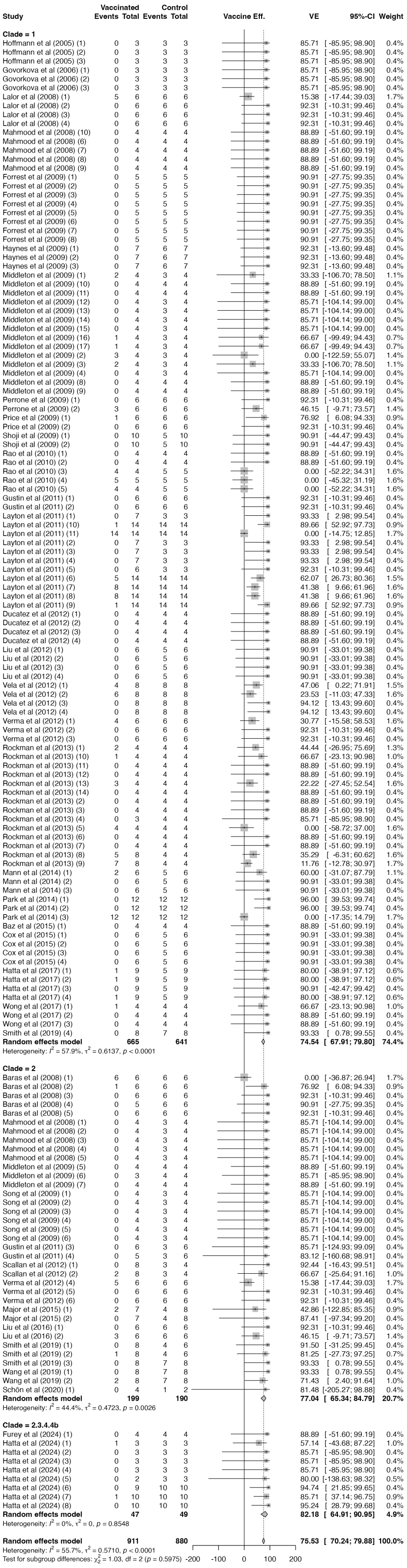

Study	Vaccinated Events	Vaccinated Total	Control Events	Control Total	VE	95%-CI	Weight
**Clade = 1**							
Hoffmann et al (2005) (1)	0	3	3	3	85.71	[-85.95; 98.90]	0.4%
Hoffmann et al (2005) (2)	0	3	3	3	85.71	[-85.95; 98.90]	0.4%
Hoffmann et al (2005) (3)	0	3	3	3	85.71	[-85.95; 98.90]	0.4%
Govorkova et al (2006) (1)	0	3	3	3	85.71	[-85.95; 98.90]	0.4%
Govorkova et al (2006) (2)	0	3	3	3	85.71	[-85.95; 98.90]	0.4%
Govorkova et al (2006) (3)	0	3	3	3	85.71	[-85.95; 98.90]	0.4%
Lalor et al (2008) (1)	5	6	6	6	15.38	[-17.44; 39.03]	1.7%
Lalor et al (2008) (2)	0	6	6	6	92.31	[-10.31; 99.46]	0.4%
Lalor et al (2008) (3)	0	6	6	6	92.31	[-10.31; 99.46]	0.4%
Lalor et al (2008) (4)	0	6	6	6	92.31	[-10.31; 99.46]	0.4%
Mahmood et al (2008) (10)	0	4	4	4	88.89	[-51.60; 99.19]	0.4%
Mahmood et al (2008) (6)	0	4	4	4	88.89	[-51.60; 99.19]	0.4%
Mahmood et al (2008) (7)	0	4	4	4	88.89	[-51.60; 99.19]	0.4%
Mahmood et al (2008) (8)	0	4	4	4	88.89	[-51.60; 99.19]	0.4%
Mahmood et al (2008) (9)	0	4	4	4	88.89	[-51.60; 99.19]	0.4%
Forrest et al (2009) (1)	0	5	5	5	90.91	[-27.75; 99.35]	0.4%
Forrest et al (2009) (2)	0	5	5	5	90.91	[-27.75; 99.35]	0.4%
Forrest et al (2009) (3)	0	5	5	5	90.91	[-27.75; 99.35]	0.4%
Forrest et al (2009) (4)	0	5	5	5	90.91	[-27.75; 99.35]	0.4%
Forrest et al (2009) (5)	0	5	5	5	90.91	[-27.75; 99.35]	0.4%
Forrest et al (2009) (6)	0	5	5	5	90.91	[-27.75; 99.35]	0.4%
Forrest et al (2009) (7)	0	5	5	5	90.91	[-27.75; 99.35]	0.4%
Forrest et al (2009) (8)	0	5	5	5	90.91	[-27.75; 99.35]	0.4%
Haynes et al (2009) (1)	0	7	6	7	92.31	[-13.60; 99.48]	0.4%
Haynes et al (2009) (2)	0	7	6	7	92.31	[-13.60; 99.48]	0.4%
Haynes et al (2009) (3)	0	7	6	7	92.31	[-13.60; 99.48]	0.4%
Middleton et al (2009) (1)	2	4	3	4	33.33	[-106.70; 78.50]	1.1%
Middleton et al (2009) (10)	0	4	4	4	88.89	[-51.60; 99.19]	0.4%
Middleton et al (2009) (11)	0	4	4	4	88.89	[-51.60; 99.19]	0.4%
Middleton et al (2009) (12)	0	4	3	4	85.71	[-104.14; 99.00]	0.4%
Middleton et al (2009) (13)	0	4	3	4	85.71	[-104.14; 99.00]	0.4%
Middleton et al (2009) (14)	0	4	3	4	85.71	[-104.14; 99.00]	0.4%
Middleton et al (2009) (15)	0	4	3	4	85.71	[-104.14; 99.00]	0.4%
Middleton et al (2009) (16)	1	4	3	4	66.67	[-99.49; 94.43]	0.7%
Middleton et al (2009) (17)	1	4	3	4	66.67	[-99.49; 94.43]	0.7%
Middleton et al (2009) (2)	3	4	3	4	0.00	[-122.59; 55.07]	1.4%
Middleton et al (2009) (3)	2	4	3	4	33.33	[-106.70; 78.50]	1.1%
Middleton et al (2009) (4)	0	4	3	4	85.71	[-104.14; 99.00]	0.4%
Middleton et al (2009) (8)	0	4	4	4	88.89	[-51.60; 99.19]	0.4%
Middleton et al (2009) (9)	0	4	4	4	88.89	[-51.60; 99.19]	0.4%
Perrone et al (2009) (1)	0	6	6	6	92.31	[-10.31; 99.46]	0.4%
Perrone et al (2009) (2)	3	6	6	6	46.15	[-9.71; 73.57]	1.4%
Price et al (2009) (1)	1	6	6	6	76.92	[6.08; 94.33]	0.9%
Price et al (2009) (2)	0	6	6	6	92.31	[-10.31; 99.46]	0.4%
Shoji et al (2009) (1)	0	10	5	10	90.91	[-44.47; 99.43]	0.4%
Shoji et al (2009) (2)	0	10	5	10	90.91	[-44.47; 99.43]	0.4%
Rao et al (2010) (1)	0	4	4	4	88.89	[-51.60; 99.19]	0.4%
Rao et al (2010) (2)	0	4	4	4	88.89	[-51.60; 99.19]	0.4%
Rao et al (2010) (3)	4	4	5	5	0.00	[-52.22; 34.31]	1.6%
Rao et al (2010) (4)	5	5	5	5	0.00	[-45.32; 31.19]	1.6%
Rao et al (2010) (5)	4	4	5	5	0.00	[-52.22; 34.31]	1.6%
Gustin et al (2011) (1)	0	6	6	6	92.31	[-10.31; 99.46]	0.4%
Gustin et al (2011) (2)	0	6	6	6	92.31	[-10.31; 99.46]	0.4%
Layton et al (2011) (1)	0	7	3	3	93.33	[2.98; 99.54]	0.4%
Layton et al (2011) (10)	1	14	14	14	89.66	[52.92; 97.73]	0.9%
Layton et al (2011) (11)	14	14	14	14	0.00	[-14.75; 12.85]	1.7%
Layton et al (2011) (2)	0	7	3	3	93.33	[2.98; 99.54]	0.4%
Layton et al (2011) (3)	0	7	3	3	93.33	[2.98; 99.54]	0.4%
Layton et al (2011) (4)	0	7	3	3	93.33	[2.98; 99.54]	0.4%
Layton et al (2011) (5)	0	6	3	3	92.31	[-10.31; 99.46]	0.4%
Layton et al (2011) (6)	5	14	14	14	62.07	[26.73; 80.36]	1.5%
Layton et al (2011) (7)	8	14	14	14	41.38	[9.66; 61.96]	1.6%
Layton et al (2011) (8)	8	14	14	14	41.38	[9.66; 61.96]	1.6%
Layton et al (2011) (9)	1	14	14	14	89.66	[52.92; 97.73]	0.9%
Ducatez et al (2012) (1)	0	4	4	4	88.89	[-51.60; 99.19]	0.4%
Ducatez et al (2012) (2)	0	4	4	4	88.89	[-51.60; 99.19]	0.4%
Ducatez et al (2012) (3)	0	4	4	4	88.89	[-51.60; 99.19]	0.4%
Ducatez et al (2012) (4)	0	4	4	4	88.89	[-51.60; 99.19]	0.4%
Liu et al (2012) (1)	0	6	5	6	90.91	[-33.01; 99.38]	0.4%
Liu et al (2012) (2)	0	6	5	6	90.91	[-33.01; 99.38]	0.4%
Liu et al (2012) (3)	0	6	5	6	90.91	[-33.01; 99.38]	0.4%
Liu et al (2012) (4)	0	6	5	6	90.91	[-33.01; 99.38]	0.4%
Vela et al (2012) (1)	4	8	8	8	47.06	[0.22; 71.91]	1.5%
Vela et al (2012) (2)	6	8	8	8	23.53	[-11.03; 47.33]	1.6%
Vela et al (2012) (3)	0	8	8	8	94.12	[13.43; 99.60]	0.4%
Vela et al (2012) (4)	0	8	8	8	94.12	[13.43; 99.60]	0.4%
Verma et al (2012) (1)	4	6	6	6	30.77	[-15.58; 58.53]	1.6%
Verma et al (2012) (2)	0	6	6	6	92.31	[-10.31; 99.46]	0.4%
Verma et al (2012) (3)	0	6	6	6	92.31	[-10.31; 99.46]	0.4%
Rockman et al (2013) (1)	2	4	4	4	44.44	[-26.95; 75.69]	1.3%
Rockman et al (2013) (10)	1	4	4	4	66.67	[-23.13; 90.98]	1.0%
Rockman et al (2013) (11)	0	4	4	4	88.89	[-51.60; 99.19]	0.4%
Rockman et al (2013) (12)	0	4	4	4	88.89	[-51.60; 99.19]	0.4%
Rockman et al (2013) (13)	3	4	4	4	22.22	[-27.45; 52.54]	1.6%
Rockman et al (2013) (14)	0	4	4	4	88.89	[-51.60; 99.19]	0.4%
Rockman et al (2013) (2)	0	4	4	4	88.89	[-51.60; 99.19]	0.4%
Rockman et al (2013) (3)	0	4	4	4	88.89	[-51.60; 99.19]	0.4%
Rockman et al (2013) (4)	0	3	4	4	85.71	[-85.95; 98.90]	0.4%
Rockman et al (2013) (5)	4	4	4	4	0.00	[-58.72; 37.00]	1.6%
Rockman et al (2013) (6)	0	4	4	4	88.89	[-51.60; 99.19]	0.4%
Rockman et al (2013) (7)	0	4	4	4	88.89	[-51.60; 99.19]	0.4%
Rockman et al (2013) (8)	5	8	4	4	35.29	[-6.31; 60.62]	1.6%
Rockman et al (2013) (9)	7	8	4	4	11.76	[-12.78; 30.97]	1.7%
Mann et al (2014) (1)	2	6	5	6	60.00	[-31.07; 87.79]	1.1%
Mann et al (2014) (2)	0	6	5	6	90.91	[-33.01; 99.38]	0.4%
Mann et al (2014) (3)	0	6	5	6	90.91	[-33.01; 99.38]	0.4%
Park et al (2014) (1)	0	12	12	12	96.00	[39.53; 99.74]	0.4%
Park et al (2014) (2)	0	12	12	12	96.00	[39.53; 99.74]	0.4%
Park et al (2014) (3)	12	12	12	12	0.00	[-17.35; 14.79]	1.7%
Baz et al (2015) (1)	0	4	4	4	88.89	[-51.60; 99.19]	0.4%
Cox et al (2015) (1)	0	6	5	6	90.91	[-33.01; 99.38]	0.4%
Cox et al (2015) (2)	0	6	5	6	90.91	[-33.01; 99.38]	0.4%
Cox et al (2015) (3)	0	6	5	6	90.91	[-33.01; 99.38]	0.4%
Cox et al (2015) (4)	0	6	5	6	90.91	[-33.01; 99.38]	0.4%
Hatta et al (2017) (1)	1	9	5	9	80.00	[-38.91; 97.12]	0.6%
Hatta et al (2017) (2)	1	9	5	9	80.00	[-38.91; 97.12]	0.6%
Hatta et al (2017) (3)	0	9	5	9	90.91	[-42.47; 99.42]	0.4%
Hatta et al (2017) (4)	1	9	5	9	80.00	[-38.91; 97.12]	0.6%
Wong et al (2017) (1)	1	4	4	4	66.67	[-23.13; 90.98]	1.0%
Wong et al (2017) (2)	0	4	4	4	88.89	[-51.60; 99.19]	0.4%
Wong et al (2017) (3)	0	4	4	4	88.89	[-51.60; 99.19]	0.4%
Smith et al (2019) (4)	0	8	7	8	93.33	[0.78; 99.55]	0.4%
**Random effects model**		**665**		**641**	**74.54**	**[67.91; 79.80]**	**74.4%**
Heterogeneity: $I^2 = 57.9\%$, $\tau^2 = 0.6137$, $p < 0.0001$							
**Clade = 2**							
Baras et al (2008) (1)	6	6	6	6	0.00	[-36.87; 26.94]	1.7%
Baras et al (2008) (2)	1	6	6	6	76.92	[6.08; 94.33]	0.9%
Baras et al (2008) (3)	0	6	6	6	92.31	[-10.31; 99.46]	0.4%
Baras et al (2008) (4)	0	5	6	6	90.91	[-27.75; 99.35]	0.4%
Baras et al (2008) (5)	0	6	6	6	92.31	[-10.31; 99.46]	0.4%
Mahmood et al (2008) (1)	0	4	3	4	85.71	[-104.14; 99.00]	0.4%
Mahmood et al (2008) (2)	0	4	3	4	85.71	[-104.14; 99.00]	0.4%
Mahmood et al (2008) (3)	0	4	3	4	85.71	[-104.14; 99.00]	0.4%
Mahmood et al (2008) (4)	0	4	3	4	85.71	[-104.14; 99.00]	0.4%
Mahmood et al (2008) (5)	0	4	3	4	85.71	[-104.14; 99.00]	0.4%
Middleton et al (2009) (5)	0	4	4	4	88.89	[-51.60; 99.19]	0.4%
Middleton et al (2009) (6)	0	3	4	4	85.71	[-85.95; 98.90]	0.4%
Middleton et al (2009) (7)	0	4	4	4	88.89	[-51.60; 99.19]	0.4%
Song et al (2009) (1)	0	4	3	4	85.71	[-104.14; 99.00]	0.4%
Song et al (2009) (2)	0	4	3	4	85.71	[-104.14; 99.00]	0.4%
Song et al (2009) (3)	0	4	3	4	85.71	[-104.14; 99.00]	0.4%
Song et al (2009) (4)	0	4	3	4	85.71	[-104.14; 99.00]	0.4%
Song et al (2009) (5)	0	4	3	4	85.71	[-104.14; 99.00]	0.4%
Song et al (2009) (6)	0	4	3	4	85.71	[-104.14; 99.00]	0.4%
Gustin et al (2011) (3)	0	6	3	6	85.71	[-124.93; 99.09]	0.4%
Gustin et al (2011) (4)	0	5	3	6	83.12	[-160.68; 98.91]	0.4%
Scallan et al (2012) (1)	0	8	3	4	92.44	[-16.43; 99.51]	0.4%
Scallan et al (2012) (2)	2	8	3	4	66.67	[-25.64; 91.16]	1.0%
Verma et al (2012) (4)	5	6	6	6	15.38	[-17.44; 39.03]	1.7%
Verma et al (2012) (5)	0	6	6	6	92.31	[-10.31; 99.46]	0.4%
Verma et al (2012) (6)	0	6	6	6	92.31	[-10.31; 99.46]	0.4%
Major et al (2015) (1)	2	7	4	8	42.86	[-122.85; 85.35]	0.9%
Major et al (2015) (2)	0	7	4	8	87.41	[-97.34; 99.20]	0.4%
Liu et al (2016) (1)	0	6	6	6	92.31	[-10.31; 99.46]	0.4%
Liu et al (2016) (2)	3	6	6	6	46.15	[-9.71; 73.57]	1.4%
Smith et al (2019) (1)	0	8	4	6	91.50	[-31.25; 99.45]	0.4%
Smith et al (2019) (2)	1	8	4	6	81.25	[-27.73; 97.25]	0.7%
Smith et al (2019) (3)	0	8	7	8	93.33	[0.78; 99.55]	0.4%
Wang et al (2019) (1)	0	8	7	8	93.33	[0.78; 99.55]	0.4%
Wang et al (2019) (2)	2	8	7	8	71.43	[2.40; 91.64]	1.0%
Schön et al (2020) (1)	0	4	1	2	81.48	[-205.27; 98.88]	0.4%
**Random effects model**		**199**		**190**	**77.04**	**[65.34; 84.79]**	**20.7%**
Heterogeneity: $I^2 = 44.4\%$, $\tau^2 = 0.4723$, $p = 0.0026$							
**Clade = 2.3.4.4b**							
Furey et al (2024) (1)	0	4	4	4	88.89	[-51.60; 99.19]	0.4%
Hatta et al (2024) (1)	1	3	3	3	57.14	[-43.68; 87.22]	1.0%
Hatta et al (2024) (2)	0	3	3	3	85.71	[-85.95; 98.90]	0.4%
Hatta et al (2024) (3)	0	3	3	3	85.71	[-85.95; 98.90]	0.4%
Hatta et al (2024) (4)	0	3	3	3	85.71	[-85.95; 98.90]	0.4%
Hatta et al (2024) (5)	0	2	3	3	80.00	[-138.63; 98.32]	0.5%
Hatta et al (2024) (6)	0	9	10	10	94.74	[21.85; 99.65]	0.4%
Hatta et al (2024) (7)	1	10	10	10	85.71	[37.14; 96.75]	0.9%
Hatta et al (2024) (8)	0	10	10	10	95.24	[28.79; 99.68]	0.4%
**Random effects model**		**47**		**49**	**82.18**	**[64.91; 90.95]**	**4.9%**
Heterogeneity: $I^2 = 0\%$, $\tau^2 = 0$, $p = 0.8548$							
**Random effects model**		**911**		**880**	**75.53**	**[70.24; 79.88]**	**100.0%**
Heterogeneity: $I^2 = 55.7\%$, $\tau^2 = 0.5710$, $p < 0.0001$							
Test for subgroup differences: $\chi^2_2 = 1.03$, df = 2 ($p = 0.5975$)							

Supplementary Figure 7. Vaccine efficacy of H5N1 vaccines: subgroup analysis by the time interval from vaccination to challenge.

Note. The time interval from vaccination to viral challenge was categorized as short (≤7 days), medium (8–30 days), and long (≥31 days).

Study	Vaccinated Events	Vaccinated Total	Control Events	Control Total	VE	95%-CI	Weight (common)	Weight (random)
**DPI_group = short**								
Shoji et al (2009) (1)	0	10	5	10	90.91	[-44.47; 99.43]	0.8%	0.5%
Shoji et al (2009) (2)	0	10	5	10	90.91	[-44.47; 99.43]	0.8%	0.5%
Schön et al (2020) (1)	0	4	1	2	81.48	[-205.27; 98.88]	0.3%	0.5%
Forrest et al (2009) (1)	0	5	5	5	90.91	[-27.75; 99.35]	0.8%	0.5%
Forrest et al (2009) (2)	0	5	5	5	90.91	[-27.75; 99.35]	0.8%	0.5%
Forrest et al (2009) (3)	0	5	5	5	90.91	[-27.75; 99.35]	0.8%	0.5%
Forrest et al (2009) (4)	0	5	5	5	90.91	[-27.75; 99.35]	0.8%	0.5%
Forrest et al (2009) (5)	0	5	5	5	90.91	[-27.75; 99.35]	0.8%	0.5%
Forrest et al (2009) (6)	0	5	5	5	90.91	[-27.75; 99.35]	0.8%	0.5%
Forrest et al (2009) (7)	0	5	5	5	90.91	[-27.75; 99.35]	0.8%	0.5%
Forrest et al (2009) (8)	0	5	5	5	90.91	[-27.75; 99.35]	0.8%	0.5%
Govorkova et al (2006) (1)	0	3	3	3	85.71	[-85.95; 98.90]	0.5%	0.5%
Govorkova et al (2006) (3)	0	3	3	3	85.71	[-85.95; 98.90]	0.5%	0.5%
Govorkova et al (2006) (2)	0	3	3	3	85.71	[-85.95; 98.90]	0.5%	0.5%
**Common effect model**		**73**		**71**	**89.80**	**[79.03; 95.03]**	**9.3%**	**.**
**Random effects model**					**89.45**	**[78.56; 94.81]**	**.**	**7.2%**
Heterogeneity: $I^2 = 0\%$, $\tau^2 = 0$, $p = 1.0000$								
**DPI_group = medium**								
Layton et al (2011) (10)	1	14	14	14	89.66	[52.92; 97.73]	2.0%	1.1%
Layton et al (2011) (11)	14	14	14	14	0.00	[-14.75; 12.85]	2.0%	2.2%
Layton et al (2011) (6)	5	14	14	14	62.07	[26.73; 80.36]	2.0%	1.8%
Layton et al (2011) (7)	8	14	14	14	41.38	[9.66; 61.96]	2.0%	2.0%
Layton et al (2011) (8)	8	14	14	14	41.38	[9.66; 61.96]	2.0%	2.0%
Layton et al (2011) (9)	1	14	14	14	89.66	[52.92; 97.73]	2.0%	1.1%
Layton et al (2011) (1)	0	7	3	3	93.33	[2.98; 99.54]	0.6%	0.5%
Layton et al (2011) (2)	0	7	3	3	93.33	[2.98; 99.54]	0.6%	0.5%
Layton et al (2011) (3)	0	7	3	3	93.33	[2.98; 99.54]	0.6%	0.5%
Layton et al (2011) (4)	0	7	3	3	93.33	[2.98; 99.54]	0.6%	0.5%
Cox et al (2015) (1)	0	6	5	6	90.91	[-33.01; 99.38]	0.8%	0.5%
Cox et al (2015) (2)	0	6	5	6	90.91	[-33.01; 99.38]	0.8%	0.5%
Cox et al (2015) (3)	0	6	5	6	90.91	[-33.01; 99.38]	0.8%	0.5%
Cox et al (2015) (4)	0	6	5	6	90.91	[-33.01; 99.38]	0.8%	0.5%
Vela et al (2012) (1)	4	8	8	8	47.06	[0.22; 71.91]	1.2%	1.8%
Vela et al (2012) (2)	6	8	8	8	23.53	[-11.03; 47.33]	1.2%	2.1%
Vela et al (2012) (3)	0	8	8	8	94.12	[13.43; 99.60]	1.2%	0.5%
Vela et al (2012) (4)	0	8	8	8	94.12	[13.43; 99.60]	1.2%	0.5%
Hatta et al (2017) (3)	0	9	5	9	90.91	[-42.47; 99.42]	0.8%	0.5%
Scallan et al (2012) (1)	0	8	3	4	92.44	[-16.43; 99.51]	0.6%	0.5%
Scallan et al (2012) (2)	2	8	3	4	66.67	[-25.64; 91.16]	0.5%	1.2%
Major et al (2015) (1)	2	7	4	8	42.86	[-122.85; 85.35]	0.5%	1.2%
Major et al (2015) (2)	0	7	4	8	87.41	[-97.34; 99.20]	0.6%	0.5%
Liu et al (2012) (1)	0	6	5	6	90.91	[-33.01; 99.38]	0.8%	0.5%
Liu et al (2012) (2)	0	6	5	6	90.91	[-33.01; 99.38]	0.8%	0.5%
Liu et al (2012) (3)	0	6	5	6	90.91	[-33.01; 99.38]	0.8%	0.5%
Liu et al (2012) (4)	0	6	5	6	90.91	[-33.01; 99.38]	0.8%	0.5%
Smith et al (2019) (2)	1	8	4	6	81.25	[-27.73; 97.25]	0.6%	0.8%
Smith et al (2019) (1)	0	8	4	6	91.50	[-31.25; 99.45]	0.7%	0.5%
Smith et al (2019) (3)	0	8	7	8	93.33	[0.78; 99.55]	1.0%	0.5%
Smith et al (2019) (4)	0	8	7	8	93.33	[0.78; 99.55]	1.0%	0.5%
Mann et al (2014) (1)	2	6	5	6	60.00	[-31.07; 87.79]	0.7%	1.3%
Mann et al (2014) (2)	0	6	5	6	90.91	[-33.01; 99.38]	0.8%	0.5%
Mann et al (2014) (3)	0	6	5	6	90.91	[-33.01; 99.38]	0.8%	0.5%
Verma et al (2012) (1)	4	6	6	6	30.77	[-15.58; 58.53]	0.9%	1.9%
Verma et al (2012) (2)	0	6	6	6	92.31	[-10.31; 99.46]	0.9%	0.5%
Verma et al (2012) (3)	0	6	6	6	92.31	[-10.31; 99.46]	0.9%	0.5%
Verma et al (2012) (4)	5	6	6	6	15.38	[-17.44; 39.03]	0.9%	2.1%
Verma et al (2012) (5)	0	6	6	6	92.31	[-10.31; 99.46]	0.9%	0.5%
Verma et al (2012) (6)	0	6	6	6	92.31	[-10.31; 99.46]	0.9%	0.5%
Wang et al (2019) (1)	0	8	7	8	93.33	[0.78; 99.55]	1.0%	0.5%
Wang et al (2019) (2)	2	8	7	8	71.43	[2.40; 91.64]	1.0%	1.3%
Song et al (2009) (1)	0	4	3	4	85.71	[-104.14; 99.00]	0.5%	0.5%
Song et al (2009) (2)	0	4	3	4	85.71	[-104.14; 99.00]	0.5%	0.5%
Song et al (2009) (3)	0	4	3	4	85.71	[-104.14; 99.00]	0.5%	0.5%
Song et al (2009) (4)	0	4	3	4	85.71	[-104.14; 99.00]	0.5%	0.5%
Song et al (2009) (5)	0	4	3	4	85.71	[-104.14; 99.00]	0.5%	0.5%
Song et al (2009) (6)	0	4	3	4	85.71	[-104.14; 99.00]	0.5%	0.5%
Mahmood et al (2008) (1)	0	4	3	4	85.71	[-104.14; 99.00]	0.5%	0.5%
Mahmood et al (2008) (10)	0	4	4	4	88.89	[-51.60; 99.19]	0.6%	0.5%
Mahmood et al (2008) (2)	0	4	3	4	85.71	[-104.14; 99.00]	0.5%	0.5%
Mahmood et al (2008) (3)	0	4	3	4	85.71	[-104.14; 99.00]	0.5%	0.5%
Mahmood et al (2008) (4)	0	4	3	4	85.71	[-104.14; 99.00]	0.5%	0.5%
Mahmood et al (2008) (5)	0	4	3	4	85.71	[-104.14; 99.00]	0.5%	0.5%
Mahmood et al (2008) (6)	0	4	4	4	88.89	[-51.60; 99.19]	0.6%	0.5%
Mahmood et al (2008) (7)	0	4	4	4	88.89	[-51.60; 99.19]	0.6%	0.5%
Mahmood et al (2008) (8)	0	4	4	4	88.89	[-51.60; 99.19]	0.6%	0.5%
Mahmood et al (2008) (9)	0	4	4	4	88.89	[-51.60; 99.19]	0.6%	0.5%
Lalor et al (2008) (4)	0	6	6	6	92.31	[-10.31; 99.46]	0.9%	0.5%
Lalor et al (2008) (1)	5	6	6	6	15.38	[-17.44; 39.03]	0.9%	2.1%
Lalor et al (2008) (2)	0	6	6	6	92.31	[-10.31; 99.46]	0.9%	0.5%
Lalor et al (2008) (3)	0	6	6	6	92.31	[-10.31; 99.46]	0.9%	0.5%
Middleton et al (2009) (10)	0	4	4	4	88.89	[-51.60; 99.19]	0.6%	0.5%
Middleton et al (2009) (11)	0	4	4	4	88.89	[-51.60; 99.19]	0.6%	0.5%
Middleton et al (2009) (12)	0	4	3	4	85.71	[-104.14; 99.00]	0.5%	0.5%
Middleton et al (2009) (13)	0	4	3	4	85.71	[-104.14; 99.00]	0.5%	0.5%
Middleton et al (2009) (14)	0	4	3	4	85.71	[-104.14; 99.00]	0.5%	0.5%
Middleton et al (2009) (15)	0	4	3	4	85.71	[-104.14; 99.00]	0.5%	0.5%
Middleton et al (2009) (16)	1	4	3	4	66.67	[-99.49; 94.43]	0.4%	0.9%
Middleton et al (2009) (17)	1	4	3	4	66.67	[-99.49; 94.43]	0.4%	0.9%
Middleton et al (2009) (9)	0	4	4	4	88.89	[-51.60; 99.19]	0.6%	0.5%
Middleton et al (2009) (1)	2	4	3	4	33.33	[-106.70; 78.50]	0.4%	1.4%
Middleton et al (2009) (2)	3	4	3	4	0.00	[-122.59; 55.07]	0.4%	1.7%
Middleton et al (2009) (3)	2	4	3	4	33.33	[-106.70; 78.50]	0.4%	1.4%
Middleton et al (2009) (4)	0	4	3	4	85.71	[-104.14; 99.00]	0.5%	0.5%
Middleton et al (2009) (5)	0	4	4	4	88.89	[-51.60; 99.19]	0.6%	0.5%
Middleton et al (2009) (6)	0	3	4	4	85.71	[-85.95; 98.90]	0.5%	0.5%
Middleton et al (2009) (7)	0	4	4	4	88.89	[-51.60; 99.19]	0.6%	0.5%
Middleton et al (2009) (8)	0	4	4	4	88.89	[-51.60; 99.19]	0.6%	0.5%
Haynes et al (2009) (2)	0	7	6	7	92.31	[-13.60; 99.48]	0.9%	0.5%
Haynes et al (2009) (3)	0	7	6	7	92.31	[-13.60; 99.48]	0.9%	0.5%
Hoffmann et al (2005) (1)	0	3	3	3	85.71	[-85.95; 98.90]	0.5%	0.5%
Hoffmann et al (2005) (2)	0	3	3	3	85.71	[-85.95; 98.90]	0.5%	0.5%
Hoffmann et al (2005) (3)	0	3	3	3	85.71	[-85.95; 98.90]	0.5%	0.5%
Baras et al (2008) (1)	6	6	6	6	0.00	[-36.87; 26.94]	0.9%	2.1%
Baras et al (2008) (2)	1	6	6	6	76.92	[6.08; 94.33]	0.9%	1.1%
Baras et al (2008) (3)	0	6	6	6	92.31	[-10.31; 99.46]	0.9%	0.5%
Baras et al (2008) (4)	0	5	6	6	90.91	[-27.75; 99.35]	0.8%	0.5%
Baras et al (2008) (5)	0	6	6	6	92.31	[-10.31; 99.46]	0.9%	0.5%
Furey et al (2024) (1)	0	4	4	4	88.89	[-51.60; 99.19]	0.6%	0.5%
**Common effect model**		**544**		**520**	**75.59**	**[71.44; 79.13]**	**70.1%**	**.**
**Random effects model**					**76.43**	**[69.50; 81.78]**	**.**	**69.6%**
Heterogeneity: $I^2 = 55.8\%$, $\tau^2 = 0.5392$, $p < 0.0001$								
**DPI_group = long**								
Rao et al (2010) (1)	0	4	4	4	88.89	[-51.60; 99.19]	0.6%	0.5%
Rao et al (2010) (2)	0	4	4	4	88.89	[-51.60; 99.19]	0.6%	0.5%
Rao et al (2010) (3)	4	4	5	5	0.00	[-52.22; 34.31]	0.7%	2.0%
Rao et al (2010) (4)	5	5	5	5	0.00	[-45.32; 31.19]	0.8%	2.1%
Rao et al (2010) (5)	4	4	5	5	0.00	[-52.22; 34.31]	0.7%	2.0%
Layton et al (2011) (5)	0	6	3	3	92.31	[-10.31; 99.46]	0.6%	0.5%
Ducatez et al (2012) (1)	0	4	4	4	88.89	[-51.60; 99.19]	0.6%	0.5%
Ducatez et al (2012) (2)	0	4	4	4	88.89	[-51.60; 99.19]	0.6%	0.5%
Ducatez et al (2012) (3)	0	4	4	4	88.89	[-51.60; 99.19]	0.6%	0.5%
Ducatez et al (2012) (4)	0	4	4	4	88.89	[-51.60; 99.19]	0.6%	0.5%
Wong et al (2017) (1)	1	4	4	4	66.67	[-23.13; 90.98]	0.6%	1.2%
Wong et al (2017) (2)	0	4	4	4	88.89	[-51.60; 99.19]	0.6%	0.5%
Wong et al (2017) (3)	0	4	4	4	88.89	[-51.60; 99.19]	0.6%	0.5%
Hatta et al (2017) (4)	1	9	5	9	80.00	[-38.91; 97.12]	0.7%	0.8%
Baz et al (2015) (1)	0	4	4	4	88.89	[-51.60; 99.19]	0.6%	0.5%
Gustin et al (2011) (1)	0	6	6	6	92.31	[-10.31; 99.46]	0.9%	0.5%
Gustin et al (2011) (2)	0	6	6	6	92.31	[-10.31; 99.46]	0.9%	0.5%
Gustin et al (2011) (3)	0	6	3	6	85.71	[-124.93; 99.09]	0.5%	0.5%
Gustin et al (2011) (4)	0	5	3	6	83.12	[-160.68; 98.91]	0.4%	0.5%
Liu et al (2016) (1)	0	6	6	6	92.31	[-10.31; 99.46]	0.9%	0.5%
Liu et al (2016) (2)	3	6	6	6	46.15	[-9.71; 73.57]	0.9%	1.8%
Hatta et al (2024) (1)	1	3	3	3	57.14	[-43.68; 87.22]	0.5%	1.3%
Hatta et al (2024) (2)	0	3	3	3	85.71	[-85.95; 98.90]	0.5%	0.5%
Hatta et al (2024) (3)	0	3	3	3	85.71	[-85.95; 98.90]	0.5%	0.5%
Hatta et al (2024) (4)	0	3	3	3	85.71	[-85.95; 98.90]	0.5%	0.5%
Hatta et al (2024) (5)	0	2	3	3	80.00	[-138.63; 98.32]	0.4%	0.6%
Hatta et al (2024) (6)	0	9	10	10	94.74	[21.85; 99.65]	1.4%	0.5%
Hatta et al (2024) (7)	1	10	10	10	85.71	[37.14; 96.75]	1.4%	1.1%
Hatta et al (2024) (8)	0	10	10	10	95.24	[28.79; 99.68]	1.4%	0.5%
**Common effect model**		**146**		**148**	**76.90**	**[68.79; 82.89]**	**20.7%**	**.**
**Random effects model**					**75.19**	**[60.38; 84.46]**	**.**	**23.2%**
Heterogeneity: $I^2 = 53.2\%$, $\tau^2 = 0.6468$, $p = 0.0004$								
**Common effect model**		**763**		**739**	**77.17**	**[73.82; 80.10]**	**100.0%**	**.**
**Random effects model**					**77.45**	**[71.98; 81.85]**	**.**	**100.0%**

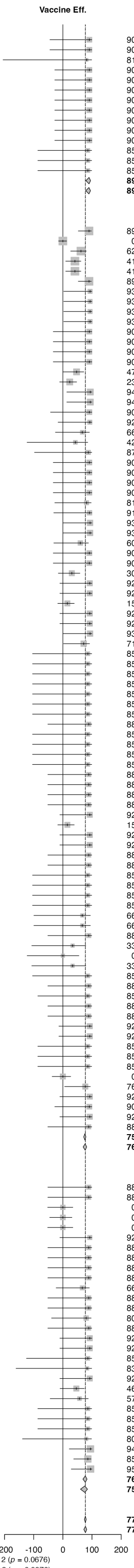

Heterogeneity: $I^2 = 54.3\%$, $\tau^2 = 0.5615$, $p < 0.0001$
Test for subgroup differences (common effect): $\chi^2_2 = 5.39$, df = 2 ($p = 0.0676$)
Test for subgroup differences (random effects): $\chi^2_2 = 4.65$, df = 2 ($p = 0.0976$)

Supplementary Figure 8. Vaccine efficacy of N1-containing seasonal vaccines: subgroup analysis by the time interval from vaccination to challenge.

Note. The time interval from vaccination to viral challenge was categorized as short (≤7 days), medium (8–30 days), and long (≥31 days).

Study	Vaccinated Events	Vaccinated Total	Control Events	Control Total	VE	95%-CI	Weight (common)	Weight (random)
**DPI_group = medium**								
Rockman et al (2013) (1)	2	4	4	4	44.44	[-26.95; 75.69]	4.0%	10.2%
Rockman et al (2013) (11)	0	4	4	4	88.89	[-51.60; 99.19]	4.0%	3.1%
Rockman et al (2013) (12)	0	4	4	4	88.89	[-51.60; 99.19]	4.0%	3.1%
Rockman et al (2013) (14)	0	4	4	4	88.89	[-51.60; 99.19]	4.0%	3.1%
Rockman et al (2013) (2)	0	4	4	4	88.89	[-51.60; 99.19]	4.0%	3.1%
Rockman et al (2013) (3)	0	4	4	4	88.89	[-51.60; 99.19]	4.0%	3.1%
Rockman et al (2013) (4)	0	3	4	4	85.71	[-85.95; 98.90]	3.5%	3.2%
Rockman et al (2013) (6)	0	4	4	4	88.89	[-51.60; 99.19]	4.0%	3.1%
Rockman et al (2013) (7)	0	4	4	4	88.89	[-51.60; 99.19]	4.0%	3.1%
Rockman et al (2013) (8)	5	8	4	4	35.29	[-6.31; 60.62]	5.1%	12.1%
Hatta et al (2017) (1)	1	9	5	9	80.00	[-38.91; 97.12]	4.4%	4.8%
Haynes et al (2009) (1)	0	7	6	7	92.31	[-13.60; 99.48]	5.7%	3.0%
Perrone et al (2009) (1)	0	6	6	6	92.31	[-10.31; 99.46]	5.7%	3.0%
Perrone et al (2009) (2)	3	6	6	6	46.15	[-9.71; 73.57]	5.7%	10.9%
**Common effect model**		**71**		**68**	**77.26**	**[64.51; 85.43]**	**62.0%**	**.**
**Random effects model**					**71.55**	**[50.41; 83.68]**	**.**	**68.9%**
Heterogeneity: $I^2 = 13.5\%$, $\tau^2 = 0.3147$, $p = 0.3059$								
**DPI_group = long**								
Park et al (2014) (1)	0	12	12	12	96.00	[39.53; 99.74]	11.0%	2.9%
Park et al (2014) (3)	12	12	12	12	0.00	[-17.35; 14.79]	11.0%	13.4%
Hatta et al (2017) (2)	1	9	5	9	80.00	[-38.91; 97.12]	4.4%	4.8%
Price et al (2009) (1)	1	6	6	6	76.92	[6.08; 94.33]	5.7%	6.9%
Price et al (2009) (2)	0	6	6	6	92.31	[-10.31; 99.46]	5.7%	3.0%
**Common effect model**		**45**		**45**	**62.79**	**[44.29; 75.15]**	**38.0%**	**.**
**Random effects model**					**74.20**	**[14.28; 92.23]**	**.**	**31.1%**
Heterogeneity: $I^2 = 74.1\%$, $\tau^2 = 1.1063$, $p = 0.0039$								
**Common effect model**		**116**		**113**	**71.77**	**[61.74; 79.16]**	**100.0%**	**.**
**Random effects model**					**72.78**	**[53.99; 83.90]**	**.**	**100.0%**

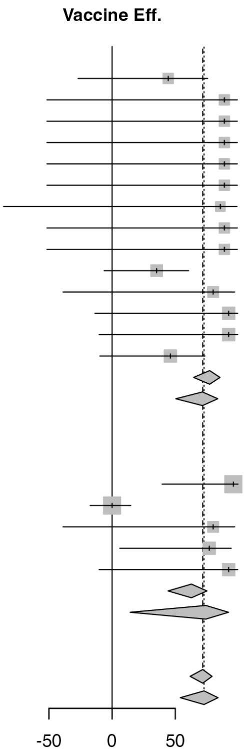

Heterogeneity: $I^2 = 62.1\%$, $\tau^2 = 0.5273$, $p = 0.0002$
Test for subgroup differences (common effect): $\chi^2_1 = 2.58$, df = 1 ($p = 0.1082$)
Test for subgroup differences (random effects): $\chi^2_1 = 0.02$, df = 1 ($p = 0.8851$)

Supplementary Figure 9. Vaccine efficacy in N1-containing seasonal vaccine: subgroup analysis by vaccine platform.

Study	Vaccinated Events	Vaccinated Total	Control Events	Control Total	VE	95%-CI	Weight
**Platform = VLP**							
Haynes et al (2009) (1)	0	7	6	7	92.31	[-13.60; 99.48]	3.0%
Perrone et al (2009) (1)	0	6	6	6	92.31	[-10.31; 99.46]	3.0%
Perrone et al (2009) (2)	3	6	6	6	46.15	[-9.71; 73.57]	10.9%
**Random effects model**		**19**		**19**	**76.47**	**[-5.47; 94.75]**	**16.9%**
Heterogeneity: $I^2 = 43.7\%$, $\tau^2 = 0.8876$, $p = 0.1690$							
**Platform = DNA vaccine**							
Price et al (2009) (1)	1	6	6	6	76.92	[6.08; 94.33]	6.9%
Price et al (2009) (2)	0	6	6	6	92.31	[-10.31; 99.46]	3.0%
**Random effects model**		**12**		**12**	**81.83**	**[37.09; 94.75]**	**9.9%**
Heterogeneity: $I^2 = 0\%$, $\tau^2 = 0$, $p = 0.4744$							
**Platform = Inactivated vaccine**							
Rockman et al (2013) (1)	2	4	4	4	44.44	[-26.95; 75.69]	10.2%
Rockman et al (2013) (11)	0	4	4	4	88.89	[-51.60; 99.19]	3.1%
Rockman et al (2013) (12)	0	4	4	4	88.89	[-51.60; 99.19]	3.1%
Rockman et al (2013) (14)	0	4	4	4	88.89	[-51.60; 99.19]	3.1%
Rockman et al (2013) (2)	0	4	4	4	88.89	[-51.60; 99.19]	3.1%
Rockman et al (2013) (3)	0	4	4	4	88.89	[-51.60; 99.19]	3.1%
Rockman et al (2013) (4)	0	3	4	4	85.71	[-85.95; 98.90]	3.2%
Rockman et al (2013) (6)	0	4	4	4	88.89	[-51.60; 99.19]	3.1%
Rockman et al (2013) (7)	0	4	4	4	88.89	[-51.60; 99.19]	3.1%
Rockman et al (2013) (8)	5	8	4	4	35.29	[-6.31; 60.62]	12.1%
Park et al (2014) (3)	12	12	12	12	0.00	[-17.35; 14.79]	13.4%
**Random effects model**		**55**		**52**	**66.45**	**[33.48; 83.08]**	**60.7%**
Heterogeneity: $I^2 = 58.1\%$, $\tau^2 = 0.5552$, $p = 0.0080$							
**Platform = Live virus**							
Park et al (2014) (1)	0	12	12	12	96.00	[39.53; 99.74]	2.9%
Hatta et al (2017) (1)	1	9	5	9	80.00	[-38.91; 97.12]	4.8%
Hatta et al (2017) (2)	1	9	5	9	80.00	[-38.91; 97.12]	4.8%
**Random effects model**		**30**		**30**	**85.57**	**[50.96; 95.76]**	**12.5%**
Heterogeneity: $I^2 = 0\%$, $\tau^2 = 0$, $p = 0.5841$							
**Random effects model**		**116**		**113**	**72.78**	**[53.99; 83.90]**	**100.0%**
Heterogeneity: $I^2 = 62.1\%$, $\tau^2 = 0.5272$, $p = 0.0002$							
Test for subgroup differences: $\chi^2_3 = 1.74$, df = 3 ($p = 0.6271$)							

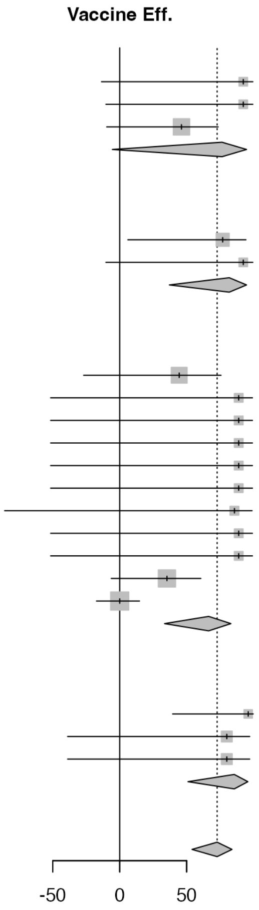

Supplementary Figure 10. Vaccine efficacy in N1-containing seasonal vaccine: subgroup analysis by adding adjuvant or not.

Study	Vaccinated Events	Vaccinated Total	Control Events	Control Total	VE	95%-CI	Weight
**Adjuvant = 0**							
Haynes et al (2009) (1)	0	7	6	7	92.31	[-13.60; 99.48]	3.0%
Perrone et al (2009) (1)	0	6	6	6	92.31	[-10.31; 99.46]	3.0%
Perrone et al (2009) (2)	3	6	6	6	46.15	[-9.71; 73.57]	10.9%
Price et al (2009) (1)	1	6	6	6	76.92	[6.08; 94.33]	6.9%
Price et al (2009) (2)	0	6	6	6	92.31	[-10.31; 99.46]	3.0%
Rockman et al (2013) (1)	2	4	4	4	44.44	[-26.95; 75.69]	10.2%
Park et al (2014) (1)	0	12	12	12	96.00	[39.53; 99.74]	2.9%
Park et al (2014) (3)	12	12	12	12	0.00	[-17.35; 14.79]	13.4%
Hatta et al (2017) (1)	1	9	5	9	80.00	[-38.91; 97.12]	4.8%
Hatta et al (2017) (2)	1	9	5	9	80.00	[-38.91; 97.12]	4.8%
**Random effects model**		**77**		**77**	**67.82**	**[37.18; 83.52]**	**62.9%**
Heterogeneity: I^2 = 67.9%, τ^2 = 0.5453, p = 0.0010							
**Adjuvant = 1**							
Rockman et al (2013) (11)	0	4	4	4	88.89	[-51.60; 99.19]	3.1%
Rockman et al (2013) (12)	0	4	4	4	88.89	[-51.60; 99.19]	3.1%
Rockman et al (2013) (14)	0	4	4	4	88.89	[-51.60; 99.19]	3.1%
Rockman et al (2013) (2)	0	4	4	4	88.89	[-51.60; 99.19]	3.1%
Rockman et al (2013) (3)	0	4	4	4	88.89	[-51.60; 99.19]	3.1%
Rockman et al (2013) (4)	0	3	4	4	85.71	[-85.95; 98.90]	3.2%
Rockman et al (2013) (6)	0	4	4	4	88.89	[-51.60; 99.19]	3.1%
Rockman et al (2013) (7)	0	4	4	4	88.89	[-51.60; 99.19]	3.1%
Rockman et al (2013) (8)	5	8	4	4	35.29	[-6.31; 60.62]	12.1%
**Random effects model**		**39**		**36**	**79.86**	**[52.22; 91.51]**	**37.1%**
Heterogeneity: I^2 = 23.9%, τ^2 = 0.5325, p = 0.2308							
**Random effects model**		**116**		**113**	**72.78**	**[53.99; 83.90]**	**100.0%**
Heterogeneity: I^2 = 62.1%, τ^2 = 0.5272, p = 0.0002							
Test for subgroup differences: χ^2_1 = 0.71, df = 1 (p = 0.4007)							

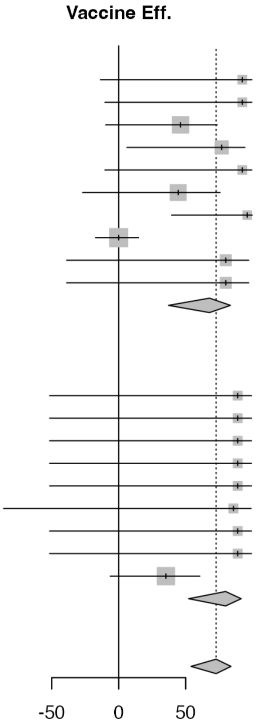

Supplementary Figure 11. Vaccine efficacy in N1-containing seasonal vaccine: subgroup analysis by booster or not.

Study	Vaccinated Events	Vaccinated Total	Control Events	Control Total	VE	95%-CI	Weight
**Booster = Yes**							
Haynes et al (2009) (1)	0	7	6	7	92.31	[-13.60; 99.48]	3.0%
Perrone et al (2009) (1)	0	6	6	6	92.31	[-10.31; 99.46]	3.0%
Perrone et al (2009) (2)	3	6	6	6	46.15	[-9.71; 73.57]	10.9%
Price et al (2009) (1)	1	6	6	6	76.92	[6.08; 94.33]	6.9%
Price et al (2009) (2)	0	6	6	6	92.31	[-10.31; 99.46]	3.0%
Rockman et al (2013) (1)	2	4	4	4	44.44	[-26.95; 75.69]	10.2%
Rockman et al (2013) (11)	0	4	4	4	88.89	[-51.60; 99.19]	3.1%
Rockman et al (2013) (12)	0	4	4	4	88.89	[-51.60; 99.19]	3.1%
Rockman et al (2013) (14)	0	4	4	4	88.89	[-51.60; 99.19]	3.1%
Rockman et al (2013) (2)	0	4	4	4	88.89	[-51.60; 99.19]	3.1%
Rockman et al (2013) (3)	0	4	4	4	88.89	[-51.60; 99.19]	3.1%
Rockman et al (2013) (4)	0	3	4	4	85.71	[-85.95; 98.90]	3.2%
Rockman et al (2013) (6)	0	4	4	4	88.89	[-51.60; 99.19]	3.1%
Rockman et al (2013) (7)	0	4	4	4	88.89	[-51.60; 99.19]	3.1%
Rockman et al (2013) (8)	5	8	4	4	35.29	[-6.31; 60.62]	12.1%
Park et al (2014) (3)	12	12	12	12	0.00	[-17.35; 14.79]	13.4%
Hatta et al (2017) (1)	1	9	5	9	80.00	[-38.91; 97.12]	4.8%
**Random effects model**		**95**		**92**	**69.90**	**[48.71; 82.34]**	**92.3%**
Heterogeneity: $I^2 = 60.6\%$, $\tau^2 = 0.4839$, $p = 0.0006$							
**Booster = No**							
Park et al (2014) (1)	0	12	12	12	96.00	[39.53; 99.74]	2.9%
Hatta et al (2017) (2)	1	9	5	9	80.00	[-38.91; 97.12]	4.8%
**Random effects model**		**21**		**21**	**88.38**	**[43.73; 97.60]**	**7.7%**
Heterogeneity: $I^2 = 0\%$, $\tau^2 = 0$, $p = 0.3444$							
**Random effects model**		**116**		**113**	**72.78**	**[53.99; 83.90]**	**100.0%**
Heterogeneity: $I^2 = 62.1\%$, $\tau^2 = 0.5272$, $p = 0.0002$							
Test for subgroup differences: $\chi^2_1 = 1.25$, df = 1 ($p = 0.2626$)							

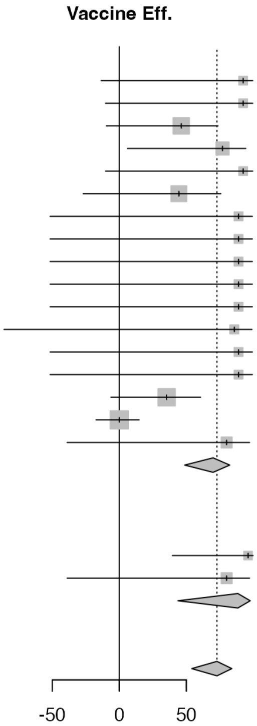

Supplementary Figure 12. Vaccine efficacy in seasonal influenza vaccine containing N1: subgroup analysis by the study year.

Study	Vaccinated Events	Vaccinated Total	Control Events	Control Total	VE	95%-CI	Weight
**period = pre-2009**							
Haynes et al (2009) (1)	0	7	6	7	92.31	[-13.60; 99.48]	3.0%
Perrone et al (2009) (1)	0	6	6	6	92.31	[-10.31; 99.46]	3.0%
Perrone et al (2009) (2)	3	6	6	6	46.15	[-9.71; 73.57]	10.9%
Price et al (2009) (1)	1	6	6	6	76.92	[6.08; 94.33]	6.9%
Price et al (2009) (2)	0	6	6	6	92.31	[-10.31; 99.46]	3.0%
**Random effects model**		**31**		**31**	**76.30**	**[38.71; 90.84]**	**26.8%**
Heterogeneity: $I^2 = 26.6\%$, $\tau^2 = 0.4093$, $p = 0.2443$							
**period = post-2009**							
Rockman et al (2013) (1)	2	4	4	4	44.44	[-26.95; 75.69]	10.2%
Rockman et al (2013) (2)	0	4	4	4	88.89	[-51.60; 99.19]	3.1%
Rockman et al (2013) (3)	0	4	4	4	88.89	[-51.60; 99.19]	3.1%
Rockman et al (2013) (4)	0	3	4	4	85.71	[-85.95; 98.90]	3.2%
Rockman et al (2013) (6)	0	4	4	4	88.89	[-51.60; 99.19]	3.1%
Rockman et al (2013) (7)	0	4	4	4	88.89	[-51.60; 99.19]	3.1%
Rockman et al (2013) (8)	5	8	4	4	35.29	[-6.31; 60.62]	12.1%
Rockman et al (2013) (11)	0	4	4	4	88.89	[-51.60; 99.19]	3.1%
Rockman et al (2013) (12)	0	4	4	4	88.89	[-51.60; 99.19]	3.1%
Rockman et al (2013) (14)	0	4	4	4	88.89	[-51.60; 99.19]	3.1%
Park et al (2014) (1)	0	12	12	12	96.00	[39.53; 99.74]	2.9%
Park et al (2014) (3)	12	12	12	12	0.00	[-17.35; 14.79]	13.4%
Hatta et al (2017) (1)	1	9	5	9	80.00	[-38.91; 97.12]	4.8%
Hatta et al (2017) (2)	1	9	5	9	80.00	[-38.91; 97.12]	4.8%
**Random effects model**		**85**		**82**	**71.96**	**[46.95; 85.18]**	**73.2%**
Heterogeneity: $I^2 = 61\%$, $\tau^2 = 0.6039$, $p = 0.0015$							
**Random effects model**		**116**		**113**	**72.78**	**[53.99; 83.90]**	**100.0%**
Heterogeneity: $I^2 = 62.1\%$, $\tau^2 = 0.5272$, $p = 0.0002$							
Test for subgroup differences: $\chi^2_1 = 0.08$, df = 1 ($p = 0.7735$)							

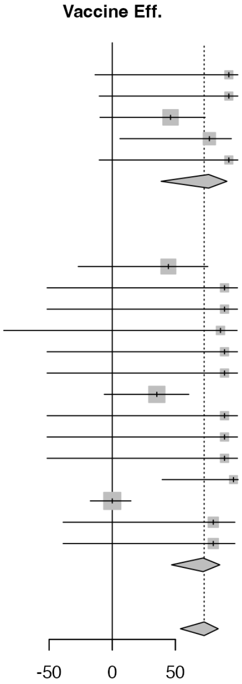

Supplementary Figure 13. Leave-one-out forest plot for seasonal influenza vaccines containing N1 trials.

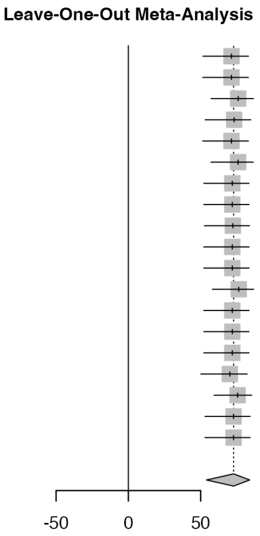

Study	VE	95%-CI	P-value	Tau2	Tau	I2
Omitting Haynes et al (2009) (1)	71.34	[51.51; 83.06]	< 0.0001	0.5028	0.7091	61.8%
Omitting Perrone et al (2009) (1)	71.31	[51.46; 83.04]	< 0.0001	0.5020	0.7085	61.7%
Omitting Perrone et al (2009) (2)	75.94	[57.14; 86.50]	< 0.0001	0.6077	0.7796	63.1%
Omitting Price et al (2009) (1)	73.17	[53.12; 84.65]	< 0.0001	0.5771	0.7597	61.6%
Omitting Price et al (2009) (2)	71.31	[51.46; 83.04]	< 0.0001	0.5020	0.7085	61.7%
Omitting Rockman et al (2013) (1)	75.87	[57.12; 86.42]	< 0.0001	0.6052	0.7779	63.5%
Omitting Rockman et al (2013) (2)	71.88	[52.16; 83.47]	< 0.0001	0.5201	0.7212	62.4%
Omitting Rockman et al (2013) (3)	71.88	[52.16; 83.47]	< 0.0001	0.5201	0.7212	62.4%
Omitting Rockman et al (2013) (4)	72.26	[52.64; 83.75]	< 0.0001	0.5317	0.7292	62.8%
Omitting Rockman et al (2013) (6)	71.88	[52.16; 83.47]	< 0.0001	0.5201	0.7212	62.4%
Omitting Rockman et al (2013) (7)	71.88	[52.16; 83.47]	< 0.0001	0.5201	0.7212	62.4%
Omitting Rockman et al (2013) (8)	76.35	[58.12; 86.65]	< 0.0001	0.5708	0.7555	63.4%
Omitting Rockman et al (2013) (11)	71.88	[52.16; 83.47]	< 0.0001	0.5201	0.7212	62.4%
Omitting Rockman et al (2013) (12)	71.88	[52.16; 83.47]	< 0.0001	0.5201	0.7212	62.4%
Omitting Rockman et al (2013) (14)	71.88	[52.16; 83.47]	< 0.0001	0.5201	0.7212	62.4%
Omitting Park et al (2014) (1)	70.20	[50.13; 82.19]	< 0.0001	0.4665	0.6830	60.2%
Omitting Park et al (2014) (3)	75.55	[59.25; 85.33]	< 0.0001	0.3449	0.5873	17.8%
Omitting Hatta et al (2017) (1)	72.74	[52.97; 84.20]	< 0.0001	0.5537	0.7441	62.6%
Omitting Hatta et al (2017) (2)	72.74	[52.97; 84.20]	< 0.0001	0.5537	0.7441	62.6%
**Random effects model**	**72.78**	**[53.99; 83.90]**	**< 0.0001**	**0.5273**	**0.7261**	**62.1%**

Supplementary Figure 14. Leave-one-out forest plot for H5N1 vaccines trials achieving seroprotection.

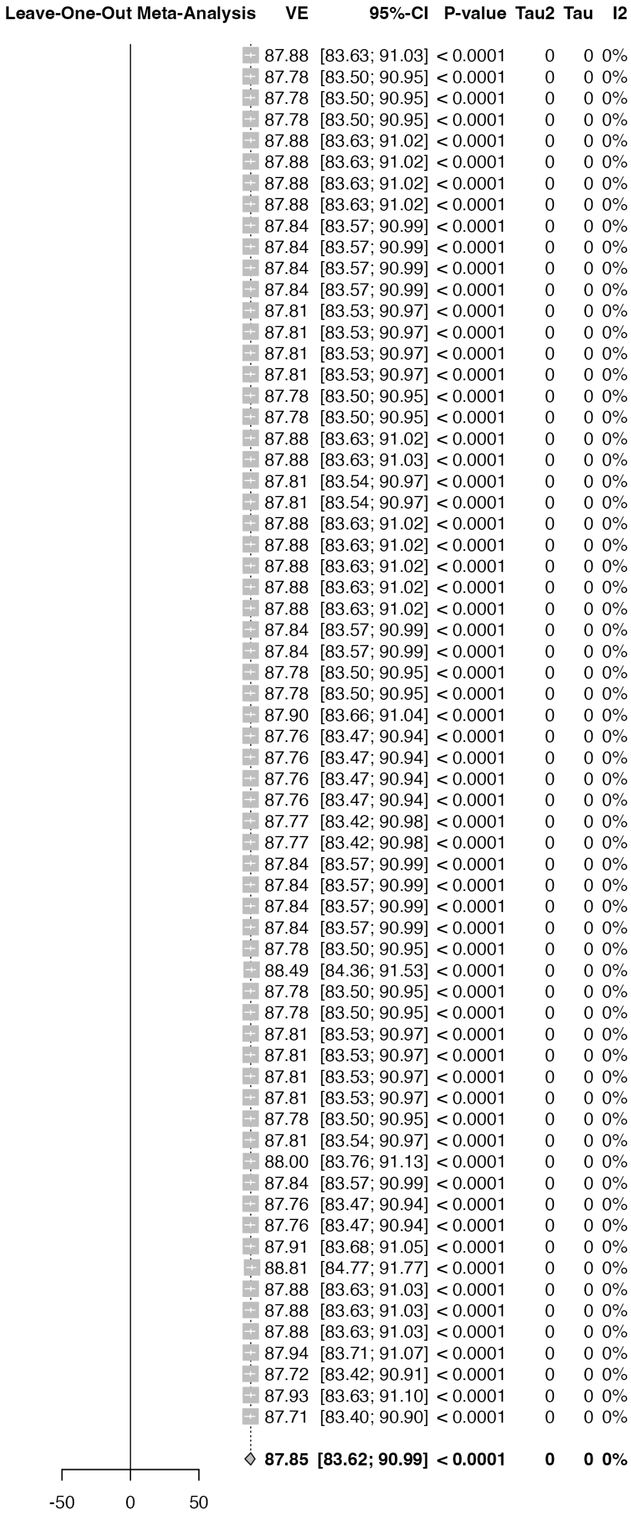

Study	VE	95%-CI	P-value	Tau2	Tau	I2
Omitting Hoffmann et al (2005) (3)	87.88	[83.63; 91.03]	< 0.0001	0	0	0%
Omitting Baras et al (2008) (3)	87.78	[83.50; 90.95]	< 0.0001	0	0	0%
Omitting Lalor et al (2008) (2)	87.78	[83.50; 90.95]	< 0.0001	0	0	0%
Omitting Lalor et al (2008) (3)	87.78	[83.50; 90.95]	< 0.0001	0	0	0%
Omitting Mahmood et al (2008) (2)	87.88	[83.63; 91.02]	< 0.0001	0	0	0%
Omitting Mahmood et al (2008) (3)	87.88	[83.63; 91.02]	< 0.0001	0	0	0%
Omitting Mahmood et al (2008) (4)	87.88	[83.63; 91.02]	< 0.0001	0	0	0%
Omitting Mahmood et al (2008) (5)	87.88	[83.63; 91.02]	< 0.0001	0	0	0%
Omitting Mahmood et al (2008) (6)	87.84	[83.57; 90.99]	< 0.0001	0	0	0%
Omitting Mahmood et al (2008) (7)	87.84	[83.57; 90.99]	< 0.0001	0	0	0%
Omitting Mahmood et al (2008) (8)	87.84	[83.57; 90.99]	< 0.0001	0	0	0%
Omitting Mahmood et al (2008) (10)	87.84	[83.57; 90.99]	< 0.0001	0	0	0%
Omitting Forrest et al (2009) (1)	87.81	[83.53; 90.97]	< 0.0001	0	0	0%
Omitting Forrest et al (2009) (2)	87.81	[83.53; 90.97]	< 0.0001	0	0	0%
Omitting Forrest et al (2009) (4)	87.81	[83.53; 90.97]	< 0.0001	0	0	0%
Omitting Forrest et al (2009) (5)	87.81	[83.53; 90.97]	< 0.0001	0	0	0%
Omitting Haynes et al (2009) (2)	87.78	[83.50; 90.95]	< 0.0001	0	0	0%
Omitting Haynes et al (2009) (3)	87.78	[83.50; 90.95]	< 0.0001	0	0	0%
Omitting Middleton et al (2009) (4)	87.88	[83.63; 91.02]	< 0.0001	0	0	0%
Omitting Middleton et al (2009) (6)	87.88	[83.63; 91.03]	< 0.0001	0	0	0%
Omitting Shoji et al (2009) (1)	87.81	[83.54; 90.97]	< 0.0001	0	0	0%
Omitting Shoji et al (2009) (2)	87.81	[83.54; 90.97]	< 0.0001	0	0	0%
Omitting Song et al (2009) (2)	87.88	[83.63; 91.02]	< 0.0001	0	0	0%
Omitting Song et al (2009) (3)	87.88	[83.63; 91.02]	< 0.0001	0	0	0%
Omitting Song et al (2009) (4)	87.88	[83.63; 91.02]	< 0.0001	0	0	0%
Omitting Song et al (2009) (5)	87.88	[83.63; 91.02]	< 0.0001	0	0	0%
Omitting Song et al (2009) (6)	87.88	[83.63; 91.02]	< 0.0001	0	0	0%
Omitting Rao et al (2010) (1)	87.84	[83.57; 90.99]	< 0.0001	0	0	0%
Omitting Rao et al (2010) (2)	87.84	[83.57; 90.99]	< 0.0001	0	0	0%
Omitting Gustin et al (2011) (1)	87.78	[83.50; 90.95]	< 0.0001	0	0	0%
Omitting Gustin et al (2011) (2)	87.78	[83.50; 90.95]	< 0.0001	0	0	0%
Omitting Gustin et al (2011) (4)	87.90	[83.66; 91.04]	< 0.0001	0	0	0%
Omitting Layton et al (2011) (1)	87.76	[83.47; 90.94]	< 0.0001	0	0	0%
Omitting Layton et al (2011) (2)	87.76	[83.47; 90.94]	< 0.0001	0	0	0%
Omitting Layton et al (2011) (3)	87.76	[83.47; 90.94]	< 0.0001	0	0	0%
Omitting Layton et al (2011) (4)	87.76	[83.47; 90.94]	< 0.0001	0	0	0%
Omitting Layton et al (2011) (9)	87.77	[83.42; 90.98]	< 0.0001	0	0	0%
Omitting Layton et al (2011) (10)	87.77	[83.42; 90.98]	< 0.0001	0	0	0%
Omitting Ducatez et al (2012) (1)	87.84	[83.57; 90.99]	< 0.0001	0	0	0%
Omitting Ducatez et al (2012) (2)	87.84	[83.57; 90.99]	< 0.0001	0	0	0%
Omitting Ducatez et al (2012) (3)	87.84	[83.57; 90.99]	< 0.0001	0	0	0%
Omitting Ducatez et al (2012) (4)	87.84	[83.57; 90.99]	< 0.0001	0	0	0%
Omitting Scallan et al (2012) (1)	87.78	[83.50; 90.95]	< 0.0001	0	0	0%
Omitting Scallan et al (2012) (2)	88.49	[84.36; 91.53]	< 0.0001	0	0	0%
Omitting Verma et al (2012) (2)	87.78	[83.50; 90.95]	< 0.0001	0	0	0%
Omitting Verma et al (2012) (5)	87.78	[83.50; 90.95]	< 0.0001	0	0	0%
Omitting Mann et al (2014) (3)	87.81	[83.53; 90.97]	< 0.0001	0	0	0%
Omitting Cox et al (2015) (2)	87.81	[83.53; 90.97]	< 0.0001	0	0	0%
Omitting Cox et al (2015) (3)	87.81	[83.53; 90.97]	< 0.0001	0	0	0%
Omitting Cox et al (2015) (4)	87.81	[83.53; 90.97]	< 0.0001	0	0	0%
Omitting Liu et al (2016) (1)	87.78	[83.50; 90.95]	< 0.0001	0	0	0%
Omitting Hatta et al (2017) (3)	87.81	[83.54; 90.97]	< 0.0001	0	0	0%
Omitting Hatta et al (2017) (4)	88.00	[83.76; 91.13]	< 0.0001	0	0	0%
Omitting Wong et al (2017) (3)	87.84	[83.57; 90.99]	< 0.0001	0	0	0%
Omitting Smith et al (2019) (3)	87.76	[83.47; 90.94]	< 0.0001	0	0	0%
Omitting Wang et al (2019) (1)	87.76	[83.47; 90.94]	< 0.0001	0	0	0%
Omitting Schön et al (2020) (1)	87.91	[83.68; 91.05]	< 0.0001	0	0	0%
Omitting Hatta et al (2024) (1)	88.81	[84.77; 91.77]	< 0.0001	0	0	0%
Omitting Hatta et al (2024) (2)	87.88	[83.63; 91.03]	< 0.0001	0	0	0%
Omitting Hatta et al (2024) (3)	87.88	[83.63; 91.03]	< 0.0001	0	0	0%
Omitting Hatta et al (2024) (4)	87.88	[83.63; 91.03]	< 0.0001	0	0	0%
Omitting Hatta et al (2024) (5)	87.94	[83.71; 91.07]	< 0.0001	0	0	0%
Omitting Hatta et al (2024) (6)	87.72	[83.42; 90.91]	< 0.0001	0	0	0%
Omitting Hatta et al (2024) (7)	87.93	[83.63; 91.10]	< 0.0001	0	0	0%
Omitting Hatta et al (2024) (8)	87.71	[83.40; 90.90]	< 0.0001	0	0	0%
**Random effects model**	**87.85**	**[83.62; 90.99]**	**< 0.0001**	**0**	**0**	**0%**

Supplementary Figure 15. Quality assessment of each study according to Collaborative Approach to Meta-analysis and Review of Animal Data in Experimental Studies (CAMARADES) checklist.

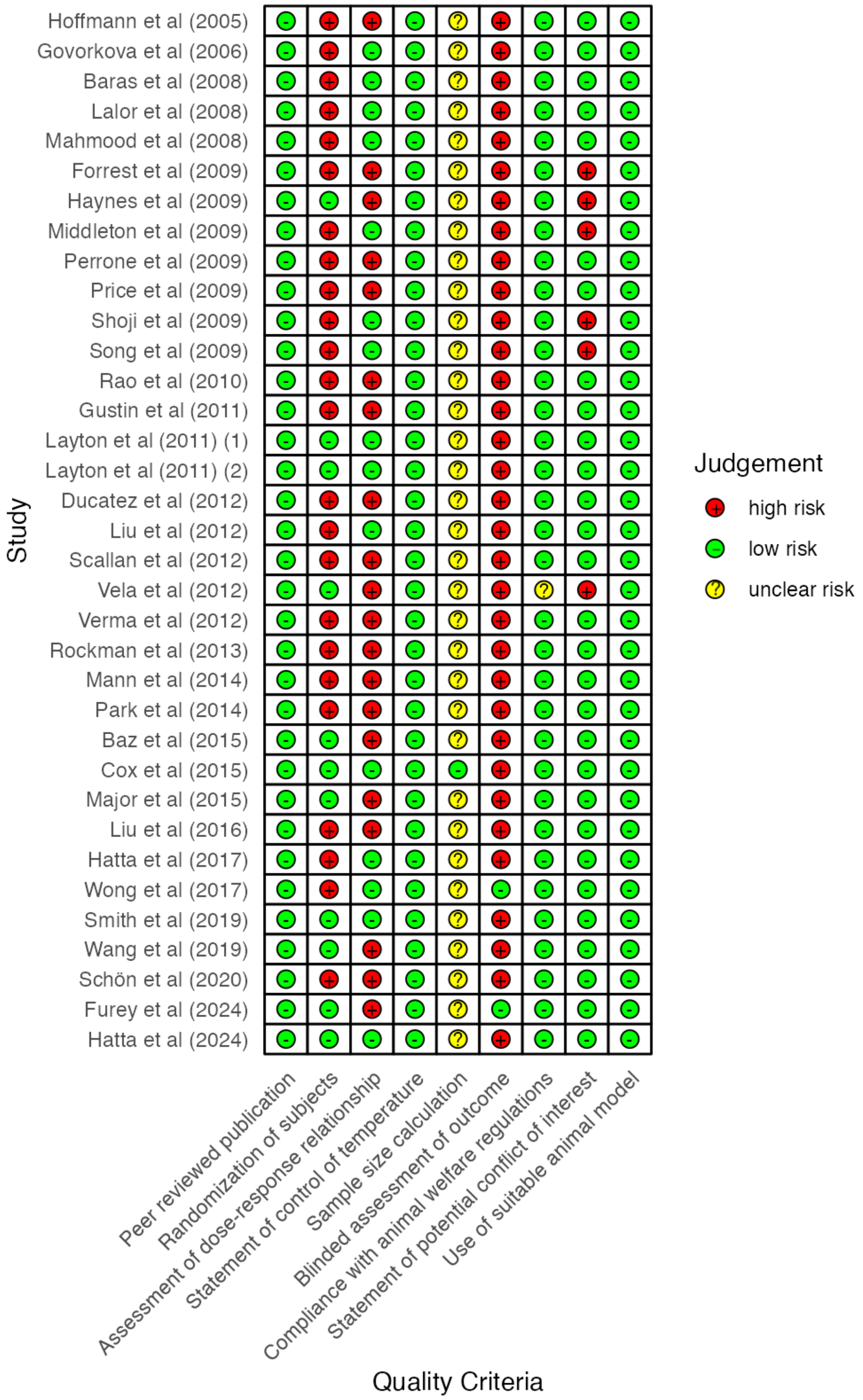

Supplementary Figure 16. Summary of quality assessment.

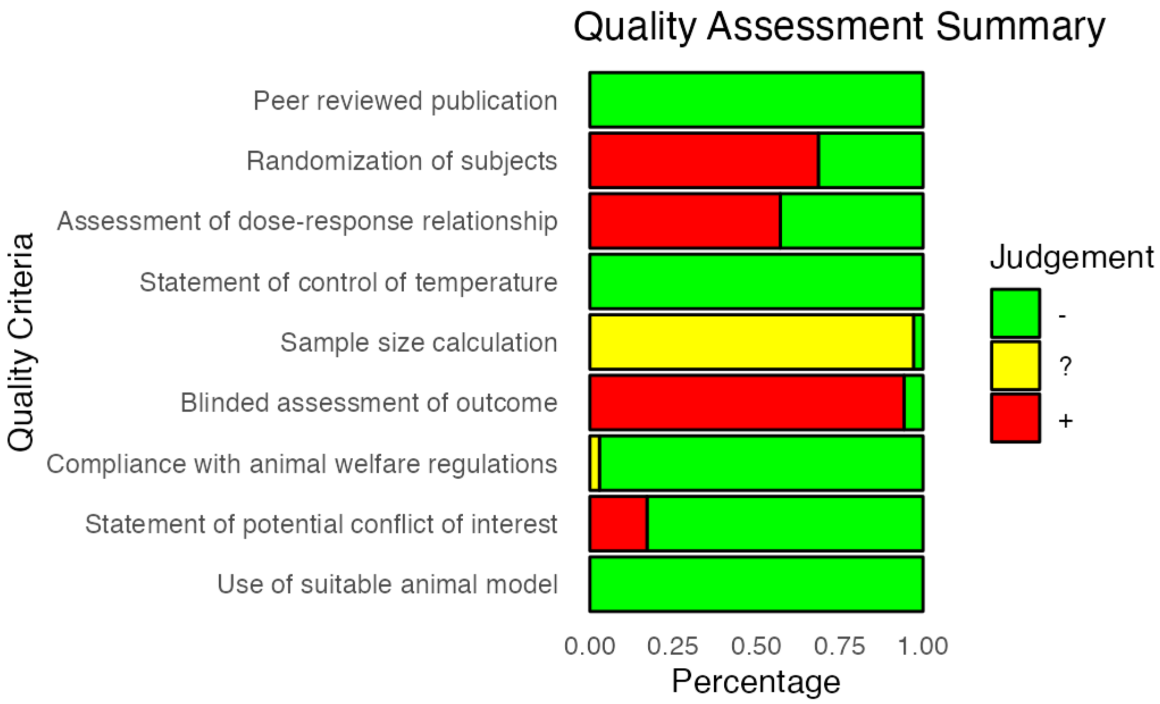

Supplementary Figure 17. Publication bias assessment of H5N1 influenza vaccine trials with seroprotection by sample size category.

Study	Vaccinated Events	Vaccinated Total	Control Events	Control Total	VE	95%-CI	Weight
**number = small**							
Hoffmann et al (2005) (3)	0	3	3	3	85.71	[-85.95; 98.90]	1.3%
Mahmood et al (2008) (10)	0	4	4	4	88.89	[-51.60; 99.19]	1.3%
Mahmood et al (2008) (2)	0	4	3	4	85.71	[-104.14; 99.00]	1.2%
Mahmood et al (2008) (3)	0	4	3	4	85.71	[-104.14; 99.00]	1.2%
Mahmood et al (2008) (4)	0	4	3	4	85.71	[-104.14; 99.00]	1.2%
Mahmood et al (2008) (5)	0	4	3	4	85.71	[-104.14; 99.00]	1.2%
Mahmood et al (2008) (6)	0	4	4	4	88.89	[-51.60; 99.19]	1.3%
Mahmood et al (2008) (7)	0	4	4	4	88.89	[-51.60; 99.19]	1.3%
Mahmood et al (2008) (8)	0	4	4	4	88.89	[-51.60; 99.19]	1.3%
Middleton et al (2009) (4)	0	4	3	4	85.71	[-104.14; 99.00]	1.2%
Middleton et al (2009) (6)	0	3	4	4	85.71	[-85.95; 98.90]	1.3%
Song et al (2009) (2)	0	4	3	4	85.71	[-104.14; 99.00]	1.2%
Song et al (2009) (3)	0	4	3	4	85.71	[-104.14; 99.00]	1.2%
Song et al (2009) (4)	0	4	3	4	85.71	[-104.14; 99.00]	1.2%
Song et al (2009) (5)	0	4	3	4	85.71	[-104.14; 99.00]	1.2%
Song et al (2009) (6)	0	4	3	4	85.71	[-104.14; 99.00]	1.2%
Rao et al (2010) (1)	0	4	4	4	88.89	[-51.60; 99.19]	1.3%
Rao et al (2010) (2)	0	4	4	4	88.89	[-51.60; 99.19]	1.3%
Layton et al (2011) (1)	0	7	3	3	93.33	[2.98; 99.54]	1.2%
Layton et al (2011) (2)	0	7	3	3	93.33	[2.98; 99.54]	1.2%
Layton et al (2011) (3)	0	7	3	3	93.33	[2.98; 99.54]	1.2%
Layton et al (2011) (4)	0	7	3	3	93.33	[2.98; 99.54]	1.2%
Ducatez et al (2012) (1)	0	4	4	4	88.89	[-51.60; 99.19]	1.3%
Ducatez et al (2012) (2)	0	4	4	4	88.89	[-51.60; 99.19]	1.3%
Ducatez et al (2012) (3)	0	4	4	4	88.89	[-51.60; 99.19]	1.3%
Ducatez et al (2012) (4)	0	4	4	4	88.89	[-51.60; 99.19]	1.3%
Scallan et al (2012) (1)	0	8	3	4	92.44	[-16.43; 99.51]	1.2%
Scallan et al (2012) (2)	2	8	3	4	66.67	[-25.64; 91.16]	5.0%
Wong et al (2017) (3)	0	4	4	4	88.89	[-51.60; 99.19]	1.3%
Schön et al (2020) (1)	0	4	1	2	81.48	[-205.27; 98.88]	1.1%
Furey et al (2024) (1)	0	4	4	4	88.89	[-51.60; 99.19]	1.3%
Hatta et al (2024) (1)	1	3	3	3	57.14	[-43.68; 87.22]	6.0%
Hatta et al (2024) (2)	0	3	3	3	85.71	[-85.95; 98.90]	1.3%
Hatta et al (2024) (3)	0	3	3	3	85.71	[-85.95; 98.90]	1.3%
Hatta et al (2024) (4)	0	3	3	3	85.71	[-85.95; 98.90]	1.3%
Hatta et al (2024) (5)	0	2	3	3	80.00	[-138.63; 98.32]	1.4%
**Random effects model**		**156**		**132**	**84.84**	**[77.31; 89.86]**	**54.2%**
Heterogeneity: $I^2 = 0\%$, $\tau^2 = 0$, $p = 1.0000$							
**number = medium**							
Baras et al (2008) (3)	0	6	6	6	92.31	[-10.31; 99.46]	1.2%
Lalor et al (2008) (2)	0	6	6	6	92.31	[-10.31; 99.46]	1.2%
Lalor et al (2008) (3)	0	6	6	6	92.31	[-10.31; 99.46]	1.2%
Forrest et al (2009) (1)	0	5	5	5	90.91	[-27.75; 99.35]	1.3%
Forrest et al (2009) (2)	0	5	5	5	90.91	[-27.75; 99.35]	1.3%
Forrest et al (2009) (4)	0	5	5	5	90.91	[-27.75; 99.35]	1.3%
Forrest et al (2009) (5)	0	5	5	5	90.91	[-27.75; 99.35]	1.3%
Haynes et al (2009) (2)	0	7	6	7	92.31	[-13.60; 99.48]	1.2%
Haynes et al (2009) (3)	0	7	6	7	92.31	[-13.60; 99.48]	1.2%
Gustin et al (2011) (1)	0	6	6	6	92.31	[-10.31; 99.46]	1.2%
Gustin et al (2011) (2)	0	6	6	6	92.31	[-10.31; 99.46]	1.2%
Gustin et al (2011) (4)	0	5	3	6	83.12	[-160.68; 98.91]	1.2%
Verma et al (2012) (2)	0	6	6	6	92.31	[-10.31; 99.46]	1.2%
Verma et al (2012) (5)	0	6	6	6	92.31	[-10.31; 99.46]	1.2%
Mann et al (2014) (3)	0	6	5	6	90.91	[-33.01; 99.38]	1.2%
Cox et al (2015) (2)	0	6	5	6	90.91	[-33.01; 99.38]	1.2%
Cox et al (2015) (3)	0	6	5	6	90.91	[-33.01; 99.38]	1.2%
Cox et al (2015) (4)	0	6	5	6	90.91	[-33.01; 99.38]	1.2%
Liu et al (2016) (1)	0	6	6	6	92.31	[-10.31; 99.46]	1.2%
Hatta et al (2017) (3)	0	9	5	9	90.91	[-42.47; 99.42]	1.2%
Hatta et al (2017) (4)	1	9	5	9	80.00	[-38.91; 97.12]	2.3%
Smith et al (2019) (3)	0	8	7	8	93.33	[0.78; 99.55]	1.2%
Wang et al (2019) (1)	0	8	7	8	93.33	[0.78; 99.55]	1.2%
**Random effects model**		**145**		**146**	**90.98**	**[84.41; 94.78]**	**29.4%**
Heterogeneity: $I^2 = 0\%$, $\tau^2 = 0$, $p = 1.0000$							
**number = large**							
Shoji et al (2009) (1)	0	10	5	10	90.91	[-44.47; 99.43]	1.2%
Shoji et al (2009) (2)	0	10	5	10	90.91	[-44.47; 99.43]	1.2%
Layton et al (2011) (10)	1	14	14	14	89.66	[52.92; 97.73]	3.8%
Layton et al (2011) (9)	1	14	14	14	89.66	[52.92; 97.73]	3.8%
Hatta et al (2024) (6)	0	9	10	10	94.74	[21.85; 99.65]	1.2%
Hatta et al (2024) (7)	1	10	10	10	85.71	[37.14; 96.75]	4.0%
Hatta et al (2024) (8)	0	10	10	10	95.24	[28.79; 99.68]	1.2%
**Random effects model**		**77**		**78**	**90.12**	**[79.44; 95.25]**	**16.4%**
Heterogeneity: $I^2 = 0\%$, $\tau^2 = 0$, $p = 0.9936$							
**Random effects model**		**378**		**356**	**87.87**	**[83.67; 90.98]**	**100.0%**
Heterogeneity: $I^2 = 0.0\%$, $\tau^2 = 0$, $p = 1.0000$							
Test for subgroup differences: $\chi^2_2 = 2.61$, df = 2 ($p = 0.2713$)							

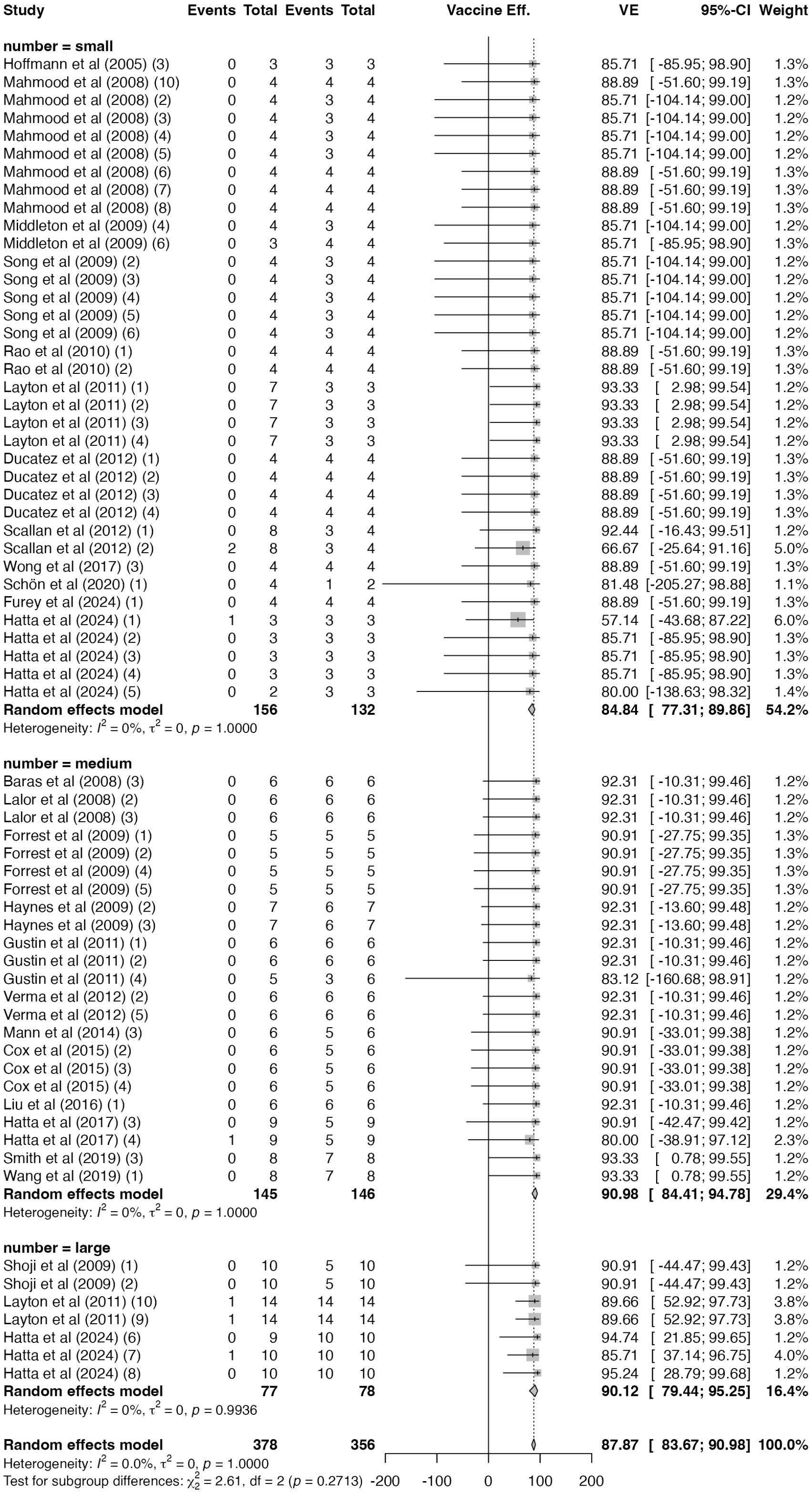

Supplementary Figure 18. Publication bias assessment of H5N1 influenza vaccine trials without seroprotection by sample size category.

Study	Vaccinated Events	Vaccinated Total	Control Events	Control Total	VE	95%-CI	Weight
**number = small**							
Hoffmann et al (2005) (1)	0	3	3	3	85.71	[-85.95; 98.90]	0.8%
Hoffmann et al (2005) (2)	0	3	3	3	85.71	[-85.95; 98.90]	0.8%
Govorkova et al (2006) (1)	0	3	3	3	85.71	[-85.95; 98.90]	0.8%
Govorkova et al (2006) (2)	0	3	3	3	85.71	[-85.95; 98.90]	0.8%
Govorkova et al (2006) (3)	0	3	3	3	85.71	[-85.95; 98.90]	0.8%
Mahmood et al (2008) (1)	0	4	3	4	85.71	[-104.14; 99.00]	0.7%
Mahmood et al (2008) (9)	0	4	4	4	88.89	[-51.60; 99.19]	0.7%
Middleton et al (2009) (1)	2	4	3	4	33.33	[-106.70; 78.50]	2.2%
Middleton et al (2009) (2)	3	4	3	4	0.00	[-122.59; 55.07]	2.8%
Middleton et al (2009) (3)	2	4	3	4	33.33	[-106.70; 78.50]	2.2%
Middleton et al (2009) (5)	0	4	4	4	88.89	[-51.60; 99.19]	0.7%
Middleton et al (2009) (7)	0	4	4	4	88.89	[-51.60; 99.19]	0.7%
Middleton et al (2009) (8)	0	4	4	4	88.89	[-51.60; 99.19]	0.7%
Middleton et al (2009) (9)	0	4	4	4	88.89	[-51.60; 99.19]	0.7%
Middleton et al (2009) (10)	0	4	4	4	88.89	[-51.60; 99.19]	0.7%
Middleton et al (2009) (11)	0	4	4	4	88.89	[-51.60; 99.19]	0.7%
Middleton et al (2009) (12)	0	4	3	4	85.71	[-104.14; 99.00]	0.7%
Middleton et al (2009) (13)	0	4	3	4	85.71	[-104.14; 99.00]	0.7%
Middleton et al (2009) (14)	0	4	3	4	85.71	[-104.14; 99.00]	0.7%
Middleton et al (2009) (15)	0	4	3	4	85.71	[-104.14; 99.00]	0.7%
Middleton et al (2009) (16)	1	4	3	4	66.67	[-99.49; 94.43]	1.3%
Middleton et al (2009) (17)	1	4	3	4	66.67	[-99.49; 94.43]	1.3%
Song et al (2009) (1)	0	4	3	4	85.71	[-104.14; 99.00]	0.7%
Layton et al (2011) (5)	0	6	3	3	92.31	[-10.31; 99.46]	0.7%
Baz et al (2015) (1)	0	4	4	4	88.89	[-51.60; 99.19]	0.7%
Wong et al (2017) (1)	1	4	4	4	66.67	[-23.13; 90.98]	1.9%
Wong et al (2017) (2)	0	4	4	4	88.89	[-51.60; 99.19]	0.7%
**Random effects model**		**105**		**102**	**74.67**	**[60.33; 83.83]**	**26.9%**
Heterogeneity: $I^2 = 0\%$, $\tau^2 = 0.2681$, $p = 0.6992$							
**number = medium**							
Baras et al (2008) (1)	6	6	6	6	0.00	[-36.87; 26.94]	3.8%
Baras et al (2008) (2)	1	6	6	6	76.92	[6.08; 94.33]	1.7%
Baras et al (2008) (4)	0	5	6	6	90.91	[-27.75; 99.35]	0.7%
Baras et al (2008) (5)	0	6	6	6	92.31	[-10.31; 99.46]	0.7%
Lalor et al (2008) (1)	5	6	6	6	15.38	[-17.44; 39.03]	3.7%
Lalor et al (2008) (4)	0	6	6	6	92.31	[-10.31; 99.46]	0.7%
Forrest et al (2009) (3)	0	5	5	5	90.91	[-27.75; 99.35]	0.7%
Forrest et al (2009) (6)	0	5	5	5	90.91	[-27.75; 99.35]	0.7%
Forrest et al (2009) (7)	0	5	5	5	90.91	[-27.75; 99.35]	0.7%
Forrest et al (2009) (8)	0	5	5	5	90.91	[-27.75; 99.35]	0.7%
Rao et al (2010) (3)	4	4	5	5	0.00	[-52.22; 34.31]	3.6%
Rao et al (2010) (4)	5	5	5	5	0.00	[-45.32; 31.19]	3.7%
Rao et al (2010) (5)	4	4	5	5	0.00	[-52.22; 34.31]	3.6%
Gustin et al (2011) (3)	0	6	3	6	85.71	[-124.93; 99.09]	0.7%
Liu et al (2012) (1)	0	6	5	6	90.91	[-33.01; 99.38]	0.7%
Liu et al (2012) (2)	0	6	5	6	90.91	[-33.01; 99.38]	0.7%
Liu et al (2012) (3)	0	6	5	6	90.91	[-33.01; 99.38]	0.7%
Liu et al (2012) (4)	0	6	5	6	90.91	[-33.01; 99.38]	0.7%
Vela et al (2012) (1)	4	8	8	8	47.06	[0.22; 71.91]	3.2%
Vela et al (2012) (2)	6	8	8	8	23.53	[-11.03; 47.33]	3.7%
Vela et al (2012) (3)	0	8	8	8	94.12	[13.43; 99.60]	0.7%
Vela et al (2012) (4)	0	8	8	8	94.12	[13.43; 99.60]	0.7%
Verma et al (2012) (1)	4	6	6	6	30.77	[-15.58; 58.53]	3.4%
Verma et al (2012) (3)	0	6	6	6	92.31	[-10.31; 99.46]	0.7%
Verma et al (2012) (4)	5	6	6	6	15.38	[-17.44; 39.03]	3.7%
Verma et al (2012) (6)	0	6	6	6	92.31	[-10.31; 99.46]	0.7%
Mann et al (2014) (1)	2	6	5	6	60.00	[-31.07; 87.79]	2.1%
Mann et al (2014) (2)	0	6	5	6	90.91	[-33.01; 99.38]	0.7%
Cox et al (2015) (1)	0	6	5	6	90.91	[-33.01; 99.38]	0.7%
Major et al (2015) (1)	2	7	4	8	42.86	[-122.85; 85.35]	1.8%
Major et al (2015) (2)	0	7	4	8	87.41	[-97.34; 99.20]	0.7%
Liu et al (2016) (2)	3	6	6	6	46.15	[-9.71; 73.57]	3.0%
Smith et al (2019) (1)	0	8	4	6	91.50	[-31.25; 99.45]	0.7%
Smith et al (2019) (2)	1	8	4	6	81.25	[-27.73; 97.25]	1.2%
Smith et al (2019) (4)	0	8	7	8	93.33	[0.78; 99.55]	0.7%
Wang et al (2019) (2)	2	8	7	8	71.43	[2.40; 91.64]	2.0%
**Random effects model**		**224**		**225**	**62.26**	**[46.66; 73.30]**	**58.9%**
Heterogeneity: $I^2 = 52.5\%$, $\tau^2 = 0.4954$, $p = 0.0001$							
**number = large**							
Layton et al (2011) (6)	5	14	14	14	62.07	[26.73; 80.36]	3.1%
Layton et al (2011) (7)	8	14	14	14	41.38	[9.66; 61.96]	3.6%
Layton et al (2011) (8)	8	14	14	14	41.38	[9.66; 61.96]	3.6%
Layton et al (2011) (11)	14	14	14	14	0.00	[-14.75; 12.85]	4.0%
**Random effects model**		**56**		**56**	**35.30**	**[3.36; 56.68]**	**14.2%**
Heterogeneity: $I^2 = 81.7\%$, $\tau^2 = 0.1219$, $p = 0.0010$							
**Random effects model**		**385**		**383**	**62.73**	**[52.30; 70.88]**	**100.0%**
Heterogeneity: $I^2 = 52.3\%$, $\tau^2 = 0.3955$, $p < 0.0001$							
Test for subgroup differences: $\chi^2_2 = 9.60$, df = 2 ($p = 0.0082$)							

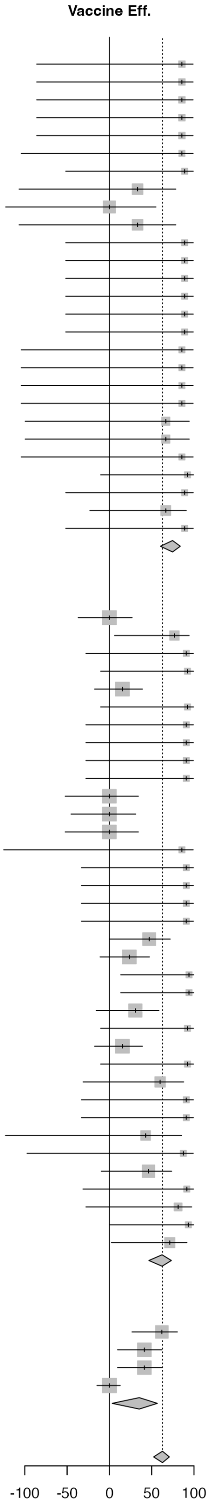

Supplementary Figure 19. Summary of findings.

Summary of findings:

Comparison of Mortality in Ferrets Vaccinated Versus Placebo Following Lethal Challenge Tests

Patient or population: survival after lethal challenging test
Setting:
Intervention: Vaccinated ferrets
Comparison: placebo

Outcomes	Anticipated absolute effects* (95% CI)		Relative effect (95% CI)	№ of participants (studies)	Certainty of the evidence (GRADE)	Comments
	**Risk with placebo**	**Risk with Vaccinated ferrets**				
Mortality (H5N1 vaccination)	294 per 1,000	**227 per 1,000** (211 to 240)	**Vaccine efficacy 0.7737** (0.7186 to 0.8180)	1494 (132 RCTs)	⨁⨁⨁◯ Moderate	Vaccinated ferrets result in a large reduction in mortality. Low heterogeneity (I^2=54.3%) existed between studies,
Mortality (H5N1 vaccination achieving seroprotection)	460 per 1,000	**404 per 1,000** (385 to 419)	**Vaccine efficacy 0.8787** (0.8367 to 0.9098)	726 (65 RCTs)	⨁⨁⨁⨁ High	Vaccinated ferrets result in a large reduction in mortality. Low heterogeneity (I^2=0%) existed between studies,
Mortality (H5N1 vaccination not achieving seroprotection)	141 per 1,000	**88 per 1,000** (74 to 100)	**Vaccine efficacy 0.6273** (0.5230 to 0.7088)	768 (67 RCTs)	⨁⨁⨁◯ Moderate	Vaccinated ferrets result in a large reduction in mortality. Low heterogeneity (I^2=52.3%) existed between studies,
Mortality (seasonal influenza vaccination containing N1)	655 per 1,000	**477 per 1,000** (354 to 549)	**Vaccine efficacy 0.7278** (0.5399 to 0.8390)	229 (19 RCTs)	⨁⨁⨁◯ Moderate	Vaccinated ferrets likely reduce mortality. High heterogeneity (I^2=62.1%) existed between studies, which may be due to the varying effects of the vaccines.

***The risk in the intervention group** (and its 95% confidence interval) is based on the assumed risk in the comparison group and the **relative effect** of the intervention (and its 95% CI).

CI: confidence interval

GRADE Working Group grades of evidence
High certainty: we are very confident that the true effect lies close to that of the estimate of the effect.
Moderate certainty: we are moderately confident in the effect estimate: the true effect is likely to be close to the estimate of the effect, but there is a possibility that it is substantially different.
Low certainty: our confidence in the effect estimate is limited: the true effect may be substantially different from the estimate of the effect.
Very low certainty: we have very little confidence in the effect estimate: the true effect is likely to be substantially different from the estimate of effect.
