# Supplementary material for: Cross-protection against highly pathogenic avian influenza H5N1 virus from seasonal influenza vaccines: a systematic review and meta-analysis of ferret studies
Source: Emerg Microbes Infect. 2026 Apr 15;15(1):2654278. doi: 10.1080/22221751.2026.2654278 (PMC13084842; doi:10.1080/22221751.2026.2654278)

| ICMJE DISCLOSURE FORM | |
| --- | --- |
| **Date:** | 12/10/2025 |
| **Your Name:** | Chi-Tai Fang |
| **Manuscript Title:** | Cross-protection against highly pathogenic avian influenza H5N1 from seasonal influenza vaccination: a systematic review and meta-analysis of ferret studies |
| **Manuscript Number (if known):** | Click or tap here to enter text. |
| In the interest of transparency, we ask you to disclose all relationships/activities/interests listed below that are related to the content of your manuscript. “Related” means any relation with for-profit or not-for-profit third parties whose interests may be affected by the content of the manuscript. Disclosure represents a commitment to transparency and does not necessarily indicate a bias. If you are in doubt about whether to list a relationship/activity/interest, it is preferable that you do so.  The author’s relationships/activities/interests should be defined broadly. For example, if your manuscript pertains to the epidemiology of hypertension, you should declare all relationships with manufacturers of antihypertensive medication, even if that medication is not mentioned in the manuscript.  In item #1 below, report all support for the work reported in this manuscript without time limit. For all other items, the time frame for disclosure is the past 36 months. | |

|  | | | **Name all entities with whom you have this relationship or indicate none (add rows as needed)** | **Specifications/Comments (e.g., if payments were made to you or to your institution)** |
| --- | --- | --- | --- | --- |
| **Time frame: Since the initial planning of the work** | | | | |
| **1** | All support for the present manuscript (e.g., funding, provision of study materials, medical writing, article processing charges, etc.)  **No time limit for this item.** | | \|  \| **None** \| \| --- \| --- \|  \| Taiwan National Science and Technology Council \| grant# NSTC-112-2314-B-002-216-MY3 \| \| --- \| --- \| \| Taiwan Ministry of Education \| grant# NTU-112L9004 \| \| Taiwan Centers for Disease Control \| grant# 114M071 \| | |
| **Time frame: past 36 months** | | | | |
| **2** | | Grants or contracts from any entity (if not indicated in item #1 above). | \|  \| **None** \| \| --- \| --- \|  \|  \|  \| \| --- \| --- \| \|  \|  \| \|  \|  \| | |
| **3** | | Royalties or licenses | \|  \| **None** \| \| --- \| --- \|  \|  \|  \| \| --- \| --- \| \|  \|  \| \|  \|  \| | |
| **4** | | Consulting fees | \|  \| **None** \| \| --- \| --- \|  \|  \|  \| \| --- \| --- \| \|  \|  \| \|  \|  \| \|  \|  \| | |
| **5** | | Payment or honoraria for lectures, presentations, speakers bureaus, manuscript writing or educational events | \|  \| **None** \| \| --- \| --- \|  \|  \|  \| \| --- \| --- \| \|  \|  \| \|  \|  \| | |
| **6** | | Payment for expert testimony | \|  \| **None** \| \| --- \| --- \|  \|  \|  \| \| --- \| --- \| \|  \|  \| \|  \|  \| | |
| **7** | | Support for attending meetings and/or travel | \|  \| **None** \| \| --- \| --- \|  \|  \|  \| \| --- \| --- \| \|  \|  \| \|  \|  \| | |
| **8** | | Patents planned, issued or pending | \|  \| **None** \| \| --- \| --- \|  \|  \|  \| \| --- \| --- \| \|  \|  \| \|  \|  \| | |
| **9** | | Participation on a Data Safety Monitoring Board or Advisory Board | \|  \| **None** \| \| --- \| --- \|  \| National Taiwan University Hospital and five allied medical centers or hospitals  (Investigator-initiated)  Multicenter Stepped Wedge Randomized Controlled Trial of Chlohexidine versus Beta-iodine for Prevention of Cathter-associated Urinary Tract Infection in Hospitalized Patients \|  \| \| --- \| --- \| \|  \|  \| \|  \|  \| | |
| **10** | | Leadership or fiduciary role in other board, society, committee or advocacy group, paid or unpaid | \|  \| **None** \| \| --- \| --- \|  \| Board Member in International Consortium:  The TB Contact Studies Consortium  Part of Analytic Consensus Team \|  \| \| --- \| --- \| \| Editorial Board of International academic  journals::  *Microbiology Spectrum* (American Society for Microbiology), 2022–2025  *Journal of Microbiology, Immunology and*  *Infection* (Infectious Disease Society of Taiwan), 2021–present  *Journal of the Formosan Medical Association*  (Formosan Medical Association), 2012–present \|  \| \|  \|  \| | |
| **11** | | Stock or stock options | \|  \| **None** \| \| --- \| --- \|  \| Photonics – AUO Corp (TPE 2409) \|  \| \| --- \| --- \| \| Computer – Quanta Computer Inc (TPE 2382) \|  \| \| Computer – Wistron Corp (TPE 3231) \|  \| \| Computer – Pegatron Corp (TPE 4938) \|  \| | |
| **12** | | Receipt of equipment, materials, drugs, medical writing, gifts or other services | \|  \| **None** \| \| --- \| --- \|  \|  \|  \| \| --- \| --- \| \|  \|  \| \|  \|  \| | |
| **13** | | Other financial or non-financial interests | \|  \| **None** \| \| --- \| --- \|  \|  \|  \| \| --- \| --- \| \|  \|  \| \|  \|  \| | |
|  | |  |  | |
| **Please place an “X” next to the following statement to indicate your agreement:** | | | | |
|  | | I certify that I have answered every question and have not altered the wording of any of the questions on this form. | | |


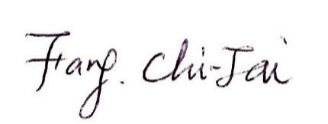

Supplement: ICMJE_CT Fang.docx [file TEMI_A_2654278_SM0931.docx]
